# Using inverse probability of censoring weighting to estimate hypothetical estimands in clinical trials: Should we implement stabilisation, and if so how?

**DOI:** 10.1177/09622802251387456

**Published:** 2025-10-31

**Authors:** Jingyi Xuan, Shahrul Mt-Isa, Nicholas R Latimer, Helen Bell Gorrod, William Malbecq, Kristel Vandormael, Victoria Yorke-Edwards, Ian R White

**Affiliations:** 1 MRC Clinical Trials Unit at UCL, 524254 University College London, London, UK; 2 Department of Infectious Disease Epidemiology and International Health, London School of Hygiene and Tropical Medicine, London, UK; 3 2793 Biostatistics and Research Decision Sciences (BARDS) Health Technology Assessment (HTA) Statistics, MSD, Zurich, Switzerland; 4 Sheffield Centre for Health and Related Research, School of Medicine and Population Health, University of Sheffield, Sheffield, UK; 5 Delta Hat Limited, Nottingham, UK; 6 Department of Mathematics, University of Brussels, Brussels, Belgium; 7 Biostatistics and Research Decision Sciences (BARDS) Health Technology Assessment (HTA) Statistics, MSD, Brussels, Belgium; 8 Centre for Advanced Research Computing, University College London, London, UK

**Keywords:** Inverse probability of censoring weight, stabilisation, propensity score, non-adherence, treatment switching, dependent censoring

## Abstract

Inverse probability of censoring weighting is an approach used to estimate the hypothetical treatment effect that would have been observed in a clinical trial if certain intercurrent events had not occurred. Despite the unbiased estimates obtained by inverse probability of censoring weighting when its key assumptions are satisfied, large standard errors and wide confidence intervals can be potential concerns. Inverse probability of censoring weighting with unstabilised weights can be simply implemented by calculating the reciprocal of the probability of being uncensored by the intercurrent events. To improve precision, stabilisation can be realised by replacing the numerator in the unstabilised weights with functions of the time and baseline covariates. Here, we aim to investigate whether stabilised weight is a preferred choice and if so how we should specify the numerator. In a simulation study, we assessed the performance of inverse probability of censoring weighting implementations with unstabilised weights and with different forms of stabilisation when the outcome analysis model was correctly specified or mis-specified. Scenarios were designed to vary the prevalence of the intercurrent event in one or both randomised arms, the existence of a deterministic intercurrent event, the indirect effect through baseline covariates and overall treatment effect, the existence and the pattern of time-varying effect and sample size. Results show that compared with unstabilised weights, stabilisation improves the efficiency of the inverse probability of censoring weighting estimator in most cases and the improvement is obvious when we stabilise for the baseline covariates. However, stabilisation risks increasing the bias when the outcome analysis model is mis-specified.

## 1. Introduction

Randomised controlled trials (RCTs) allocate participants to different intervention strategies, allowing the comparison of randomised groups with balanced characteristics. By convention, intention-to-treat (ITT) analyses based on the assignment of the interventions are usually conducted.^1–3^ However, a difference between the assignment and the receipt of the interventions is likely to be observed. Analysis based on the ITT principle ignoring those changes can still answer the clinical question focusing on the assignment to the interventions at baseline but fails to capture the effect that would be observed in the absence of events occurring post-randomisation.^4^ Our study focuses on intercurrent events (ICEs) defined as post-randomisation events that can ‘influence either the interpretation or the existence of the measurements associated with the clinical question of interest’.^5^ For example, patients may discontinue the assigned treatment, switch to another treatment either within or outside the trial, or receive additional treatment due to lack of efficacy, toxicity, or other reasons. When the occurrence of such events does not represent real-world practice or when the interest lies in isolating the effect of the treatment under investigation, a hypothetical strategy can be used to address these ICEs. A hypothetical estimand can be precisely defined by specifying the estimand attributes outlined in the ICH E9(R1) addendum.^5^ This study focuses on hypothetical estimands, with the aim of improving the performance of the estimators to target such estimands.

To address the ICEs with the hypothetical strategy, simple approaches exist but they require strong assumptions which are rarely satisfied in practice. Per-protocol (PP) attempts to remove the impact of ICEs and proceeds by setting data after ICEs to missing: it is easy to implement and is widely adopted in trials. However, when targeting a hypothetical estimand, PP is prone to selection bias when ICEs are caused by confounders which are also associated with the counterfactual outcomes.^ 6 ^ An extension to PP is to include the baseline covariates causing the ICEs in the substantive outcome model and we can obtain unbiased estimates when the ICEs are only caused by baseline confounders. We have to pay more attention when there are time-varying confounders (TVCs) defined as confounders that change over time in a manner which is not determined at baseline.^7^ TVCs are themselves mediators that are affected by prior treatment and also carry information on the indirect treatment effect. The outcome regression with baseline covariates does not suffice to control for all the confounding and we should not adjust for TVCs in a trial where our interest is the overall treatment effect. Therefore, in the presence of time-varying confounding, it is not suitable to adopt ordinary outcome regression.

More sophisticated statistical methods have been proposed to adjust for ICEs targeting a hypothetical estimand. A common type of approach used is to set the data post-ICEs to missing followed by the implementation of missing data methods. After excluding observations after ICEs, inverse probability of censoring weighting (IPCW) creates pseudo observations to represent the censored individuals,^8^ and multiple imputation (MI) creates hypothetical outcomes for censored individuals.^9^ These interpolating methods can adjust for time-varying confounding caused by TVCs making the artificial missingness occurring at random (given those confounders) more plausible. There are also methods developed for treatment switching, which have been used regularly in analyses submitted to health technology assessment agencies such as the National Institute for Health and Care Excellence (NICE). These methods include IPCW, as well as the rank preserving structural failure time model (RPSFTM) which models the effect of the intervention,^10^ and the two-stage method (TSE) which models the effect of the ICE.^11^

IPCW can be considered as an elaborate version of PP and is most closely related to standard analysis methods among those complex methods mentioned above. IPCW was shown by the authors to be a safer choice than PP in a simulation^12^ and increasing use of IPCW is expected when a hypothetical estimand is targeted. IPCW has straightforward implementation steps: following the same censoring process as in the simple PP analysis, extra weights are given to participants remaining uncensored. While the balance is broken between the groups in the PP population, the re-weighting process in IPCW results in a weighted sample of uncensored participants in each arm where the distribution of the measured covariates resembles that in the original trial population so that the balance (based on measured covariates) between randomised groups is recovered. IPCW yields unbiased estimates when the weighting model and the outcome model are correctly specified and the ‘No unmeasured confounders’ and ‘Positivity’ assumptions are satisfied.^13^ However, IPCW provides large standard errors (SEs) and wide confidence intervals especially when the weighting model performs poorly resulting in highly variable weights.^14^

Weights in the IPCW method can be unstabilised or stabilised.^13^ The unstabilised weight is obtained by calculating the reciprocal of the probability of remaining uncensored with the denominator informally seen as an individual’s conditional probability of remaining uncensored up to time t , given past time-dependent covariate history. Computed weights can be extreme for some participants and result in the IPCW estimator with unstabilised weights having a large variance. Stabilisation of weights was recommended to address this large variance and to obtain narrower 95% confidence intervals and better coverage rates when the outcome model is not saturated.^15^ For example, the numerator can be constructed to represent the marginal probability of remaining uncensored up to time t .

There is another form of stabilised weight where the numerator is the probability of remaining uncensored at a specific time point given the baseline covariates.^13^ As stabilising for baseline covariates re-introduces the association between baseline covariates and the ICEs, we have to adjust for those covariates in the outcome model (i.e. a marginal model additionally including baseline covariates). When we target a marginal treatment effect and the substantive outcome model is non-collapsible, standardisation is required after outcome modelling.

Though stabilisation is generally preferred in order to decrease the magnitude of the extreme weights calculated and reduce the numerical instability of small probabilities,^13,15^ the benefit and risk of stabilisation remain unclear in practice. In a review of methods addressing treatment switches in oncology trials, 1/6 of published articles stated the use of stabilised weights while 5/6 did not and 3/4 NICE Technology Appraisals used stabilised weights while 1/4 did not.^16^ In addition, the term ‘stabilisation’ was vaguely defined without a clear description of the form of the numerators.

When the outcome analysis model is not saturated, the choice of weights is driven by the goal of improving precision; however, it is equally important to assess their potential impact on accuracy. When we consider stabilising for time only, stabilisation mainly affects the relative weight given to different follow-up time points. This does not affect landmark survival obtained by the Kaplan-Meier (KM) estimator since the numerators are constant by arm and risk set but is problematic for estimands obtained by models assuming constant treatment effect over time. The substantive outcome model is supposed to be specified with underlying assumptions evaluated but in practice, it can be misspecified such that the treatment effect varies with time or with covariates (e.g. gender or age).

In this article, we investigate the implications of applying stabilised inverse probability weights when the substantive outcome model is mis-specified. Specifically, we consider a scenario where the true treatment effect varies over time, but the analysis assumes a constant effect. When stabilisation is implemented, weights computed are proportional to the time-specific information in the observations so that we can obtain more precise estimates than those obtained from unstabilised weights. But from an analysis with a mis-specified outcome model that does not account for the time-varying effect, the estimate is a weighted average of the time-specific treatment effects, which can be biased. To our knowledge, this is the first study to explore the potential for such bias due to mis-specified outcome models in the context of IPCW stabilisation.

Furthermore, while stabilisation of weights has been discussed in the context of marginal structural models for handling time-varying confounding^13,17^ and missing data,^18^ there has not been a systematic evaluation of stabilisation in IPCW in the context of RCTs. Our study fills this gap by providing a structured assessment of stabilisation strategies in RCTs under different outcome model specifications. We also consider practical situations that reflect common analysis choices in clinical trials. Important methodological issues are accounted for, including covariate adjustment in the substantive outcome model, the re-introduced confounding bias when stabilising for baseline covariates, and the need for appropriate adjustment to obtain the target estimand – particularly when it is non-collapsible.

The objective of this article is therefore to evaluate the performance of IPCW with unstabilised weights and stabilised weights in terms of accuracy and precision. To realise the objective, we start with an illustrative example with simplified settings. Then we conduct a simulation study covering potential IPCW implementations in a broad range of realistic trial settings. The article is organised as follows. In Section 2, we review the IPCW method and how different types of weights are typically implemented. In Section 3, we provide a simple example to explain the impact of stabilisation. In Sections 4 and 5, we report a series of simulation studies investigating the performance of IPCW with different forms of weights without stabilisation or with varied forms of stabilisation. We conclude in Section 6 with a discussion of the strengths, limitations and potential extensions of this study. We provide detailed information on the simulation design in Supplemental Material A and additional results of the simulation in Supplemental Materials B, C, D, E and F.

## 2. Overview of methods

### 2.1. Notation

This study is based on our previous work^12^ and we use similar notation. Let uppercase letters represent random variables, lowercase letters represent realisations of corresponding random variables or constants, Greek letters represent unknown parameters and overbars represent histories.

We use subscripts i to denote the values of the variables for patient i , labelled $i=1,\ldots ,n_{\mathrm{obs}}$ . We use subscripts v to represent baseline and follow-up visits, labelled v=0,1,…,V , where v=0 denotes the baseline and v=V denotes the last visit. Counterfactual variables are indexed with a superscript.

-
$B_{i}$ denotes the set of time-independent covariates measured at baseline v=0 .
-
$L_{iv}$ denotes the set of TVCs measured at follow-up visit v where v=0 (baseline), 1,2,… .
-
$Z_{i}$ is the binary variable for treatment allocation for participant i with 0 denoting the control group and 1 denoting the experimental group.
-
$C_{iv}$ denotes the binary variable for the occurrence of the ICE ( C for censoring) for participant i at visit v where v=0 (baseline), 1,2,… with 0 denoting the absence of the ICE and 1 denoting the presence of the ICE. $C_{ivto}=\sum_{t=1}^{v}C_{it}$ denotes the presence of ICE up until visit v . ${\bar{C}}_{iv}$ is the ICE history recorded for participant i at visit v and ${\bar{c}}_{0}$ denotes the history without ICE occurrence.
-
$T_{i\mathrm{off}}$ denotes the time (visit) to ICE for participant i .
-
$T_{iv\mathrm{on}}$ denotes the number of periods (intervals between visits) spent before the ICE for participant i until visit v .
-
$T_{iv\mathrm{off}}$ denotes the number of periods spent after the ICE for participant i until visit v .
-
$Y_{iv}$ denotes the outcome variable for participant i at visit v .
-
$Y_{iv}^{Z_{i},{\bar{C}}_{iv}}$ denotes the potential outcome of participant i under treatment history ( $Z_{i},{\bar{C}}_{iv}$ ).
-
$K_{iv}$ denotes the set of covariate information that we select to include in the model from the available data for covariates at visit v . Depending on the model assumptions, this can possibly include non-linear and/or interaction terms, at visit v and up until visit v (i.e. a measurement history).
-
$X_{i}$ denotes the set of baseline measurements of the selected covariates $K_{i}$ . That is to say, $X_{i}$ is a subset of $\left(B_{i},L_{i0}\right)$ .

### 2.2. Hypothetical estimand

The hypothetical estimand targeted by IPCW compares the effect of hypothetical treatment strategies under investigation. In this study, we are interested in comparing two randomised groups in a trial if the ICEs specified with the hypothetical strategy had not occurred. We define the treatment strategy for the control group as ( $Z=0,{\bar{c}}_{0}$ ) and the treatment strategy for the experimental group as ( $Z=1,{\bar{c}}_{0}$ ).

Targeting a causal effect, we focus on comparing the counterfactual outcomes $Y_{v}^{z_{0},{\bar{c}}_{0}}$ and $Y_{v}^{z_{1},{\bar{c}}_{0}}$ . Such a binary outcome of interest denotes both whether an event occurred and when it occurred so it has a TTE nature. We consider the effect measures to characterise the causal effect which is given by the distribution of counterfactual effect in the trial population for every participant. Since forms of stabilisation that include baseline covariates in the numerator require covariates to be adjusted in the substantive outcome model (see Section 2.3.2), collapsibility is considered while choosing a suitable effect measure. Therefore, we use marginal risk difference at a specified time endpoint as the estimand for all methods.

### 2.3. IPCW estimator

Here we discuss how to conduct the statistical analysis process with an IPCW estimator given a well-defined estimand. To align with the simulation study to be introduced in Section 4, we describe the implementation process considering a trial with TTE outcome, with data on covariates, ICEs and outcome status measured at discrete times (follow-up visits).

#### 2.3.1. Censoring by ICEs

In common with PP analysis, IPCW first censors the observations by the occurrence of ICEs. It is a selection process that removes the effect of ICEs but breaks the balance of characteristics between randomised groups.

#### 2.3.2. Modelling ICEs and calculating inverse probability weights

In order to recover the balance between intervention strategies under investigation, weighting is used to remove the association between the occurrence of the ICEs and the confounders. In brief, weighting is realised by modelling the ICE mechanism and then giving extra weights computed by the inverse probability of remaining uncensored to the uncensored participants.

First, we need to create the probability model for the occurrence of the ICE. We consider cases where ICEs occur in one arm only or in both arms but here we only show the process of weighting in one arm. Under the cases where ICEs occur in both arms, rather than modelling all ICEs using a single model, we model the ICE separately for each randomised arm. Although it doubles the number of parameters potentially reducing the efficiency, it reduces the risk of model mis-specification potentially missing arm-related interaction terms. Instead of estimating a separate intercept for each visit, we assume the intercept to be a smooth function and use pooled logistic regression to estimate the inverse probability weight.^13^

In terms of different forms of weights, we provide a detailed modelling process of the weighting forms under investigation in this study, as follows:

- Unstabilised weight:
  Unstabilised weighting is the simplest form of the weighting process and we need to estimate the denominator of the weight (while the numerator is one) for each subject and visit. For each arm, we fit a pooled logistic regression model:
(1) $$\mathrm{logit}P(C_{iv}=1|C_{i(v-1)}=0,K_{iv},Z_{i}=z)=\vartheta_{z}+\zeta_{z}K_{iv}+\eta_{z}f_{c}(v)$$
  where z=0,1 and $f_{c}(v)$ is a function of visit v .
  After that, we predict the probability of occurrence of ICE for individual i at visit v :
(2) $${\hat{\pi }}_{Div}=\hat{P}[C_{iv}=1|C_{i(v-1)}=0,K_{iv},Z_{i}]$$
  where ${\hat{\pi }}_{Div}$ denotes the probability predicted to be used in the denominator.
  Then the unstabilised weight of individual i at visit v can be constructed by multiplying together all their probabilities of not having ICEs up to visit v :
(3) $${\hat{W}}_{iv}=\prod_{k=1}^{v}\frac{1}{1-{\hat{\pi }}_{Dik}}$$
- Stabilised weight:
  When components of the X are strongly associated with $C_{iv}$ , the probabilities $pr[C_{iv}=0|X=x_{i}]$ can vary greatly between participants. As a result, we may obtain extreme weights for some participants.^15^ The variation in the unstabilised weights may get larger as time accumulates in sustained treatment cases. We can compute stabilised weight by changing the numerator from 1 in equation (3) into the probability of a participant remaining uncensored by the ICE at visit v , conditional on a selection of variables. The stabilised weights $SW=\frac{g[C]}{f[C|K]}$ have multiple forms with a selection of different specifications of the numerator, where g[C] needs to be a function of v and X .
  When the key assumptions (see Section 2.4) required by the IPCW method are satisfied, implementations with unstabilised or stabilised weights result in point estimates that are the same in expectation but stabilised weights typically result in narrower 95% confidence intervals than unstabilised weights. However, the statistical superiority in the precision of the stabilised weights can only occur when the outcome model is not saturated. When saturated models are specified, the treatment effect estimates that result from the use of stabilised or unstabilised weights are the same.^15^ In correctly specified unsaturated outcome models, the estimates differ but this is only because of the sampling variability.^19^ Since in this study we have sustained treatment with the time-varying occurrence of ICEs, the weighted model cannot possibly be saturated and therefore stabilised weights are used. In other settings (e.g. continuous treatments), the weighted outcome model is also unsaturated.
  We consider varied forms of the stabilised weights and classify them into the following two types. The construction of the numerator is realised in a similar process by obtaining the prediction of the probability after fitting a pooled logistic regression with the set of variables included in the numerator.
  - Stabilised weight without baseline covariates
    First, we estimate the probability of occurrence of ICE for individual i at visit v given that no ICEs occurred in the last visit:
(4) $${\hat{\pi }}_{Niv}=\hat{P}[C_{iv}=1|C_{i(v-1)}=0,Z_{i}]$$
    where ${\hat{\pi }}_{Niv}$ denotes the probability predicted with the above model without baseline covariates to be used in the numerator. The estimates can be obtained by fitting a pooled logistic model with time to ICEs only or non-parametrically through the KM estimator of time to ICEs. Then obtain the stabilised weight by the following equation:
(5) $${\hat{\mathrm{SW}}}_{iv}=\prod_{k=1}^{v}\frac{1-{\hat{\pi }}_{Nik}}{1-{\hat{\pi }}_{Dik}}$$
  - Stabilised weight with baseline covariates
    We can also include baseline covariates and the time in the numerator. The probability of occurrence of the ICE at visit v given baseline covariates is estimated after fitting a pooled logistic model with time to ICE and baseline covariates:
(6) $${\hat{\pi }}_{NBiv}=\hat{P}[C_{iv}=1|X_{i},C_{i(v-1)}=0,Z_{i}]$$
    We obtain the stabilised weight by the following equation:
(7) $${\hat{\mathrm{SW}}}_{Biv}=\prod_{k=1}^{v}\frac{1-{\hat{\pi }}_{NBik}}{1-{\hat{\pi }}_{Dik}}$$
    where ${\hat{\pi }}_{NBiv}$ denotes the probability predicted with the above model baseline covariates to be used in the numerator.

#### 2.3.3. Modelling and estimating the outcome

The specification of the outcome model is completed in the statistical analysis plan according to the trial objective of interest and plausible assumptions about the survival curve based on background knowledge. Given that in this study we are interested in a TTE outcome, the modelling of the outcome should consider the plausibility of the treatment effect over time. The time-varying property of the treatment effect should be sufficiently accounted for.

In the context of inverse probability method implementation, the outcome model usually refers to a marginal model without covariate adjustment, but a more general outcome can be specified for two aims. One case is when covariate adjustment is planned to improve efficiency, covariate-adjusted outcome regression may be conducted as one of the ways to realise covariate adjustment.^20,21^ Another case is when we have a particular interest in a conditional treatment effect or typically we want to identify treatment effect heterogeneity (TEH), we will include some baseline variables and/or relative interaction terms forming a general form of marginal outcome model. This targets a different estimand not covered here.

We assume that the trial’s analysis plan specifies a regression model for Y with Z and X as covariates. This could be a fully parametric model or a semiparametric model, but we require that it at least specifies how the mean of Y depends on Z and X :

(8) $$E(Y_{v}|Z,X)=f(Z,X,\beta ,v)$$

where f(Z,X,β) is an assumed function with β being the model parameters.

To estimate the outcome using IPCW, common methods in survival analysis including the KM estimator, log-rank test, or Cox partial likelihood estimator of the ratio of the treatment–arm-specific mortality rates, can be adopted with their weighted versions.^8,22^ When baseline covariates are adjusted in the numerator (see equation (6)), bias induced by baseline covariates is re-introduced so we have to adjust for these covariates in the outcome model. If the pre-specified outcome model does not have baseline variables, the outcome model will need to be updated by adding the baseline covariates that are included in the numerator or stabilisation using (7) should be abandoned. In addition, a standardisation process is needed after fitting the updated outcome model to avoid the shift of estimand if we are interested in estimating a marginal treatment effect (which is the case under investigation in this study).

#### 2.3.4. Variance estimation

Ordinary standard errors fail to account for the induced correlation among weighted observations so they are not valid in a weighted analysis. The sandwich variance or the delta method can be implemented but does not incorporate uncertainty in estimating the weights and tends to underestimate the variance. Bootstrapping accounts for the uncertainty in weight estimation and is thought to be more conservative. Therefore, we use bootstrapping for variance estimation in this study.

### 2.4. Assumptions

#### 2.4.1. No unmeasured confounding

No unmeasured confounding (NUC) is assumed by the IPCW, requiring that all confounders (i.e. variables that are common causes of the counterfactual outcome and the ICEs) are identified and properly adjusted so that the association between confounders and ICEs is fully removed. Since we have no information about the confounders that are not measured, this assumption is not statistically testable.^23,24^ Under the cases where sustained treatment strategies are being investigated and ICEs occur at different times, potential confounders should include both the time-invariant ones measured at baseline (e.g. age or gender) and time-varying ones measured at follow-up visits (e.g. an indicator of disease progression). For confounder selection, the structural causal approach using the directed acyclic graph (DAG) at the trial design stage may be the preferred choice rather than a data-driven method at the analysis stage which can possibly hide important causal relationships.^25^ Sensitivity analysis is also suggested; by adding measured variables one at a time and comparing the estimate obtained, we attempt to adjust for the bias caused by measured confounders while controlling for the variance to be at a reasonable level.

#### 2.4.2. Positivity

When the probability of remaining uncensored by ICEs equals 0 for some combinations of covariates, the positivity assumption is violated. A structural violation occurs when theoretically some participants with specific covariate values are certain to have ICEs and this can not be addressed by increasing the sample size. Even with no structural violation, practical violations can occur by chance in finite samples.^26^ The positivity assumption is required as the IPCW estimator is undefined in the presence of non-positivity and there would be no participants with similar characteristics left to reweight.^15,27^

#### 2.4.3. Correct model specification

For IPCW to produce valid estimates, the method requires correct specifications of both the weighting model and the outcome model.^27^ There are two main types of model mis-specification. One is the mis-specification of the main effect of the covariates or time (e.g. missing non-linear term) and the other is the omission of potential interaction terms. For the weighting model, model mis-specification mainly refers to missing confounders or omitting non-linear terms and interaction terms potentially resulting in the violation of the NUC assumption mentioned above. If the outcome model is mis-specified (which is the focus of this study), such that the treatment effect varies with time or with baseline covariates, then IPCW may fail to provide an unbiased estimate.

## 3. An illustrative example

In this section, we use a simple example to illustrate the potential extra loss in accuracy brought up by stabilisation when the substantive outcome model is mis-specified.

We consider a two-arm randomised trial with N participants following 1:1 randomisation ( $n_{0}=n_{1}=\frac{N}{2}$ ) and the true treatment effect varies over time. For simplicity, we include two follow-up visits (at month 1 and month 2) and an ICE which occurs between the first and the second visit in the control arm. Information on the ICE (and covariates) is collected up until the second visit and the outcome (which is irreversible) status is observed at two visits. Given that the same denominator is used for unstabilised and stabilised weights, the selection bias caused by censoring the patients with the ICE is accounted for in the same way by IPCW with both forms of weights. Therefore, we consider the special case in the absence of confounders as confounding is irrelevant to the issue that we focus on.

**Figure 1.**
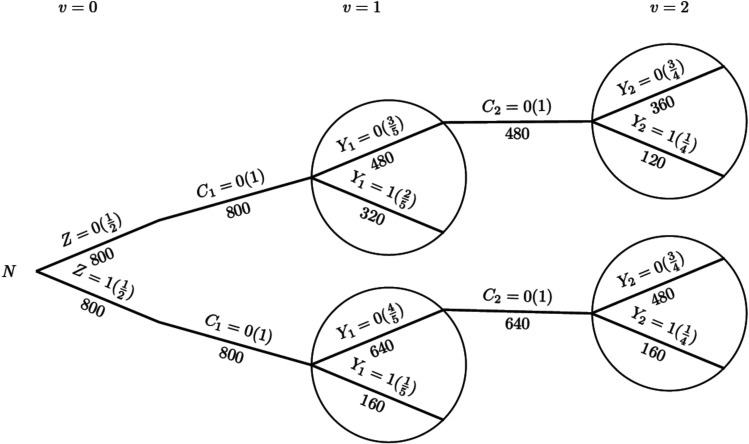
Probability tree illustrating the hypothetical case without an intercurrent event. *Note:* Labels in the brackets show the conditional probability of each value of the variables given all prior branches and labels below branches represent the observed number of individuals for those values of the variables.

**Figure 2.**
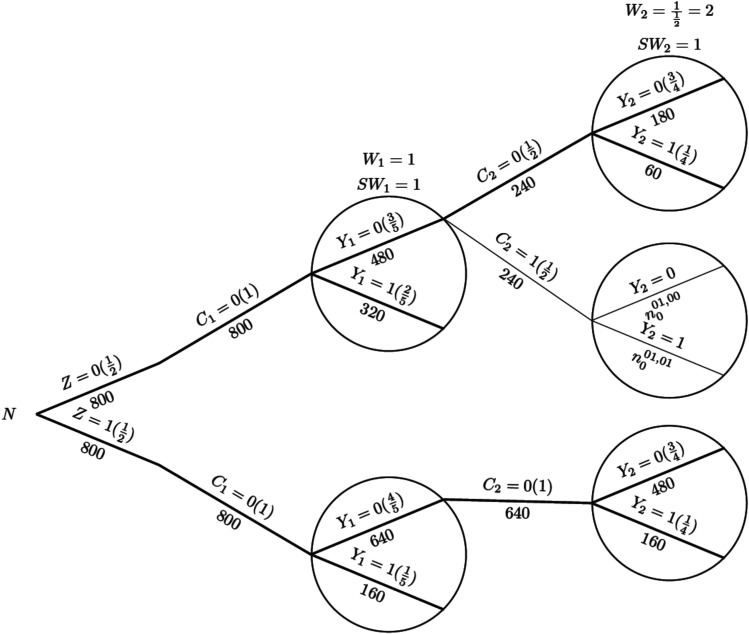
Probability tree illustrating the case where there is an intercurrent event in the control arm, with weights applied in the control arm. *Note:* Labels in the brackets show the conditional probability of each value of the variables given all prior branches and labels below branches represent the observed number of individuals for those values of the variables; v = visit ( v=0,1,2 ); $W_{v}$ = unstabilised weights calculated at visit v ; $SW_{v}$ = stabilised weights calculated at visit v .

To illustrate the process of IPCW implementation with unstabilised or stabilised weights, we show each component required for the computation using a probability tree,^28,29^ which includes all potential combinations of the treatment arm, status of ICE occurrence, measured variables and outcome status up to the second visit ( v=2 ). We let the ordering of nodes from left to right denote the ordering of time for observations. We use circular nodes for the outcome variable and use tree branches outside circular nodes to represent randomised treatment and the time-varying occurrence of the ICE. Labels in the brackets show the conditional probability of each value of the variables given all prior branches and labels below branches represent the observed number of individuals for those values of the variables. Figure 1 shows the hypothetical scenario in the absence of an ICE and Figure 2 shows a scenario with an ICE occurring in the control arm. For the hypothetical estimand which targets the treatment effect that would be observed had the ICE not occurred, we compare two treatment strategies which are being assigned the experimental treatment and receiving the assigned control treatment in the absence of the ICE. We use bold lines to denote the branches from randomisation representing those treatment strategies of interest.

For the IPCW implementation process, we use the probability tree to illustrate (see Figure 2). The first step is to censor observations at the occurrence of the ICE and it is conducted by only keeping the branches in bold (following $C_{2}=0$ ) while dropping the other branches (following $C_{2}=1$ ). Then we give weights to the outcome node on the branch in bold at each time point. The unstabilised weight given to individuals remaining uncensored is 1 at v=1 and $\frac{1}{\frac{1}{2}}=2$ at v=2 . Stabilised weights are both 1 for v=1,2 in this special case. Following the weighting process, we conduct the outcome analysis. We assume a constant treatment effect (i.e. using a mis-specified outcome model) and target a hazard ratio (HR).

For the outcome analysis, we assume that the hazard rate is constant over time (e.g. in an exponential survival model) to simplify the explanation. Let $\lambda_{0}$ denote the control group hazard rate and $\lambda_{1}$ denote the experimental group hazard rate.

Given the survival data over two time periods, we obtain the total person-time for each group, assuming patients remain at risk throughout the time period until they experience an event or are censored. We start with the hypothetical scenario in the absence of the ICE as shown in Figure 1.

For the control group, the constant hazard rate (events per person-month) is:

(9) $$\lambda_{0}=\frac{{\text{Total number of events}}_{\mathrm{control}}}{{\text{Total person-months at risk}}_{\mathrm{control}}}=\frac{320+120}{800+480}=\frac{440}{1280}\approx 0.344$$

For the experimental group, the hazard rate is:

(10) $$\lambda_{1}=\frac{{\text{Total number of events}}_{\mathrm{experimental}}}{{\text{Total person-months at risk}}_{\mathrm{experimental}}}=\frac{160+160}{800+640}=\frac{320}{1440}\approx 0.222$$

The hazard ratio is:

(11) $$\mathrm{HR}=\frac{\lambda_{1}}{\lambda_{0}}=\frac{0.222}{0.344}\approx 0.646$$

Now we consider the situation where an ICE is introduced as shown in Figure 2. Because the ICE only occurs in the control arm, the hazard rate for the experimental group (i.e. the numerator in equation (11)) is the same for all methods. Therefore, we can focus on the denominator and estimate the control group hazard rate and the incorrect model assumption has become ‘hazard rate is independent of time in the control arm’.

Table 1 shows the observed and weighted number of events and person-months at risk by different methods. For the second time interval, events/person-months is 120/480 in a hypothetical scenario without an ICE and events/person-months is observed as 60/240 in the scenario with an ICE. While weighting the observations with unstabilised weights effectively provides the correct events/person-months =120/480 , events/person-months remains the same as observed ( 60/240 ) using stabilised weights.

**Table 1.** Summary of observed and weighted events and person-months in two time intervals in the control group.

	Up to v= 1			Between v= 1 and v= 2	
Scenario	Events	Person-months		Events	Person-months
Observed data
Without an ICE	320	800		120	480
With an ICE	320	800		60	240
With an ICE, weighted data			Weight
Unstabilised weight	320	800	2	120	480
Stabilised weight	320	800	1	60	240

ICE: intercurrent event.

The hazard rate in the control group obtained from the outcome model weighted by unstabilised weight is

(12) $$\lambda_{0W}=\frac{{\text{Total number of events}}_{\mathrm{control}}}{{\text{Total person-months at risk}}_{\mathrm{control}}}=\frac{320+120}{800+480}=\frac{440}{1280}\approx 0.344$$

and by stabilised weight is

(13) $$\lambda_{0\mathrm{SW}}=\frac{{\text{Total number of events}}_{\mathrm{control}}}{{\text{Total person-months at risk}}_{\mathrm{control}}}=\frac{320+60}{800+240}=\frac{19}{52}\approx 0.365$$

With the hazard rate for the experimental group ( $\lambda_{1}=0.222$ from equation (10)), HR is $\frac{0.222}{0.344}\approx 0.646$ by unstabilised weight and $\frac{0.222}{0.365}\approx 0.608$ by stabilised weight.

From the computed HR by two forms of IPCW, we observe that only IPCW with unstabilised weights gives the correct average 0.646 which is the same as that provided by the true HR in the case where there is not an ICE. However, IPCW with stabilised weights gives an overestimated hazard rate for the control group and in turn an exaggerated treatment effect (HR =0.608 ). The bias arises because the stabilised weight underweights visit 2, at which there is in fact no treatment effect but at which the estimation assumes the same treatment effect as at visit 1. This bias will grow as the prevalence of ICE increases or as the treatment effect changes more significantly over time. While in this example we use the simplest modelling assuming a constant hazard rate, the analysis adjusting for time shows similar findings.

## 4. Simulation study

We use a simulation study to investigate the performance of IPCW implementations with different forms of weights to estimate a hypothetical estimand. The simulation study is informed by ODYSSEY (a trial in paediatric HIV infection) in order to emulate a realistic trial setting.^30^ We used a similar setting as we did in a previous simulation study based on ODYSSEY.^12^ Properties of TVCs, ICEs and outcomes were explored to choose parameters for the data-generating mechanism. The following sections begin with a description of the design of the simulation according to the ADEMP framework by Morris et al.^31^ We then summarise and interpret the main results and follow with some useful conclusions at the end.

### 4.1. General setting

**Figure 3.**
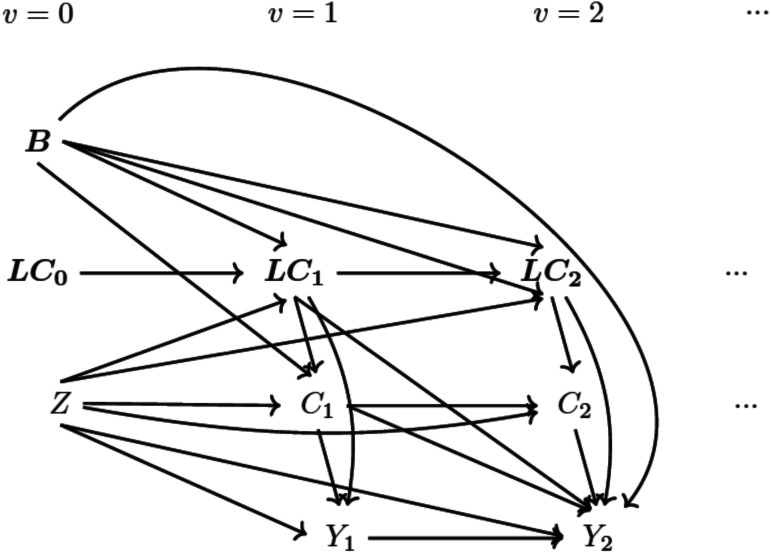
Directed acyclic graph (DAG) for data-generating mechanism *Note:* (1) Z represents the randomised treatment and B represents time-independent covariates measured at baseline. (2) Suppressing i for the variables shown in Section 2.1, $C_{v}$ represents the occurrence of the intercurrent event (ICE) at visit v and $LC_{v}$ represents confounders at visit v . (3) Risk factors $LR_{v}$ and ICE factors $LI_{v}$ at visit v which are not confounders are omitted here for clearer illustration.

We simulate a trial with a sustained treatment strategy and time-varying occurrence of one ICE in one or both arms. Both the baseline and the time-varying covariates influence the occurrence of the ICE and there is time-varying confounding. We focus on a case where every trial participant is observed at regular time points (e.g. follow-up visits) v=1,2,… up to the event or trial termination. Time-varying covariates $L_{iv}$ and the indicator of ICE occurrence $C_{iv}$ are recorded at every visit. Events and the occurrence of the ICE are assumed to be observed in continuous time. We assume all participants start the assigned treatment $Z_{i}$ at v=0 but they may follow a range of longitudinal ICE patterns thereafter, with the pattern depending on both baseline and time-varying covariates. The ICE is assumed to occur from the first follow-up visit until the seventh visit v=1,2,…,7 but not at the last visit v=8 since the occurrence at this time does not influence the outcome. No loss to follow-up and no measurement errors are also assumed. The assumed structure of the data is illustrated in the DAG in Figure 3.

### 4.2. Aim

The aim of this study is to evaluate the performance of IPCW implemented with different forms of weights targeting a hypothetical estimand in a series of situations that are likely to occur in practice potentially violating the underlying model assumptions.

### 4.3. Data-generating mechanisms

Starting with a description of supplementary notations, we introduce the framework for the simulation process covering all models with mathematical details followed by the specification of varying factors under exploration for scenario designs.

#### 4.3.1. Updated notations

Based on the notations described in Section 2.1, here we add further details. Vector of baseline covariates $B_{i}$ has three components including baseline age ( $B_{i1}$ ), gender ( $B_{i2}$ ) and WHO stage ( $B_{i3}$ ). Vector of TVCs measured at each visit v denoted as $L_{iv}$ has five components including logarithm of CD4 count ( $L_{iv1}$ ), logarithm of CD4/CD8 ratio ( $L_{iv2}$ ), viral load ( $L_{iv3}$ ), total cholesterol ( $L_{iv4}$ ) and triglycerides ( $L_{iv5}$ ). Therefore, sets of TVCs, ICE factors and outcome risk factors are defined as ( $LC_{iv}=\{L_{iv1},L_{iv2}\}$ ), $LR_{iv}=\{L_{iv3}\}$ , $LI_{iv}=\{L_{iv4},L_{iv5}\}$ ).

#### 4.3.2. Data-generation process

The simulating process is designed in a setting where $n_{\mathrm{obs}}$ participants enter the trial at baseline ( v=0 ) with baseline measurements $X_{i}$ and are randomly assigned to intervention $Z_{i}$ . Then at each of the following visits, data are simulated in a sequential conditional manner with time-varying covariates ( $L_{iv}$ ), the occurrence of the ICE ( $C_{iv}$ ) and outcome status ( $Y_{iv}$ ) at each visit generated in turn conditional on the previous values, starting from the baseline visit. Figure 3 visualises this process using a DAG with non-confounding time-varying covariates omitted. Data are generated from left to right following the time sequence and from top to bottom according to the mechanism described so far. The detailed generating algorithm is described in Supplemental Material A.1.

1. Baseline measurement model: Values of covariates except for $B_{i2}$ at v=0 are generated from a truncated multivariate normal distribution. $B_{i1},B_{i3},L_{i01},L_{i02},L_{i03},L_{i04},L_{i05}∼TruncNorm(\mu_{0},\Sigma ,m,n)$ . Then simulate the binary $B_{i2}$ with $B_{i2}∼Bernoulli(logit^{-1}(\rho_{1}B_{i1}+\rho_{2}B_{i3}+\rho_{3}L_{i0}))$ .
2. Randomised treatment model: Randomly allocated treatment is generated using $Z_{i}∼Bernoulli(0.5)$ which denotes a 1:1 randomisation to the control and the experimental groups.
3. TVC model: The values of TVCs are simulated visit by visit depending on treatment history, visit and covariates history.
  The values of TVCs are generated at each follow-up visit v :
(14) $$\begin{array}{rl} L_{ivq} & =\beta_{q0}+\beta_{q1}Z_{i}+(\beta_{q2}+\beta_{q3}Z_{i})C_{i(v-1)to}+\beta_{q4}B_{i}+\beta_{q5}L_{i(v-1)q}+\epsilon_{iq} \end{array}$$
(15) $$\begin{array}{rl} \epsilon_{i} & ∼Norm(\xi ,\Lambda ) \end{array}$$
  where v=1,…,8 , $i=1,\ldots ,n_{\mathrm{obs}}$ and q=1,…,5 . $\epsilon_{i}$ is the vector of error terms for $L_{ivq}$ with mean vector ξ and covariance matrix (for q=1,2 ) Λ . $L_{1}$ and $L_{2}$ are made correlated by the error terms $\epsilon_{i}$ , rather than by letting each L depend on lagged values of other L ’s. The correlated error terms for each participant at each visit are realised by using Cholesky factorisation. The correlation matrix denoted as M of the error terms of time-dependent confounders is designed to be fixed across time.
4. ICE model: Let $p_{iv}$ denote the probability of ICE for participant i in the interval [v,v+1) . The indicator of ICE for participant i at visit v is generated :
(16) $$\begin{array}{rl} p_{iv} & =logit^{-1}(\theta_{0}+\theta_{1}v+\theta_{2}Z_{i}+\theta_{3}B_{i}+\theta_{4}L_{iv})\\ C_{iv} & ∼Bernoulli(p_{iv})\\ & if\;C_{i(v-1)}=0 \end{array}$$
$\theta_{2}$ controls for the difference of ICE occurrence in two randomised groups. $\theta_{3}$ is a vector of parameters for baseline covariates and $\theta_{3}=(\theta_{31},\theta_{32},\theta_{33})$ . $\theta_{4}$ is a vector of parameters for time-dependent covariates and $\theta_{4}=(\theta_{41},\theta_{42},\theta_{43},\theta_{44},\theta_{45})$ . Note that time-dependent covariates in the ICE model include two confounders ( $L_{1}$ and $L_{2}$ ) and two non-confounding factors ( $L_{4}$ and $L_{5}$ ) associated with ICE. Non-confounding risk factor ( $L_{3}$ ) is not included in the model since it is not associated with ICE ( $\theta_{43}=0$ ).
5. Outcome model: Since we assume discrete-time data, we can work with the probability of failure in a single time interval [v,v+1) given survival up to visit v , as the discrete equivalent of the hazard function. Let $\lambda_{iv}$ denote the probability of failure in the interval [v,v+1) conditional on survival up to visit v ,
(17) $$\begin{array}{rl} \lambda_{iv}= & logit^{-1}(\mu_{0}+\mu_{1}Z_{i}+(\mu_{2}+\mu_{3}Z_{i})T_{ivon}\\ & +(\mu_{4}+\mu_{5}Z_{i})T_{ivoff}+\mu_{6}B_{i}+\mu_{7}L_{iv})\\ Y_{iv}∼ & Bernoulli(\lambda_{iv}) \end{array}$$
  where v=1,2,…,8 , $i=1,2,\ldots ,n_{\mathrm{obs}}$ , $T_{iv\mathrm{on}}=\min(v,T_{i\mathrm{off}})$ and $T_{iv\mathrm{off}}=\max(v-T_{ioff},0)$ . Time-dependent non-confounding covariates $L_{4}$ and $L_{5}$ are not included in the outcome model since they are not associated with the outcome ( $\mu_{74}=\mu_{75}=0$ ).

#### 4.3.3. Scenario design

For the purpose of scenario design, while fixing some parameters in the models in the dynamic general model (DGM), we use a full factorial design in which the factors with multiple levels are allowed to vary (see Table 2). For the ICE mechanism, we vary the arm with the ICE and the pattern of ICE covering different prevalences of ICE and structural violations of positivity. In the outcome model, the magnitude of the indirect effect carried by baseline covariates and the magnitude of the overall treatment effect are varied. The treatment effect is designed to be time-invariant or time-varying with early or late effects. We also consider sample sizes of 500 or 1000 considering small sample bias (practical violation of the positivity assumption) and imprecision. In total, 144 scenarios are investigated.

**Table 2.** Summary of quantities specified in the DGM for a factorial simulation design.

Factor	Levels	Description
TVC model		
Confounders	1	$L_{1}$ and $L_{2}$
Risk factor	1	$L_{3}$
ICE factors	1	$L_{4}$ and $L_{5}$
Correlation between confounders	1	0.4
ICE model
Arm of ICE occurrence	2	Control arm/both arms
ICE characteristics	3	prevalence of 0.4 prevalence of 0.6
		prevalence of 0.4 with deterministic occurrence ${}^{a}$
Outcome model
Indirect treatment effect by baseline covariates	2	Medium/high
Treatment effect over time	3	Constant/increasing/decreasing
Overall treatment effect	2	Low/ medium
Trial setting
Sample size	2	500, 1000

*Note:* DGM: data-generating mechanism; TVC: time-dependent covariates; ICE: intercurrent event.

${}^{a}$ To account for the case where the positivity assumption is violated, ICE is certain to occur when the values of time-varying covariates reach a pre-specified level.

Following the identified factors described above, this simulation is implemented in a factorial exploration process with all scenarios summarised in Tables A1 and A2 in Supplemental Material A. For the choice of parameters in the models included in the data-generating mechanism, we refer to the properties obtained in the ODYSSEY data. Supplemental Material A.3 shows numerical values for varied parameters in each scenario.

### 4.4. Estimand

#### 4.4.1. Marginal risk difference

We use marginal risk difference as introduced in Section 2.2, and set the analysis time to be at the eighth visit ( v=8 ). Therefore, the estimand for all methods is:

(18) $$RD(8)=Pr(Y_{8}^{z_{1},{\bar{c}}_{0}}=1)-Pr(Y_{8}^{z_{0},{\bar{c}}_{0}}=1)$$

#### 4.4.2. True value of the estimand

Since the true value of the estimand can not be obtained directly in the data-generating models, we use an alternative approach by estimating from a large simulated dataset.^32^ We set a sufficiently large dataset with the size of $2\times 10^{6}$ so that the variance in the truth calculation is ignorable. By simulating hypothetical intervention regimes in the absence of ICEs and the counterfactual outcome under this case, we obtain the ‘true’ risk difference.

### 4.5. Method

IPCW with varying forms of implementation is explored in this simulation. The method is implemented as described in Section 2.3, with varying factors in both the weighting model and the outcome model, as shown in Table 3.

**Table 3.** Summary of factors varying in IPCW implementations.

	Factor	Values	Description	Reason ${}^{b}$
Weighting model	Numerator	1	Unstabilised
		v	Stabilised without baseline covariates	Main aim
		v , $X_{i}^{a}$	Stabilised with baseline covariates
	Denominator	v , $K_{iN}$	All confounders included (NUC)	Sensitivity
		v , $K_{iR}^{a}$	Omitting a confounder (RC)	
Outcome model	Time-varying treatment effect	trt*v	Assuming time-varying treatment effect	Main aim
		–	Assuming constant treatment effect
	Covariate adjustment	$X_{i}$	Analysis adjusted for baseline covariates	Sensitivity
		–	Analysis not adjusted for baseline covariates

*Note:* IPCW: inverse probability of censoring weighting; NUC: no unmeasured confounding; RC: residual confounding.

${}^{a}$ For NUC: $K_{iN}=(B_{i},L_{i1},L_{i2})$ and $X_{i}=(B_{i},L_{i01},L_{i02})$ ; for RC: $K_{iR}=(B_{i},L_{i2})$ and $X_{i}=(B_{i},L_{i02})$ .

${}^{b}$ We mainly focus on investigating the numerator and we control for it by varying it with different forms. The other factors are treated as pre-specified and outside the scope of the comparison in this study. We also varied these factors in sensitivity analyses to support our conclusion.

#### 4.5.1. Handling the ICE

Following censoring by the ICE, models for the numerator and the denominator are specified and then the inverse probability weights are calculated. The numerator is the main focus of this study and is varied by replacing 1 in the numerator of the unstabilised weights with different variables including time only, or baseline covariates and time together (see Table 3). The specification of the denominator is the key to satisfying the NUC assumption and in the main analysis of this study we assume a correctly specified denominator with no residual confounding. We also explore the situations with residual confounding, realised by omitting one confounder for sensitivity.

#### 4.5.2. Modelling and estimating the outcome

We assume that the trial’s SAP specifies a regression model for Y with Z and X as covariates. As described in Section 2, we considered situations where covariate adjustment is pre-specified or not and with time-varying/ time-invariant treatment effect.

Calculated weights are incorporated within the pooled logistic regression to model the binary outcomes measured across follow-up visits. The parameters of the pooled logistic regression are known to approximate the parameters of a Cox proportional hazard model when the outcome is rare in each time interval.^33^ Since our aim is to compare unstabilised weights with stabilised weights rather than compare different outcome analyses, we assume that the outcome model is pre-specified (with a determined choice of whether to account for the time-varying treatment effect and whether to do covariate adjustment) before the analysis. The model described by equation (19) shows the simplest form of the outcome model when a time-invariant treatment effect is assumed and when no covariate adjustment is conducted nor baseline covariates are included in the numerator of the weighting model. A product term between randomised arm $Z_{i}$ and a function of time is added to the model if a time-varying treatment effect is assumed. The outcome model may be pre-specified to include baseline covariates for specific estimand or covariate adjustment. Also, when the stabilised weight with baseline covariates is included in the numerator, those covariates are included in the outcome model as well to eliminate the re-introduced bias by the numerator (see equations (20) for a time-invariant treatment effect and (21) for a time-varying treatment effect). Assuming no selection bias due to loss to follow-up or measurement error, unbiased estimates can be yielded by fitting one of the following logistic regression models (equations (19)–21(22)) considering the model assumptions. Depending on the true outcome model mechanism in the DGM among scenarios, these potential implementations of the outcome models are therefore correctly specified or mis-specified.

(19) $$\begin{array}{rl} logit[Pr(Y_{iv} & =1|Z_{i},Y_{i(v-1)}=0,C_{iv}=0)]=\alpha Z_{i}+\gamma f(v) \end{array}$$

(20) $$\begin{array}{rl} logit[Pr(Y_{iv} & =1|Z_{i},Y_{i(v-1)}=0,C_{iv}=0)]=\alpha Z_{i}+\gamma f(v)+\psi Z_{i}f(v) \end{array}$$

(21) $$\begin{array}{rl} logit[Pr(Y_{iv} & =1|Z_{i},Y_{i(v-1)}=0,C_{iv}=0)]=\alpha Z_{i}+\gamma f(v)+\iota X_{i} \end{array}$$

(22) $$\begin{array}{rl} logit[Pr(Y_{iv} & =1|Z_{i},Y_{i(v-1)}=0,C_{iv}=0)]=\alpha Z_{i}+\gamma f(v)+\psi Z_{i}f(v)+\iota X_{i} \end{array}$$

where f(v)=v since we assumed a linear relationship and discrete time (visit).

#### 4.5.3. Summary of method application

The weighting process is implemented as described in Section 2.3.2 but with a different confounder set $K_{iv}$ chosen as specified in Table A5 (in Supplemental Material A). Then the outcome is modelled by (equations (19)21 22) using weighted population computed with weight=W (see equation (3)), weight=SW (see equation (5)) or $weight=SW_{B}$ (see equation (7)).

After fitting the outcome model, we obtain the marginal risk difference which is the difference between the cumulative incidence of each arm. For the outcome model containing baseline covariates, standardisation across these covariates is conducted.

#### 4.5.4. Methods under investigation

Table 3 summarises the factors that require specifications for IPCW implementations. To implement IPCW, there are in total four choices to be made to fully specify the model. Targeting the aim of exploring the precision of IPCW by different weighting models, we controlled the numerator by setting it to: unstabilised form, stabilised without baseline covariates form and stabilised with baseline form. Ideally, three choices should be pre-specified in a trial. The choices of the denominator and the correct specification of the outcome model mainly influence the accuracy of the estimates and we set them to particular levels that then remained unchanged in the main analysis. The choice of covariate adjustment influences the precision and to keep a single source of variation in standard error, we assumed that the covariate adjustment is done in all analyses. We also varied these three factors (which were designed to be outside our control range in this study) for sensitivity analyses.

### 4.6. Performance measures

Absolute bias, empirical standard error (EmpSE) and root mean squared error (RMSE) together with the corresponding Monte Carlo standard error (MCSE) for these performance measures are evaluated in the main analysis. We also report the model-based standard error (ModSE) calculated by bootstrap with 200 samples.

### 4.7. Implementation

We use the bootstrap method with 200 repetitions to obtain the ModSEs and to obtain a two-sided non-conservative 95% confidence interval accordingly for the cumulative incidence in all the scenarios.

The whole simulation was conducted using Stata software, version 16.0. Performance measures were computed by simsum.^34^ To visualise the comparison of performances between method implementations we used the siman package in Stata to produce nested-loop plots.^35^

## 5. Results

We investigated the factors listed in Table 2 and the properties of each scenario are provided in Tables B1 to B4 in Supplemental Material B. Table 3 summarises the factors varying IPCW implementations and Table A5 in Supplemental Material A lists details of every possible analysis within the study design. IPCW with three weighting choices (i.e. unstabilised, stabilised for time and stabilised for time and baseline) are taken to be within our control to explore the properties of stabilisation under two circumstances where the substantive outcome model is correctly specified or mis-specified. We also considered other choices including whether NUC is satisfied or not and whether covariate adjustment is planned for sensitivity analysis.

To validate the findings in the simulation, we performed multiple checks.^36^ We investigated the missing values and outliers in the datasets of estimates for each scenario. EmpSEs were compared with the ModSE provided by the bootstrapping method. From the results shown in Tables B5 to B8 in Supplemental Material B, we observe similar EmpSEs and ModSEs in each scenario for every method.

To provide a concise interpretation of the simulation results, we only present the results of IPCW implementations with NUC and with covariates adjusted in the outcome model among Scenarios 1–36 with a large magnitude of the indirect treatment effect by baseline covariates and a medium overall treatment effect. In terms of the patterns of the ICE, these scenarios cover situations where the ICE occur in one arm or both arms with medium prevalence ( 30%−40% ), high prevalence ( ≈60% ) or medium prevalence with deterministic occurrences. We expect and have observed similar properties in the rest of the scenarios. We provide nested-loop plots of the absolute bias, EmpSE and RMSE for the main analyses in this section (see Supplemental Material C to E for the rest of the analyses) and the summary of performance measures in Supplemental Tables B5 and B6. The magnitude of indirect effect by baseline covariates is explored by the concordance index shown in Supplemental Table A4. Box plots of the SD and max/min of the weights in control or both arms are also provided (see Supplemental Material F) for weight distributions.

### 5.1. Main analyses

**Figure 4.**
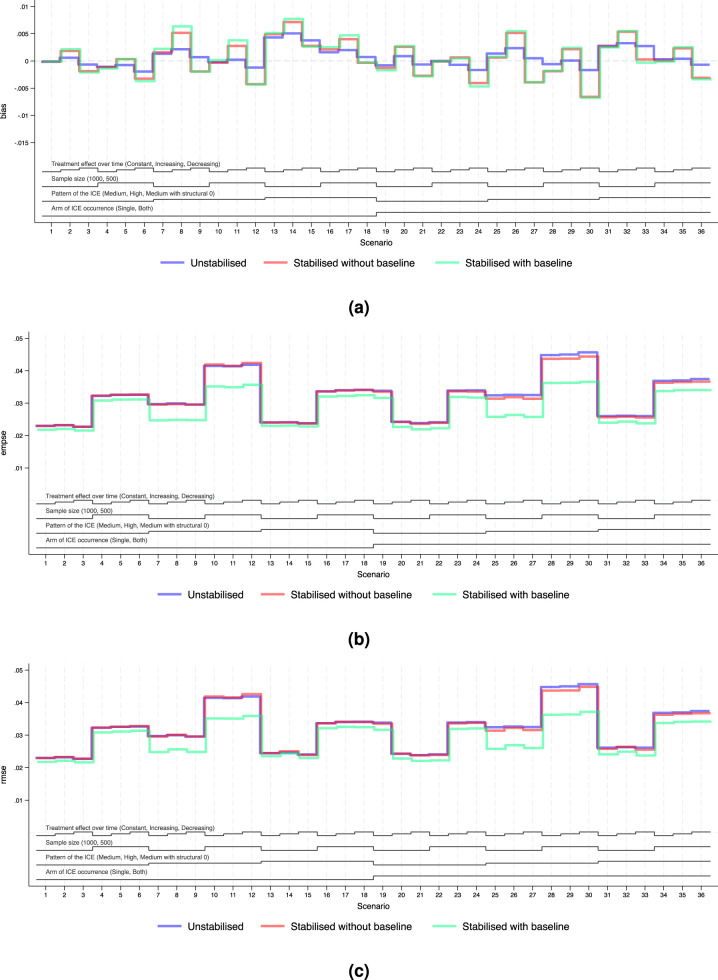
Performance measures of IPCW implementations in Scenarios 1 to 36 when outcome analysis model does not account for time-varying treatment effect and does covariate adjustment. *Note:* The black lines at the bottom indicate the factors varied in these scenarios as shown in Table 2. For each factor, the levels (as noted in brackets) are shown in the order that corresponds to the ascending steps of each line. (a) Performance measure: bias. Monte Carlo standard error ≤ 0.001, (b) performance measure: empirical standard error. Monte Carlo standard error ≤ 0.001 and (c) performance measure: root mean squared error. Monte Carlo standard error ≤ 0.001.

**Figure 5.**
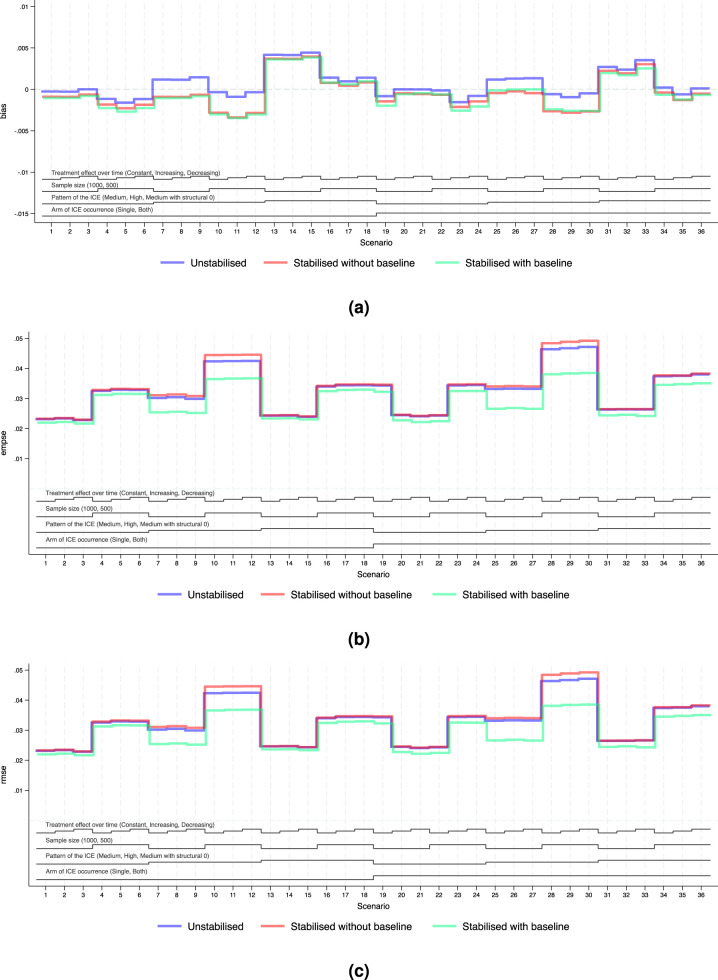
Performance measures of IPCW implementations in Scenarios 1 to 36 when outcome analysis model accounts for the time-varying treatment effect and does covariate adjustment. *Note:* The black lines at the bottom indicate the factors varied in these scenarios as shown in Table 2. For each factor, the levels (as noted in brackets) are shown in the order that corresponds to the ascending steps of each line. (a) Performance measure: bias. Monte Carlo standard error ≤ 0.002, (b) performance measure: empirical standard error. Monte Carlo standard error ≤ 0.001 and (c) performance measure: root mean squared error. Monte Carlo standard error ≤ 0.001.

For the main analyses, we consider the case where NUC is satisfied and covariate adjustment is conducted. Performance measures of bias, EmpSE and RMSE are visualised in Figures 4 and 5. Tables B5 and B6 in Supplemental Material B provide a full summary of performance measures.

#### 5.1.1. Distribution of weights

We explored the distribution of the weights by different forms of weighting models. For all forms of the weights, as the sample size gets smaller and as the prevalence of the ICE gets higher, the weights are more variable and the EmpSE is larger. Unstabilised weights are highly variable (see the figures in Supplemental Material F.2) and the EmpSE is large (see Figure 5(b)). We observed a reduction in max/min and standard deviation by stabilisation in the weights as shown in Supplemental Material F.2.

#### 5.1.2. Standard error

Figures 4(b) and 5(b) show the EmpSE of IPCW implementations with different forms of weights. As the sample size gets smaller, the prevalence of the ICE gets higher, the weights are more variable and the EmpSE is larger. IPCW stabilised for time (red lines) provides slightly reduced EmpSE compared with IPCW with unstabilised weight (blue lines) in most scenarios. An obvious reduction in EmpSE is observed in IPCW stabilising for time and baseline (green lines). We see a larger decrease of EmpSE when both arms have the ICE when the prevalence of ICE gets higher and when there is a structural violation of the positivity assumption. Among all 72 scenarios, the efficiency of the estimates was most obviously improved (by 20%) by stabilising for time and baseline in Scenarios 25–27 when ICE occurs in both arms, with a high prevalence of ICE and a sample size of 500. Practical random violation of the positivity assumption is more likely to occur leading to more variable weight estimation. In these situations, a bigger reduction in EmpSE when stabilisation is conducted for IPCW implementation is observed. From the summary statistics of the computed weights shown in Supplemental Figures F4 to F6, the max/min and SD of the weights is big and stabilisation reduces both of them substantially in the two situations mentioned above.

#### 5.1.3. Bias

Figures 4(a) and 5(a) show the bias of IPCW implementations with different forms of weights. The bias by three IPCW implementations is small when the positivity assumption is satisfied. In Scenarios 13–15 and 31–33 where the positivity assumption is violated, we see obvious bias which is over 2 times larger than the MCSE in all three IPCW implementations under investigation. Scenarios with time-varying treatment effect in the DGM in Figure 4 investigated situations where the outcome model is mis-specified failing to account for the treatment effect varying over time. In such cases, we see a larger bias in IPCW with stabilisation compared with IPCW with unstabilised weights.

#### 5.1.4. Root mean squared error

Figures 4(c) and 5(c) show the RMSEs of IPCW implementations with different forms of weights, which provide an overall summary of both bias and variability. The minor difference between EmpSE and RMSE suggests that variability dominates the total error, while bias remains relatively small across scenarios. When the outcome model is correctly specified, all methods present low RMSE and little difference between IPCW with stabilised and unstabilised weights. When the outcome model is mis-specified, we observe larger RMSE overall, but still relatively minor differences across stabilisation strategies.

Notably, under model mis-specification, IPCW with unstabilised weights yields slightly smaller RMSE compared to its stabilised counterparts and this is more evident in Scenarios 8, 9, 11 and 12 where the prevalence of the ICE is high. This can be attributed to the reintroduction of confounding through stabilisation, which introduces additional bias that can not be offset by gains in precision.

### 5.2. Sensitivity analyses

We assess the performance of varying factors which are outside the data analyst’s control (see Table 3) as sensitivity analyses.

#### 5.2.1. NUC assumption violation

The figures in Supplemental Material D show the results of IPCW implementations when the NUC assumption is violated by omitting a confounder. We draw identical conclusions as we do in situations where NUC is satisfied. Though there seems to be a smaller bias by IPCW with residual confounding compared with IPCW with NUC, this is probably because the bias induced by residual confounding and the bias caused by the mis-specification of the outcome model is in different directions and these biases cancel each other out.

#### 5.2.2. Without covariate adjustment

Figure C.2 in Supplemental Material C shows the performance of IPCW with different weighting models when there is no covariate adjustment. Findings in the case where covariate adjustment is conducted remain the same as when covariate adjustment is not done.

#### 5.2.3. Smaller magnitude of baseline covariate effect

In Supplemental Material E, we provided the simulation results for Scenarios 73–144 where the baseline covariates have a smaller effect on the potential outcome. Supplemental Table A4 shows the concordance index for different models aiming to compare the influence of the effect of baseline covariates. Compared with the results from Scenarios 1 to 72 where the baseline covariate effect is larger, we see a slightly reduced improvement of the precision which is thought to be negligible.

## 6. Discussion

Given the lack of consensus regarding when stabilisation is needed and whether it risks losing accuracy in the presence of model mis-specification, we used an illustrative example and Monte Carlo simulations to investigate the performance of potential IPCW implementations using different forms of the stabilised weight compared with using the unstabilised weight. In the simulation, the performance of all method implementations is investigated in a series of scenarios that were designed to represent real-world settings with the overall treatment effect designed to be small or moderate. The scenarios covered a broad range of situations where the key assumptions of the IPCW method are likely to be somehow violated. We varied the prevalence of the ICE in one or both arms ( 30% – 60% ) which was shown to be the most important factor affecting the performance of the IPCW method in previous studies.^11,12^ The cases where the positivity assumption is violated were realised by including the deterministic occurrence of the ICE in some scenarios. To compare the methods when the outcome model is violated, we designed the scenarios with different characteristics of treatment effect (time-invariant treatment effect, increasing treatment effect and decreasing treatment effect). While a sample size of 1000 is generally used in simulating a trial, a reduced size of 500 is also explored, accounting for both the potential small sample bias and the different behaviours of the IPCW implementations studied in EmpSE.

Some key findings addressed the aim of this study. In terms of efficiency, we showed that stabilising for time improved the precision of the estimates compared with the unstabilised counterpart when the substantive outcome model is not saturated. Stabilising for baseline covariates provided a substantial increase in the precision of the estimates. As the prevalence of the ICE became larger, the improvement in efficiency became more obvious. However, stabilisation could potentially result in extra bias when the outcome analysis model was mis-specified. Specifically, when the true treatment effect varied over time (increasing or decreasing) and a substantive outcome model wrongly assumed a constant treatment effect, implementing the IPCW estimator with stabilisation resulted in less accurate estimates compared with the unstabilised implementation.

Our findings have important practical implications for the use of IPCW in clinical trials, particularly in guiding the choice of stabilisation while considering model specification. In practice, IPCW often provides estimates with large variances, making improving precision an important objective. Meanwhile, achieving a correctly specified model is inherently challenging and rarely feasible. This highlights the trade-off between reducing variance through stabilisation and the potential for introducing bias when the model is misspecified. The key finding that stabilisation risks yielding additional bias when the treatment effect varies over time, is especially relevant given that the assumption of a constant treatment effect is often unrealistic. This issue is particularly critical in health technology assessment (HTA), where models that assume a constant treatment effect are often not used but the correct specification is hard to achieve.^37,38^

Beyond the specific issue of treatment effect misspecification over time investigated in this study, our findings also point to broader concerns regarding model misspecification. Stabilisation may amplify bias if the underlying model is misspecified, not only in terms of how the treatment effect evolves over time but also in relation to other covariates included in the weighting model. This highlights the importance of careful model specification and evaluations of underlying assumptions when implementing IPCW so that stabilisation is applied with enhanced precision without compromising validity. Given these risks, sensitivity analyses can be helpful in assessing the robustness of results under different model specifications, particularly in cases where there is uncertainty about the correct functional form of covariates or their interactions with treatment effects.

We acknowledge several limitations of this study and suggest directions for future research. First, the simulation study explored a limited range of scenarios, such as a modest overall treatment effect, a small magnitude of the time-varying treatment effect, and a strong indirect treatment effect mediated by baseline covariates. We believe these settings are realistic and likely to occur in practice, and the simulation results demonstrated clear properties and trends as the investigated factors were varied. Therefore, we expect the basic conclusions that are obtained in this simulation to remain unchanged for a broader range of situations. Second, this study assumes no selection bias by loss to follow-up. To address selection bias from loss to follow-up, general missing data methods can be applied before implementing IPCW. Treating loss to follow-up as another type of event, similar to censoring by ICEs, is expected to yield comparable conclusions. If weighting is also used to address censoring by loss to follow-up, stabilised weights are also recommended. The outcome model can then be applied after estimating probabilities for both ICEs and loss to follow-up. However, we have to note that loss to follow-up increases the risk of violating the positivity assumption, potentially leading to greater bias and reduced efficiency. Lastly, this study explores only one type of model misspecification: incorrectly assuming treatment effect over time is constant. There are other forms of incorrect specification of the outcome model that we have not covered. In terms of the baseline covariates, all implementations we explored correctly specified the terms concerning the covariates. The outcome model can be mis-specified such that the treatment effect varies with baseline covariates (e.g. age or baseline prognostic factors). Further studies can investigate cases where there is treatment effect heterogeneity (e.g. treatment interacts with a covariate). In such cases, the weights we obtained by the weighting model are specifically proportional to the covariate-specific information. When we ignore the interaction term in the outcome model, the estimates obtained are in fact a weighted average of the treatment effect in a subgroup of the total population with specific covariate values. We anticipate reaching a similar conclusion in that scenario, where stabilising by baseline covariates introduces extra bias.

In conclusion, our findings demonstrate that using stabilised weights in IPCW improves estimator efficiency, with a notable reduction in standard errors when baseline covariates are also stabilised. However, we also highlight the potential risks of stabilisation when the outcome model is mis-specified, which could lead to increased bias. The choice of weighting method should be empirically driven, based on the pre-specified outcome model and reasonable assumptions. In most cases, when we are confident in the specification of the outcome model, the efficiency gains from stabilisation outweigh the risk of bias. However, when there is uncertainty about the model specification, using unstabilised weights may be preferable to prioritise accuracy.

## Supplemental Material

sj-pdf-1-smm-10.1177_09622802251387456 - Supplemental material for Using inverse probability of censoring weighting to estimate hypothetical estimands in clinical trials: Should we implement stabilisation, and if so how? Supplemental material, sj-pdf-1-smm-10.1177_09622802251387456 for Using inverse probability of censoring weighting to estimate hypothetical estimands in clinical trials: Should we implement stabilisation, and if so how? by Jingyi Xuan, Shahrul Mt-Isa, Nicholas R Latimer, Helen Bell Gorrod, William Malbecq, Kristel Vandormael, Victoria Yorke-Edwards and Ian R White in Statistical Methods in Medical Research
