# Supplementary material for: Using inverse probability of censoring weighting to estimate hypothetical estimands in clinical trials: Should we implement stabilisation, and if so how?
Source: Stat Methods Med Res. 2025 Oct 31;35(1):40–60. doi: 10.1177/09622802251387456 (PMC12783383; doi:10.1177/09622802251387456)
Supplement: sj-pdf-1-smm-10.1177_09622802251387456 - Supplemental material for Using inverse probability of censoring weighting to estimate hypothetical estimands in clinical trials: Should we implement stabilisation, and if so how? [file sj-pdf-1-smm-10.1177_09622802251387456.pdf]

Using inverse probability of censoring weighting to estimate hypothetical estimands in clinical trials: Should we implement stabilisation, and if so how?

SUPPLEMENTARY MATERIALS

Appendix A: Details for simulation design

Appendix B: Tables with the summary of all scenarios

Appendix C: Nestedloop plots of performance measures for estimates by IPCW implementations with NUC

Appendix D: Nestedloop plots of performance measures for estimates by IPCW implementations with RC

Appendix E: Nestedloop plots of performance measures for scenarios 73-144 with smaller baseline effect

Appendix F: Summary of weights of IPCW

Appendix A Details for simulation design

A.1 Data-generating algorithm

For each individual $i = 1, \dots, n$:

1. At $v = 0$:
    - Generate values for all the covariates (time-independent and time-dependent) at $v = 0$: $L_{i01}, L_{i02}, L_{i03}, L_{i04}, L_{i05}$.
    - Generate treatment assignment by randomisation: Z_i
    - Set indicator of intervention deviation: $C_{0i} = 0$
    - Set time on and off treatment assigned by randomisation: $T_{i0on} = 0$; $T_{i0off} = 0$
    - Set indicator of outcome: $Y_{i0} = 0$
2. At $v = 1, 2, ..., 8$ (if $Y_{i(v-1)} = 0$):
    - Generate time-varying covariates: $L_{iv1}, L_{iv2}, L_{iv3}, L_{iv4}, L_{iv5}$
    - Update indicator of intervention deviation C_{iv}:
        - If $C_{i(v-1)} = 1$:
            * Set C_{iv} to NULL
        - If $C_{i(v-1)} = 0$:
            * Generate indicator of intervention deviation C_{iv} at visit v
    - Generate indicator of outcome: Y_{iv}
    - Update periods on and off treatment assigned: T_{ivon} and T_{ivoff}

A.2 List of scenarios designed

Table A1. List of scenarios 1-72 with varying factors described in Table 2. The base case is Scenario 1, with whom the other scenarios are to be compared by changing one factor at a time.

No.	Sample size	ICE		Treatment effect			No.	Sample size	ICE		Treatment effect		
		Arm	Pattern	Baseline*	Time-varying	Overall			Arm	Pattern	Baseline*	Time-varying	Overall
1	1000	Ctrl	medium	high	NA	medium	37	1000	Ctrl	medium	high	NA	low
2	1000	Ctrl	medium	high	Late	medium	38	1000	Ctrl	medium	high	Late	low
3	1000	Ctrl	medium	high	Early	medium	39	1000	Ctrl	medium	high	Early	low
4	500	Ctrl	medium	high	NA	medium	40	500	Ctrl	medium	high	NA	low
5	500	Ctrl	medium	high	Early	medium	41	500	Ctrl	medium	high	Early	low
6	500	Ctrl	medium	high	Late	medium	42	500	Ctrl	medium	high	Late	low
7	1000	Ctrl	high	high	NA	medium	43	1000	Ctrl	high	high	NA	low
8	1000	Ctrl	high	high	Early	medium	44	1000	Ctrl	high	high	Early	low
9	1000	Ctrl	high	high	Late	medium	45	1000	Ctrl	high	high	Late	low
10	500	Ctrl	high	high	NA	medium	46	500	Ctrl	high	high	NA	low
11	500	Ctrl	high	high	Early	medium	47	500	Ctrl	high	high	Early	low
12	500	Ctrl	high	high	Late	medium	48	500	Ctrl	high	high	Late	low
13	1000	Ctrl	Deterministic	high	NA	medium	49	1000	Ctrl	Deterministic	high	NA	low
14	1000	Ctrl	Deterministic	high	Early	medium	50	1000	Ctrl	Deterministic	high	Early	low
15	1000	Ctrl	Deterministic	high	Late	medium	51	1000	Ctrl	Deterministic	high	Late	low
16	500	Ctrl	Deterministic	high	NA	medium	52	500	Ctrl	Deterministic	high	NA	low
17	500	Ctrl	Deterministic	high	Early	medium	53	500	Ctrl	Deterministic	high	Early	low
18	500	Ctrl	Deterministic	high	Late	medium	54	500	Ctrl	Deterministic	high	Late	low
19	1000	Both	medium	high	NA	medium	55	1000	Both	medium	high	NA	low
20	1000	Both	medium	high	Late	medium	56	1000	Both	medium	high	Late	low
21	1000	Both	medium	high	Early	medium	57	1000	Both	medium	high	Early	low
22	500	Both	medium	high	NA	medium	58	500	Both	medium	high	NA	low
23	500	Both	medium	high	Early	medium	59	500	Both	medium	high	Early	low
24	500	Both	medium	high	Late	medium	60	500	Both	medium	high	Late	low
25	1000	Both	high	high	NA	medium	61	1000	Both	high	high	NA	low
26	1000	Both	high	high	Early	medium	62	1000	Both	high	high	Early	low
27	1000	Both	high	high	Late	medium	63	1000	Both	high	high	Late	low
28	500	Both	high	high	NA	medium	64	500	Both	high	high	NA	low
29	500	Both	high	high	Early	medium	65	500	Both	high	high	Early	low
30	500	Both	high	high	Late	medium	66	500	Both	high	high	Late	low
31	1000	Both	Deterministic	high	NA	medium	67	1000	Both	Deterministic	high	NA	low
32	1000	Both	Deterministic	high	Early	medium	68	1000	Both	Deterministic	high	Early	low
33	1000	Both	Deterministic	high	Late	medium	69	1000	Both	Deterministic	high	Late	low
34	500	Both	Deterministic	high	NA	medium	70	500	Both	Deterministic	high	NA	low
35	500	Both	Deterministic	high	Early	medium	71	500	Both	Deterministic	high	Early	low
36	500	Both	Deterministic	high	Late	medium	72	500	Both	Deterministic	high	Late	low

Note: ICE = Intercurrent events; Ctrl = Control; TE = Treatment effect.
* Magnitude of indirect treatment effect by baseline covariates.

Table A2. List of scenarios 73-144 with varying factors described in Table 2. The base case is Scenario 1, with whom the other scenarios are to be compared by changing one factor at a time.

No.	Sample size	ICE		Treatment effect			No.	Sample size	ICE		Treatment effect		
		Arm	Pattern	Baseline*	Time-varying	Overall			Arm	Pattern	Baseline*	Time-varying	Overall
73	1000	Ctrl	medium	medium	NA	medium	109	1000	Ctrl	medium	medium	NA	low
74	1000	Ctrl	medium	medium	Late	medium	110	1000	Ctrl	medium	medium	Late	low
75	1000	Ctrl	medium	medium	Early	medium	111	1000	Ctrl	medium	medium	Early	low
76	500	Ctrl	medium	medium	NA	medium	112	500	Ctrl	medium	medium	NA	low
77	500	Ctrl	medium	medium	Early	medium	113	500	Ctrl	medium	medium	Early	low
78	500	Ctrl	medium	medium	Late	medium	114	500	Ctrl	medium	medium	Late	low
79	1000	Ctrl	high	medium	NA	medium	115	1000	Ctrl	high	medium	NA	low
80	1000	Ctrl	high	medium	Early	medium	116	1000	Ctrl	high	medium	Early	low
81	1000	Ctrl	high	medium	Late	medium	117	1000	Ctrl	high	medium	Late	low
82	500	Ctrl	high	medium	NA	medium	118	500	Ctrl	high	medium	NA	low
83	500	Ctrl	high	medium	Early	medium	119	500	Ctrl	high	medium	Early	low
84	500	Ctrl	high	medium	Late	medium	120	500	Ctrl	high	medium	Late	low
85	1000	Ctrl	Deterministic	medium	NA	medium	121	1000	Ctrl	Deterministic	medium	NA	low
86	1000	Ctrl	Deterministic	medium	Early	medium	122	1000	Ctrl	Deterministic	medium	Early	low
87	1000	Ctrl	Deterministic	medium	Late	medium	123	1000	Ctrl	Deterministic	medium	Late	low
88	500	Ctrl	Deterministic	medium	NA	medium	124	500	Ctrl	Deterministic	medium	NA	low
89	500	Ctrl	Deterministic	medium	Early	medium	125	500	Ctrl	Deterministic	medium	Early	low
90	500	Ctrl	Deterministic	medium	Late	medium	126	500	Ctrl	Deterministic	medium	Late	low
91	1000	Both	medium	medium	NA	medium	127	1000	Both	medium	medium	NA	low
92	1000	Both	medium	medium	Late	medium	128	1000	Both	medium	medium	Late	low
93	1000	Both	medium	medium	Early	medium	129	1000	Both	medium	medium	Early	low
94	500	Both	medium	medium	NA	medium	130	500	Both	medium	medium	NA	low
95	500	Both	medium	medium	Early	medium	131	500	Both	medium	medium	Early	low
96	500	Both	medium	medium	Late	medium	132	500	Both	medium	medium	Late	low
97	1000	Both	high	medium	NA	medium	133	1000	Both	high	medium	NA	low
98	1000	Both	high	medium	Early	medium	134	1000	Both	high	medium	Early	low
99	1000	Both	high	medium	Late	medium	135	1000	Both	high	medium	Late	low
100	500	Both	high	medium	NA	medium	136	500	Both	high	medium	NA	low
101	500	Both	high	medium	Early	medium	137	500	Both	high	medium	Early	low
102	500	Both	high	medium	Late	medium	138	500	Both	high	medium	Late	low
103	1000	Both	Deterministic	medium	NA	medium	139	1000	Both	Deterministic	medium	NA	low
104	1000	Both	Deterministic	medium	Early	medium	140	1000	Both	Deterministic	medium	Early	low
105	1000	Both	Deterministic	medium	Late	medium	141	1000	Both	Deterministic	medium	Late	low
106	500	Both	Deterministic	medium	NA	medium	142	500	Both	Deterministic	medium	NA	low
107	500	Both	Deterministic	medium	Early	medium	143	500	Both	Deterministic	medium	Early	low
108	500	Both	Deterministic	medium	Late	medium	144	500	Both	Deterministic	medium	Late	low

Note: ICE = Intercurrent events; Ctrl = Control; TE = Treatment effect.
* Magnitude of indirect treatment effect by baseline covariates.

A.3 Numerical values for simulation models

$$\boldsymbol{\mu_0} = (11.95, 1.97, 5.79, -1.10, 4.39, 95.77, 138.13) \quad (1)$$

$$\boldsymbol{m} = (2.91, 1.00, 0.69, -5.07, 1.30, 1.26, 31.71) \quad (2)$$

$$\boldsymbol{n} = (17.99, 4.00, 8.00, 2.67, 6.64, 374.18, 256.00) \quad (3)$$

$$\boldsymbol{\Sigma} = \begin{pmatrix} 1 & 0.04 & -0.27 & -0.20 & -0.05 & 0.01 & 0.10 \\ 0.04 & 1 & -0.30 & -0.30 & 0.17 & 0.03 & -0.03 \\ -0.27 & -0.30 & 1 & 0.49 & -0.42 & -0.13 & 0.07 \\ -0.20 & -0.30 & 0.49 & 1 & -0.46 & -0.14 & 0.09 \\ -0.05 & 0.17 & -0.42 & -0.46 & 1 & 0.14 & -0.14 \\ 0.01 & 0.03 & -0.13 & -0.14 & 0.14 & 1 & 0.10 \\ 0.10 & -0.03 & 0.07 & 0.09 & -0.14 & 0.10 & 1 \end{pmatrix} \quad (4)$$

$$\boldsymbol{\rho} = (\rho_0, \rho_{31}, \rho_{32}, \rho_{34}) = (-3.00, 0.46, -0.83, -0.01) \tag{5}$$

$$\boldsymbol{\Lambda} = \begin{pmatrix} 1 & 0.40 \\ 0.40 & 1 \end{pmatrix} \tag{6}$$

Table A3. Summary of numerical values for parameters in TVC model

TVC	β_{q0}	β_{q1}	β_{q2}	β_{q3}	$\boldsymbol{\beta_{q4}}$	β_{q5}	sd_q
L_1	2.39	0.20	*	*	(-0.02,-0.04, -0.01)	0.66	0.70
L_2	0.11	0.10	*	*	(-0.01,-0.07, -0.02)	0.72	0.78
L_3	1.29	-0.17	0.24	0	(0,0,0.05)	0.25	0.81
L_4	67.47	0	7.89	0	(0,-2.99,0)	0	0.57
L_5	84.00	-13.97	0	0	(-0.92,0,-3.78)	0.42	53.55

* Values for β_{12}, β_{13}, β_{22} and β_{23} vary

$$\boldsymbol{\theta_3} = (0.24, 0.50, -0.83) \tag{7}$$

$$\mu_2 = -0.13 \tag{8}$$

$$\boldsymbol{\mu_6} = (0.04, 0.18, 0) \tag{9}$$

A.4 Concordance index

Table A4. Concordance index for different models

	$Y\|X, v = 8$	$Y\|X, v$	$Y\|v$
S1-72	0.78	0.65	0.60
S73-144	0.81	0.64	0.56

Note: The statistics are means of the c-index in small number of simulations with 100 replications. Scenarios 1-72 have large indirect effect of baseline covariates while Scenarios 73-144 have smaller effect.

A.5 Description of all IPCW implementations investigated

Table A5. List of IPCW implementations under investigation

No.	Numerator in weighting model	Denominator in weighting model	Outcome model ‡
IPCW without stabilisation, NUC			
1	1	v, $\boldsymbol{X}$, L_1, L_2	without time*trt
2	1	v, $\boldsymbol{X}$, L_1, L_2	with time*trt
IPCW with stabilisation, NUC			
	Time only		
3	v	v, $\boldsymbol{X}$, L_1, L_2	without time*trt
4	v	v, $\boldsymbol{X}$, L_1, L_2	with time*trt
	Time and baseline covariates		
5	v, $\boldsymbol{X}$, L_{01}, L_{02}	v, $\boldsymbol{X}$, L_1, L_2	without time*trt
6	v, $\boldsymbol{X}$, L_{01}, L_{02}	v, $\boldsymbol{X}$, L_1, L_2	with time*trt
*IPCW without stabilisation, RC**			
7	1	v, $\boldsymbol{X}$, L_2	without time*trt
8	1	v, $\boldsymbol{X}$, L_2	with time*trt
IPCW with stabilisation, RC			
	Time only		
9	v	v, $\boldsymbol{X}$, L_2	without time*trt
10	v	v, $\boldsymbol{X}$, L_2	with time*trt
	Time and baseline covariates		
11	v, $\boldsymbol{X}$, L_{02} †	v, $\boldsymbol{X}$, L_2	without time*trt
12	v, $\boldsymbol{X}$, L_{02} †	v, $\boldsymbol{X}$, L_2	with time*trt

Note: NUC = No unmeasured confounders; RC = Residual confounding; IPCW implementations vary in the weighting model considering the numerator (unstabilised/stabilised) and the denominator (NUC/RC) of the weighting model and in the outcome analysis model (accounting for time-varying treatment or not).

* RC is realised by omitting confounder L_1.

†When there is residual confounding caused by unmeasured confounder L_1, the numerator attempting to adjust for baseline covariates will only include baseline measurement of L_2 (L_{02}).

‡Outcome analysis model not accounting for the time-varying treatment effect is mis-specified if the actual treatment effect specified in the data-generating mechanism is time-varying.

Appendix B Tables with summary of all scenarios

B.1 Tables summarising the characteristics of the simulated scenarios

Table B1. Table for a summary of simulated data from Scenarios 1 to 36.

	ICE in Ctrl arm		ICE in Exp arm		Failure in Ctrl arm		Failure in Exp arm		True risk difference	
	Mean	SD	Mean	SD	Mean	SD	Mean	SD	Mean	SD
1	0.34	0.02	0.00	0.00	0.16	0.02	0.07	0.01	-0.0899	0.0006
2	0.34	0.02	0.00	0.00	0.16	0.02	0.08	0.01	-0.0982	0.0006
3	0.34	0.02	0.00	0.00	0.16	0.02	0.07	0.01	-0.0955	0.0006
4	0.34	0.03	0.00	0.00	0.16	0.02	0.07	0.02	-0.0899	0.0006
5	0.34	0.03	0.00	0.00	0.16	0.02	0.08	0.02	-0.0982	0.0006
6	0.34	0.03	0.00	0.00	0.16	0.02	0.07	0.02	-0.0955	0.0006
7	0.56	0.02	0.00	0.00	0.16	0.02	0.07	0.01	-0.0899	0.0006
8	0.56	0.02	0.00	0.00	0.16	0.02	0.08	0.01	-0.0982	0.0006
9	0.56	0.02	0.00	0.00	0.16	0.02	0.07	0.01	-0.0955	0.0006
10	0.56	0.03	0.00	0.00	0.16	0.02	0.07	0.02	-0.0899	0.0006
11	0.56	0.03	0.00	0.00	0.16	0.02	0.08	0.02	-0.0982	0.0006
12	0.56	0.03	0.00	0.00	0.16	0.02	0.07	0.02	-0.0955	0.0006
13	0.39	0.02	0.00	0.00	0.16	0.02	0.07	0.01	-0.0899	0.0006
14	0.39	0.02	0.00	0.00	0.16	0.02	0.08	0.01	-0.0982	0.0006
15	0.39	0.02	0.00	0.00	0.16	0.02	0.07	0.01	-0.0955	0.0006
16	0.39	0.03	0.00	0.00	0.16	0.02	0.07	0.02	-0.0899	0.0006
17	0.39	0.03	0.00	0.00	0.16	0.02	0.08	0.02	-0.0982	0.0006
18	0.39	0.03	0.00	0.00	0.16	0.02	0.07	0.02	-0.0955	0.0006
19	0.34	0.02	0.32	0.02	0.16	0.02	0.07	0.01	-0.0899	0.0006
20	0.34	0.02	0.32	0.02	0.16	0.02	0.08	0.01	-0.0982	0.0006
21	0.34	0.02	0.32	0.02	0.16	0.02	0.06	0.01	-0.0955	0.0006
22	0.34	0.03	0.32	0.03	0.16	0.02	0.07	0.02	-0.0899	0.0006
23	0.34	0.03	0.32	0.03	0.16	0.02	0.08	0.02	-0.0982	0.0006
24	0.34	0.03	0.32	0.03	0.16	0.02	0.06	0.01	-0.0955	0.0006
25	0.56	0.02	0.54	0.02	0.16	0.02	0.07	0.01	-0.0899	0.0006
26	0.56	0.02	0.54	0.02	0.16	0.02	0.08	0.01	-0.0982	0.0006
27	0.56	0.02	0.54	0.02	0.16	0.02	0.06	0.01	-0.0955	0.0006
28	0.56	0.03	0.54	0.03	0.16	0.02	0.07	0.02	-0.0899	0.0006
29	0.56	0.03	0.54	0.03	0.16	0.02	0.08	0.02	-0.0982	0.0006
30	0.56	0.03	0.54	0.03	0.16	0.02	0.06	0.01	-0.0955	0.0006
31	0.39	0.02	0.37	0.02	0.16	0.02	0.07	0.01	-0.0899	0.0006
32	0.39	0.02	0.37	0.02	0.16	0.02	0.08	0.01	-0.0982	0.0006
33	0.39	0.02	0.37	0.02	0.16	0.02	0.06	0.01	-0.0955	0.0006
34	0.39	0.03	0.37	0.03	0.16	0.02	0.07	0.02	-0.0899	0.0006
35	0.39	0.03	0.37	0.03	0.16	0.02	0.08	0.02	-0.0982	0.0006
36	0.39	0.03	0.37	0.03	0.16	0.02	0.06	0.02	-0.0955	0.0006

Table B2. Table for a summary of simulated data in Scenarios 37 to 72.

	ICE in Ctrl arm		ICE in Exp arm		Failure in Ctrl arm		Failure in Exp arm		True risk difference	
	Mean	SD	Mean	SD	Mean	SD	Mean	SD	Mean	SD
37	0.34	0.02	0.00	0.00	0.16	0.02	0.10	0.01	-0.0638	0.0007
38	0.34	0.02	0.00	0.00	0.16	0.02	0.11	0.01	-0.0559	0.0007
39	0.34	0.02	0.00	0.00	0.16	0.02	0.10	0.01	-0.0674	0.0007
40	0.34	0.03	0.00	0.00	0.16	0.02	0.10	0.02	-0.0638	0.0007
41	0.34	0.03	0.00	0.00	0.16	0.02	0.11	0.02	-0.0559	0.0007
42	0.34	0.03	0.00	0.00	0.16	0.02	0.10	0.02	-0.0674	0.0007
43	0.56	0.02	0.00	0.00	0.16	0.02	0.10	0.01	-0.0638	0.0007
44	0.56	0.02	0.00	0.00	0.16	0.02	0.11	0.01	-0.0559	0.0007
45	0.56	0.02	0.00	0.00	0.16	0.02	0.10	0.01	-0.0674	0.0007
46	0.56	0.03	0.00	0.00	0.16	0.02	0.10	0.02	-0.0638	0.0007
47	0.56	0.03	0.00	0.00	0.16	0.02	0.11	0.02	-0.0559	0.0007
48	0.56	0.03	0.00	0.00	0.16	0.02	0.10	0.02	-0.0674	0.0007
49	0.39	0.02	0.00	0.00	0.16	0.02	0.10	0.01	-0.0638	0.0007
50	0.39	0.02	0.00	0.00	0.16	0.02	0.11	0.01	-0.0559	0.0007
51	0.39	0.02	0.00	0.00	0.16	0.02	0.10	0.01	-0.0674	0.0007
52	0.39	0.03	0.00	0.00	0.16	0.02	0.10	0.02	-0.0638	0.0007
53	0.39	0.03	0.00	0.00	0.16	0.02	0.11	0.02	-0.0559	0.0007
54	0.39	0.03	0.00	0.00	0.16	0.02	0.10	0.02	-0.0674	0.0007
55	0.34	0.02	0.32	0.02	0.16	0.02	0.10	0.01	-0.0638	0.0007
56	0.34	0.02	0.32	0.02	0.16	0.02	0.11	0.01	-0.0559	0.0007
57	0.34	0.02	0.32	0.02	0.16	0.02	0.09	0.01	-0.0674	0.0007
58	0.34	0.03	0.32	0.03	0.16	0.02	0.10	0.02	-0.0638	0.0007
59	0.34	0.03	0.32	0.03	0.16	0.02	0.11	0.02	-0.0559	0.0007
60	0.34	0.03	0.32	0.03	0.16	0.02	0.09	0.02	-0.0674	0.0007
61	0.56	0.02	0.54	0.02	0.16	0.02	0.10	0.01	-0.0638	0.0007
62	0.56	0.02	0.54	0.02	0.16	0.02	0.11	0.01	-0.0559	0.0007
63	0.56	0.02	0.54	0.02	0.16	0.02	0.09	0.01	-0.0674	0.0007
64	0.56	0.03	0.54	0.03	0.16	0.02	0.10	0.02	-0.0638	0.0007
65	0.56	0.03	0.54	0.03	0.16	0.02	0.11	0.02	-0.0559	0.0007
66	0.56	0.03	0.54	0.03	0.16	0.02	0.09	0.02	-0.0674	0.0007
67	0.39	0.02	0.37	0.02	0.16	0.02	0.10	0.01	-0.0638	0.0007
68	0.39	0.02	0.37	0.02	0.16	0.02	0.11	0.01	-0.0559	0.0007
69	0.39	0.02	0.37	0.02	0.16	0.02	0.09	0.01	-0.0674	0.0007
70	0.39	0.03	0.37	0.03	0.16	0.02	0.10	0.02	-0.0638	0.0007
71	0.39	0.03	0.37	0.03	0.16	0.02	0.11	0.02	-0.0559	0.0007
72	0.39	0.03	0.37	0.03	0.16	0.02	0.09	0.02	-0.0674	0.0007

Table B3. Table for a summary of simulated data from Scenarios 73 to 108.

	ICE in Ctrl arm		ICE in Exp arm		Failure in Ctrl arm		Failure in Exp arm		True risk difference	
	Mean	SD	Mean	SD	Mean	SD	Mean	SD	Mean	SD
73	0.34	0.02	0.00	0.00	0.13	0.01	0.06	0.01	-0.0719	0.0006
74	0.34	0.02	0.00	0.00	0.13	0.01	0.06	0.01	-0.0786	0.0005
75	0.34	0.02	0.00	0.00	0.13	0.01	0.05	0.01	-0.0765	0.0005
76	0.34	0.03	0.00	0.00	0.13	0.02	0.06	0.01	-0.0719	0.0006
77	0.34	0.03	0.00	0.00	0.13	0.02	0.06	0.01	-0.0786	0.0005
78	0.34	0.03	0.00	0.00	0.13	0.02	0.05	0.01	-0.0765	0.0005
79	0.56	0.02	0.00	0.00	0.12	0.01	0.06	0.01	-0.0719	0.0006
80	0.56	0.02	0.00	0.00	0.12	0.01	0.06	0.01	-0.0786	0.0005
81	0.56	0.02	0.00	0.00	0.12	0.01	0.05	0.01	-0.0765	0.0005
82	0.56	0.03	0.00	0.00	0.13	0.02	0.06	0.01	-0.0719	0.0006
83	0.56	0.03	0.00	0.00	0.13	0.02	0.06	0.01	-0.0786	0.0005
84	0.56	0.03	0.00	0.00	0.13	0.02	0.05	0.01	-0.0765	0.0005
85	0.39	0.02	0.00	0.00	0.13	0.01	0.06	0.01	-0.0719	0.0006
86	0.39	0.02	0.00	0.00	0.13	0.01	0.06	0.01	-0.0786	0.0005
87	0.39	0.02	0.00	0.00	0.13	0.01	0.05	0.01	-0.0765	0.0005
88	0.39	0.03	0.00	0.00	0.13	0.02	0.06	0.01	-0.0719	0.0006
89	0.39	0.03	0.00	0.00	0.13	0.02	0.06	0.01	-0.0786	0.0005
90	0.39	0.03	0.00	0.00	0.13	0.02	0.05	0.01	-0.0765	0.0005
91	0.34	0.02	0.32	0.02	0.13	0.01	0.05	0.01	-0.0719	0.0006
92	0.34	0.02	0.32	0.02	0.13	0.01	0.06	0.01	-0.0786	0.0005
93	0.34	0.02	0.32	0.02	0.13	0.01	0.05	0.01	-0.0765	0.0005
94	0.34	0.03	0.32	0.03	0.13	0.02	0.05	0.01	-0.0719	0.0006
95	0.34	0.03	0.32	0.03	0.13	0.02	0.06	0.01	-0.0786	0.0005
96	0.34	0.03	0.32	0.03	0.13	0.02	0.05	0.01	-0.0765	0.0005
97	0.56	0.02	0.54	0.02	0.12	0.01	0.05	0.01	-0.0719	0.0006
98	0.56	0.02	0.54	0.02	0.12	0.01	0.06	0.01	-0.0786	0.0005
99	0.56	0.02	0.54	0.02	0.12	0.01	0.05	0.01	-0.0765	0.0005
100	0.56	0.03	0.54	0.03	0.13	0.02	0.05	0.01	-0.0719	0.0006
101	0.56	0.03	0.54	0.03	0.13	0.02	0.06	0.01	-0.0786	0.0005
102	0.56	0.03	0.54	0.03	0.13	0.02	0.05	0.01	-0.0765	0.0005
103	0.39	0.02	0.37	0.02	0.13	0.01	0.05	0.01	-0.0719	0.0006
104	0.39	0.02	0.37	0.02	0.13	0.01	0.06	0.01	-0.0786	0.0005
105	0.39	0.02	0.37	0.02	0.13	0.01	0.05	0.01	-0.0765	0.0005
106	0.39	0.03	0.37	0.03	0.13	0.02	0.05	0.01	-0.0719	0.0006
107	0.39	0.03	0.37	0.03	0.13	0.02	0.06	0.01	-0.0786	0.0005
108	0.39	0.03	0.37	0.03	0.13	0.02	0.05	0.01	-0.0765	0.0005

Table B4. Table for a summary of simulated data in Scenarios 109 to 144.

	ICE in Ctrl arm		ICE in Exp arm		Failure in Ctrl arm		Failure in Exp arm		True risk difference	
	Mean	SD	Mean	SD	Mean	SD	Mean	SD	Mean	SD
109	0.34	0.02	0.00	0.00	0.13	0.01	0.08	0.01	-0.0514	0.0007
110	0.34	0.02	0.00	0.00	0.13	0.01	0.09	0.01	-0.0451	0.0006
111	0.34	0.02	0.00	0.00	0.13	0.01	0.08	0.01	-0.0543	0.0006
112	0.34	0.03	0.00	0.00	0.13	0.02	0.08	0.02	-0.0514	0.0007
113	0.34	0.03	0.00	0.00	0.13	0.02	0.09	0.02	-0.0451	0.0006
114	0.34	0.03	0.00	0.00	0.13	0.02	0.08	0.02	-0.0543	0.0006
115	0.56	0.02	0.00	0.00	0.12	0.01	0.08	0.01	-0.0514	0.0007
116	0.56	0.02	0.00	0.00	0.12	0.01	0.09	0.01	-0.0451	0.0006
117	0.56	0.02	0.00	0.00	0.12	0.01	0.08	0.01	-0.0543	0.0006
118	0.56	0.03	0.00	0.00	0.13	0.02	0.08	0.02	-0.0514	0.0007
119	0.56	0.03	0.00	0.00	0.13	0.02	0.09	0.02	-0.0451	0.0006
120	0.56	0.03	0.00	0.00	0.13	0.02	0.08	0.02	-0.0543	0.0006
121	0.39	0.02	0.00	0.00	0.13	0.01	0.08	0.01	-0.0514	0.0007
122	0.39	0.02	0.00	0.00	0.13	0.01	0.09	0.01	-0.0451	0.0006
123	0.39	0.02	0.00	0.00	0.13	0.01	0.08	0.01	-0.0543	0.0006
124	0.39	0.03	0.00	0.00	0.13	0.02	0.08	0.02	-0.0514	0.0007
125	0.39	0.03	0.00	0.00	0.13	0.02	0.09	0.02	-0.0451	0.0006
126	0.39	0.03	0.00	0.00	0.13	0.02	0.08	0.02	-0.0543	0.0006
127	0.34	0.02	0.32	0.02	0.13	0.01	0.08	0.01	-0.0514	0.0007
128	0.34	0.02	0.32	0.02	0.13	0.01	0.09	0.01	-0.0451	0.0006
129	0.34	0.02	0.32	0.02	0.13	0.01	0.07	0.01	-0.0543	0.0006
130	0.34	0.03	0.32	0.03	0.13	0.02	0.08	0.02	-0.0514	0.0007
131	0.34	0.03	0.32	0.03	0.13	0.02	0.09	0.02	-0.0451	0.0006
132	0.34	0.03	0.32	0.03	0.13	0.02	0.07	0.02	-0.0543	0.0006
133	0.56	0.02	0.54	0.02	0.12	0.01	0.07	0.01	-0.0514	0.0007
134	0.56	0.02	0.54	0.02	0.12	0.01	0.09	0.01	-0.0451	0.0006
135	0.56	0.02	0.54	0.02	0.12	0.01	0.07	0.01	-0.0543	0.0006
136	0.56	0.03	0.54	0.03	0.13	0.02	0.07	0.02	-0.0514	0.0007
137	0.56	0.03	0.54	0.03	0.13	0.02	0.08	0.02	-0.0451	0.0006
138	0.56	0.03	0.54	0.03	0.13	0.02	0.07	0.02	-0.0543	0.0006
139	0.39	0.02	0.37	0.02	0.13	0.01	0.08	0.01	-0.0514	0.0007
140	0.39	0.02	0.37	0.02	0.13	0.01	0.09	0.01	-0.0451	0.0006
141	0.39	0.02	0.37	0.02	0.13	0.01	0.07	0.01	-0.0543	0.0006
142	0.39	0.03	0.37	0.03	0.13	0.02	0.08	0.02	-0.0514	0.0007
143	0.39	0.03	0.37	0.03	0.13	0.02	0.09	0.02	-0.0451	0.0006
144	0.39	0.03	0.37	0.03	0.13	0.02	0.07	0.02	-0.0543	0.0006

B.2 Tables summarising the performance measures

Table B5. Table for estimates by IPCW when the outcome analysis model does not account for time-varying treatment effect and does covariate adjustment.

	IPCWu_NUC			IPCWs_t_NUC			IPCWs_tL_NUC			IPCWu_RC			IPCWs_t_RC			IPCWs_tL_RC			Max_MCSE		
	Estimate	EmpSE	ModSE	Estimate	EmpSE	ModSE	Estimate	EmpSE	ModSE	Estimate	EmpSE	ModSE	Estimate	EmpSE	ModSE	Estimate	EmpSE	ModSE	Mean	EmpSE	ModSE
1	-0.0947	0.0224	0.0227	-0.0947	0.0224	0.0227	-0.0946	0.0214	0.0220	-0.0933	0.0221	0.0225	-0.0933	0.0221	0.0225	-0.0934	0.0212	0.0219	0.0007	0.0005	0.0001
2	-0.0883	0.0225	0.0228	-0.0871	0.0225	0.0229	-0.0868	0.0214	0.0222	-0.0870	0.0222	0.0227	-0.0858	0.0222	0.0227	-0.0856	0.0212	0.0220	0.0007	0.0005	0.0001
3	-0.0978	0.0222	0.0226	-0.0991	0.0222	0.0226	-0.0993	0.0212	0.0220	-0.0964	0.0220	0.0224	-0.0977	0.0219	0.0224	-0.0981	0.0210	0.0218	0.0007	0.0005	0.0001
4	-0.0947	0.0317	0.0320	-0.0947	0.0317	0.0322	-0.0949	0.0305	0.0314	-0.0934	0.0314	0.0318	-0.0934	0.0314	0.0319	-0.0937	0.0303	0.0311	0.0010	0.0007	0.0001
5	-0.0882	0.0325	0.0323	-0.0871	0.0325	0.0324	-0.0870	0.0314	0.0316	-0.0869	0.0322	0.0320	-0.0858	0.0321	0.0321	-0.0858	0.0311	0.0314	0.0010	0.0007	0.0001
6	-0.0981	0.0316	0.0319	-0.0994	0.0315	0.0321	-0.0997	0.0303	0.0313	-0.0967	0.0312	0.0317	-0.0980	0.0312	0.0318	-0.0985	0.0300	0.0310	0.0010	0.0007	0.0001
7	-0.0940	0.0288	0.0275	-0.0938	0.0288	0.0276	-0.0937	0.0242	0.0248	-0.0913	0.0284	0.0271	-0.0911	0.0282	0.0271	-0.0914	0.0237	0.0244	0.0009	0.0007	0.0002
8	-0.0876	0.0292	0.0277	-0.0847	0.0293	0.0277	-0.0839	0.0239	0.0247	-0.0850	0.0287	0.0273	-0.0820	0.0286	0.0273	-0.0815	0.0235	0.0243	0.0009	0.0007	0.0003
9	-0.0973	0.0289	0.0275	-0.1000	0.0289	0.0277	-0.1006	0.0244	0.0250	-0.0946	0.0285	0.0271	-0.0973	0.0284	0.0272	-0.0981	0.0239	0.0245	0.0009	0.0007	0.0002
10	-0.0939	0.0412	0.0382	-0.0939	0.0411	0.0385	-0.0931	0.0353	0.0353	-0.0915	0.0406	0.0379	-0.0915	0.0407	0.0382	-0.0910	0.0347	0.0346	0.0013	0.0009	0.0003
11	-0.0873	0.0420	0.0383	-0.0845	0.0418	0.0385	-0.0833	0.0358	0.0352	-0.0850	0.0414	0.0381	-0.0822	0.0413	0.0382	-0.0812	0.0352	0.0346	0.0013	0.0009	0.0003
12	-0.0974	0.0411	0.0382	-0.1003	0.0410	0.0387	-0.1001	0.0353	0.0355	-0.0950	0.0405	0.0380	-0.0979	0.0406	0.0384	-0.0979	0.0346	0.0349	0.0013	0.0009	0.0003
13	-0.0923	0.0234	0.0236	-0.0918	0.0233	0.0236	-0.0914	0.0222	0.0228	-0.0903	0.0232	0.0233	-0.0898	0.0231	0.0233	-0.0896	0.0220	0.0225	0.0007	0.0005	0.0001
14	-0.0858	0.0235	0.0237	-0.0837	0.0234	0.0237	-0.0830	0.0223	0.0229	-0.0837	0.0233	0.0234	-0.0817	0.0232	0.0234	-0.0813	0.0221	0.0226	0.0007	0.0005	0.0001
15	-0.0954	0.0234	0.0235	-0.0965	0.0233	0.0235	-0.0965	0.0222	0.0228	-0.0933	0.0231	0.0233	-0.0944	0.0230	0.0232	-0.0946	0.0219	0.0225	0.0007	0.0005	0.0001
16	-0.0921	0.0336	0.0334	-0.0916	0.0335	0.0336	-0.0912	0.0321	0.0325	-0.0902	0.0333	0.0331	-0.0898	0.0332	0.0333	-0.0894	0.0317	0.0321	0.0011	0.0008	0.0002
17	-0.0856	0.0342	0.0337	-0.0836	0.0341	0.0338	-0.0829	0.0327	0.0327	-0.0838	0.0339	0.0334	-0.0819	0.0338	0.0335	-0.0812	0.0324	0.0323	0.0011	0.0008	0.0002
18	-0.0954	0.0334	0.0334	-0.0966	0.0332	0.0335	-0.0965	0.0318	0.0325	-0.0936	0.0331	0.0331	-0.0947	0.0329	0.0332	-0.0947	0.0315	0.0321	0.0011	0.0007	0.0002
19	-0.0948	0.0240	0.0236	-0.0952	0.0238	0.0235	-0.0951	0.0221	0.0226	-0.0941	0.0238	0.0234	-0.0944	0.0235	0.0232	-0.0945	0.0219	0.0223	0.0008	0.0005	0.0001
20	-0.0884	0.0241	0.0238	-0.0868	0.0239	0.0237	-0.0866	0.0222	0.0228	-0.0877	0.0238	0.0235	-0.0861	0.0236	0.0234	-0.0860	0.0221	0.0225	0.0008	0.0005	0.0001
21	-0.0980	0.0241	0.0237	-0.1003	0.0237	0.0235	-0.1005	0.0219	0.0224	-0.0972	0.0239	0.0235	-0.0995	0.0235	0.0232	-0.0997	0.0217	0.0222	0.0008	0.0005	0.0001
22	-0.0941	0.0327	0.0335	-0.0945	0.0324	0.0334	-0.0948	0.0312	0.0322	-0.0934	0.0322	0.0331	-0.0938	0.0320	0.0330	-0.0941	0.0309	0.0319	0.0010	0.0007	0.0001
23	-0.0877	0.0336	0.0336	-0.0862	0.0334	0.0336	-0.0863	0.0322	0.0325	-0.0870	0.0330	0.0333	-0.0855	0.0329	0.0333	-0.0857	0.0318	0.0322	0.0011	0.0008	0.0001
24	-0.0973	0.0327	0.0336	-0.0997	0.0321	0.0334	-0.1003	0.0306	0.0321	-0.0966	0.0321	0.0332	-0.0990	0.0316	0.0330	-0.0996	0.0303	0.0318	0.0010	0.0007	0.0001
25	-0.0946	0.0321	0.0299	-0.0958	0.0308	0.0293	-0.0958	0.0253	0.0258	-0.0931	0.0317	0.0295	-0.0942	0.0302	0.0287	-0.0943	0.0248	0.0253	0.0010	0.0007	0.0003
26	-0.0881	0.0320	0.0299	-0.0856	0.0310	0.0294	-0.0854	0.0252	0.0259	-0.0868	0.0316	0.0294	-0.0843	0.0304	0.0288	-0.0841	0.0247	0.0254	0.0010	0.0007	0.0002
27	-0.0980	0.0326	0.0302	-0.1029	0.0312	0.0294	-0.1030	0.0253	0.0258	-0.0965	0.0321	0.0298	-0.1012	0.0306	0.0289	-0.1014	0.0247	0.0252	0.0010	0.0007	0.0003
28	-0.0934	0.0444	0.0413	-0.0948	0.0433	0.0410	-0.0950	0.0365	0.0368	-0.0922	0.0440	0.0411	-0.0935	0.0427	0.0406	-0.0938	0.0359	0.0361	0.0014	0.0010	0.0004
29	-0.0865	0.0447	0.0413	-0.0843	0.0439	0.0411	-0.0846	0.0373	0.0370	-0.0854	0.0443	0.0411	-0.0831	0.0434	0.0407	-0.0835	0.0366	0.0363	0.0014	0.0010	0.0004
30	-0.0969	0.0451	0.0416	-0.1021	0.0432	0.0412	-0.1024	0.0359	0.0368	-0.0956	0.0446	0.0414	-0.1007	0.0426	0.0408	-0.1011	0.0352	0.0360	0.0015	0.0010	0.0004
31	-0.0937	0.0246	0.0250	-0.0939	0.0242	0.0247	-0.0939	0.0227	0.0235	-0.0925	0.0243	0.0247	-0.0927	0.0239	0.0244	-0.0928	0.0223	0.0232	0.0008	0.0006	0.0001
32	-0.0876	0.0247	0.0251	-0.0855	0.0244	0.0248	-0.0852	0.0229	0.0237	-0.0865	0.0244	0.0247	-0.0845	0.0241	0.0245	-0.0843	0.0226	0.0234	0.0008	0.0006	0.0001
33	-0.0967	0.0247	0.0251	-0.0992	0.0242	0.0247	-0.0993	0.0226	0.0234	-0.0953	0.0244	0.0248	-0.0978	0.0239	0.0244	-0.0981	0.0222	0.0231	0.0008	0.0006	0.0001
34	-0.0932	0.0360	0.0355	-0.0936	0.0354	0.0353	-0.0936	0.0328	0.0336	-0.0924	0.0355	0.0350	-0.0927	0.0350	0.0348	-0.0926	0.0323	0.0331	0.0011	0.0008	0.0002
35	-0.0870	0.0360	0.0356	-0.0852	0.0355	0.0355	-0.0850	0.0332	0.0339	-0.0862	0.0356	0.0352	-0.0843	0.0352	0.0350	-0.0840	0.0327	0.0334	0.0011	0.0008	0.0002
36	-0.0963	0.0364	0.0356	-0.0990	0.0355	0.0353	-0.0992	0.0325	0.0335	-0.0954	0.0359	0.0352	-0.0980	0.0350	0.0348	-0.0981	0.0320	0.0330	0.0012	0.0008	0.0002
37	-0.0626	0.0235	0.0238	-0.0625	0.0234	0.0238	-0.0624	0.0223	0.0231	-0.0613	0.0232	0.0236	-0.0612	0.0232	0.0236	-0.0612	0.0222	0.0229	0.0007	0.0005	0.0001
38	-0.0539	0.0236	0.0240	-0.0524	0.0235	0.0239	-0.0520	0.0224	0.0232	-0.0526	0.0233	0.0238	-0.0511	0.0232	0.0237	-0.0509	0.0223	0.0231	0.0007	0.0005	0.0001
39	-0.0671	0.0235	0.0237	-0.0685	0.0235	0.0237	-0.0687	0.0224	0.0231	-0.0657	0.0232	0.0235	-0.0671	0.0232	0.0235	-0.0675	0.0222	0.0229	0.0007	0.0005	0.0001
40	-0.0626	0.0341	0.0336	-0.0624	0.0340	0.0337	-0.0626	0.0329	0.0329	-0.0613	0.0337	0.0334	-0.0611	0.0336	0.0334	-0.0614	0.0327	0.0327	0.0011	0.0008	0.0001
41	-0.0540	0.0340	0.0339	-0.0524	0.0339	0.0340	-0.0522	0.0327	0.0332	-0.0527	0.0336	0.0337	-0.0511	0.0335	0.0337	-0.0510	0.0325	0.0329	0.0011	0.0008	0.0001
42	-0.0674	0.0337	0.0335	-0.0688	0.0336	0.0336	-0.0692	0.0325	0.0328	-0.0661	0.0333	0.0332	-0.0674	0.0332	0.0333	-0.0680	0.0323	0.0326	0.0011	0.0008	0.0001
43	-0.0621	0.0301	0.0286	-0.0615	0.0301	0.0285	-0.0611	0.0247	0.0255	-0.0595	0.0296	0.0282	-0.0588	0.0293	0.0280	-0.0588	0.0243	0.0251	0.0010	0.0007	0.0003
44	-0.0532	0.0295	0.0285	-0.0494	0.0289	0.0282	-0.0486	0.0245	0.0254	-0.0506	0.0290	0.0282	-0.0467	0.0283	0.0278	-0.0464	0.0241	0.0250	0.0009	0.0007	0.0002
45	-0.0667	0.0305	0.0286	-0.0695	0.0305	0.0287	-0.0699	0.0252	0.0257	-0.0640	0.0299	0.0282	-0.0667	0.0297	0.0282	-0.0675	0.0247	0.0253	0.0010	0.0007	0.0003
46	-0.0620	0.0432	0.0396	-0.0613	0.0428	0.0396	-0.0605	0.0371	0.0363	-0.0596	0.0428	0.0394	-0.0589	0.0425	0.0393	-0.0584	0.0365	0.0357	0.0014	0.0010	0.0003
47	-0.0530	0.0431	0.0398	-0.0492	0.0423	0.0395	-0.0478	0.0364	0.0362	-0.0507	0.0427	0.0395	-0.0469	0.0420	0.0392	-0.0457	0.0358	0.0356	0.0014	0.0010	0.0003
48	-0.0670	0.0431	0.0396	-0.0698	0.0429	0.0398	-0.0695	0.0371	0.0366	-0.0646	0.0426	0.0394	-0.0673	0.0424	0.0395	-0.0674	0.0365	0.0360	0.0014	0.0010	0.0003
49	-0.0601	0.0246	0.0246	-0.0594	0.0245	0.0245	-0.0590	0.0233	0.0237	-0.0580	0.0244	0.0243	-0.0573	0.0243	0.0243	-0.0572	0.0231	0.0235	0.0008	0.0006	0.0001
50	-0.0514	0.0248	0.0248	-0.0488	0.0246	0.0247	-0.0481	0.0234	0.0238	-0.0494	0.0246	0.0245	-0.0469	0.0244	0.0244	-0.0464	0.0232	0.0236	0.0008	0.0006	0.0001
51	-0.0646	0.0246	0.0246	-0.0658	0.0245	0.0246	-0.0658	0.0233	0.0238	-0.0624	0.0244	0.0243	-0.0636	0.0243	0.0243	-0.0639	0.0231	0.0235	0.0008	0.0006	0.0001
52	-0.0600	0.0355	0.0350	-0.0592	0.0353	0.0350	-0.0588	0.0340	0.0339	-0.0582	0.0353	0.0347	-0.0574	0.0350	0.0347	-0.0571	0.0336	0.0335	0.0011	0.0008	0.0002
53	-0.0513	0.0357	0.0353	-0.0487	0.0354	0.0352	-0.0479	0.0340	0.0341	-0.0495	0.0354	0.0350	-0.0470	0.0351	0.0349	-0.0462	0.0336	0.0337	0.0011	0.0008	0.0002
54	-0.0649	0.0354	0.0349	-0.0660	0.0352	0.0350	-0.0660	0.0338	0.0339	-0.0630	0.0350	0.0346	-0.0641	0.0348	0.0347	-0.0641	0.0335	0.0335	0.0011	0.0008	0.0002
55	-0.0629	0.0255	0.0251	-0.0631	0.0251	0.0248	-0.0630	0.0233	0.0238	-0.0623	0.0252	0.0248	-0.0626	0.0249	0.0246	-0.0626	0.0231	0.0236	0.0008	0.0006	0.0001
56	-0.0540	0.0254	0.0252	-0.0519	0.0251	0.0250	-0.0515	0.0235	0.0240	-0.0535	0.0251	0.0249	-0.0514	0.0248	0.0247	-0.0512	0.0233	0.0238	0.0008	0.0006	0.0001
57	-0.0674	0.0255	0.0252	-0.0701	0.0251	0.0249	-0.0703	0.0233	0.0238	-0.0668	0.0252	0.0249	-0.0695	0.0248	0.0246	-0.0697	0.0231	0.0235	0.0008	0.0006	0.0001
58	-0.0623	0.0358	0.0355	-0.0626	0.0353	0.0353	-0.0629	0.0339	0.0341	-0.0619	0.0352	0.0351	-0.0621	0.0348	0.0349	-0.0625	0.0336	0.0337	0.0011	0.0008	0.0001
59	-0.0532	0.0360	0.0357	-0.0512	0.0356	0.0356	-0.0512	0.0341	0.0344	-0.0528	0.0353	0.0354	-0.0508	0.0350	0.0352	-0.0509	0.0337	0.0340	0.0011	0.0008	0.0001
60	-0.0669	0.0358	0.0355	-0.0696	0.0352	0.0352	-0.0701	0.0337	0.0339	-0.0664	0.0352	0.0352	-0.0690	0.0346	0.0349	-0.0696	0.0333	0.0335	0.0011	0.0008	0.0001
61	-0.0627	0.0343	0.0318	-0.0636	0.0326	0.0308	-0.0635	0.0263	0.0270	-0.0618	0.0339	0.0313	-0.0626	0.0320	0.0302	-0.0625	0.0258	0.0264	0.0011	0.0008	0.0003
62	-0.0534	0.0338	0.0316	-0.0498	0.0320	0.0307	-0.0499	0.0263	0.0271	-0.0527	0.0332	0.0312	-0.0491	0.0313	0.0301	-0.0491	0.0258	0.0266	0.0011	0.0008	0.0003
63	-0.0681	0.0341	0.0321	-0.0735	0.0321	0.0309	-0.0737	0.0265	0.0270	-0.0670	0.0335	0.0316	-0.0723	0.0315	0.0303	-0.0725	0.0259	0.0265	0.0011	0.0008	0.0003
64	-0.0613	0.0477	0.0439	-0.0622	0.0458	0.0432	-0.0629	0.0385	0.0385	-0.0605	0.0472	0.0437	-0.0613	0.0453	0.0428	-0.0621	0.0379	0.0377	0.0015	0.0011	0.0004
65	-0.0522	0.0473	0.0438	-0.0486	0.0456	0.0431	-0.0491	0.0386	0.0386	-0.0514	0.0470	0.0436	-0.0478	0.0452	0.0427	-0.0484	0.0379	0.0379	0.0015	0.0011	0.0004
66	-0.0665	0.0483	0.0443	-0.0720	0.0462	0.0434	-0.0728	0.0384	0.0386	-0.0654	0.0476	0.0442	-0.0709	0.0454	0.0431	-0.0719	0.0377	0.0378	0.0015	0.0011	0.0004
67	-0.0624	0.0261	0.0264	-0.0626	0.0257	0.0261	-0.0625	0.0241	0.0248	-0.0615	0.0257	0.0261	-0.0617	0.0253	0.0257	-0.0617	0.0237	0.0244	0.0008	0.0006	0.0001
68	-0.0540	0.0263	0.0265	-0.0514	0.0259	0.0262	-0.0510	0.0243	0.0249	-0.0532	0.0259	0.0262	-0.0506	0.0255	0.0258	-0.0504	0.0239	0.0246	0.0008	0.0006	0.0001
69	-0.0665	0.0263	0.0266	-0.0696	0.0257	0.0262	-0.0698	0.0241	0.0248	-0.0654	0.0260	0.0263	-0.0684	0.0254	0.0258	-0.0689	0.0238	0.0245	0.0008	0.0006	0.0001
70	-0.0617	0.0387	0.0377	-0.0619	0.0379	0.0374	-0.0622	0.0348	0.0355	-0.0611	0.0381	0.0373	-0.0613	0.0373	0.0369	-0.0615	0.0343	0.0350	0.0012	0.0009	0.0002
71	-0.0534	0.0387	0.0378	-0.0510	0.0379	0.0375	-0.0509	0.0350	0.0357	-0.0530	0.0381	0.0373	-0.0505	0.0374	0.0370	-0.0503	0.0344	0.0352	0.0012	0.0009	0.0002
72	-0.0662	0.0390	0.0379	-0.0693	0.0379	0.0374	-0.0699	0.0346	0.0355	-0.0654	0.0384	0.0375	-0.0685	0.0374	0.0370	-0.0690	0.0341	0.0349	0.0012	0.0009	0.0002

Table B6. Table for estimates by IPCW when the outcome analysis model accounts for time-varying treatment effect and does covariate adjustment.

	IPCWu_NUC			IPCWs_t_NUC			IPCWs_tL_NUC			IPCWu_RC			IPCWs_t_RC			IPCWs_tL_RC			Max_MCSE		
	Estimate	EmpSE	ModSE	Estimate	EmpSE	ModSE	Estimate	EmpSE	ModSE	Estimate	EmpSE	ModSE	Estimate	EmpSE	ModSE	Estimate	EmpSE	ModSE	Mean	EmpSE	ModSE
1	-0.0947	0.0225	0.0228	-0.0953	0.0227	0.0230	-0.0954	0.0215	0.0222	-0.0934	0.0223	0.0226	-0.0939	0.0224	0.0228	-0.0942	0.0213	0.0221	0.0007	0.0005	0.0001
2	-0.0891	0.0227	0.0230	-0.0897	0.0228	0.0232	-0.0898	0.0216	0.0224	-0.0877	0.0224	0.0228	-0.0883	0.0225	0.0230	-0.0886	0.0214	0.0222	0.0007	0.0005	0.0001
3	-0.0971	0.0223	0.0227	-0.0977	0.0225	0.0229	-0.0978	0.0213	0.0221	-0.0957	0.0221	0.0225	-0.0963	0.0222	0.0227	-0.0965	0.0211	0.0219	0.0007	0.0005	0.0001
4	-0.0948	0.0320	0.0323	-0.0955	0.0322	0.0326	-0.0957	0.0309	0.0318	-0.0935	0.0316	0.0320	-0.0941	0.0318	0.0323	-0.0945	0.0307	0.0314	0.0010	0.0007	0.0001
5	-0.0891	0.0328	0.0326	-0.0898	0.0330	0.0329	-0.0900	0.0317	0.0320	-0.0878	0.0324	0.0323	-0.0884	0.0326	0.0326	-0.0888	0.0315	0.0317	0.0010	0.0007	0.0001
6	-0.0973	0.0318	0.0321	-0.0980	0.0321	0.0324	-0.0982	0.0307	0.0316	-0.0960	0.0315	0.0318	-0.0966	0.0317	0.0321	-0.0970	0.0305	0.0313	0.0010	0.0007	0.0001
7	-0.0941	0.0292	0.0279	-0.0960	0.0302	0.0288	-0.0967	0.0249	0.0256	-0.0914	0.0288	0.0275	-0.0932	0.0296	0.0283	-0.0943	0.0244	0.0251	0.0010	0.0007	0.0003
8	-0.0886	0.0299	0.0282	-0.0905	0.0313	0.0292	-0.0910	0.0250	0.0257	-0.0859	0.0294	0.0278	-0.0877	0.0305	0.0287	-0.0886	0.0245	0.0252	0.0010	0.0007	0.0004
9	-0.0965	0.0291	0.0278	-0.0984	0.0301	0.0287	-0.0991	0.0249	0.0255	-0.0938	0.0288	0.0274	-0.0956	0.0296	0.0282	-0.0967	0.0244	0.0250	0.0010	0.0007	0.0003
10	-0.0941	0.0419	0.0388	-0.0963	0.0432	0.0402	-0.0961	0.0365	0.0365	-0.0917	0.0413	0.0386	-0.0939	0.0426	0.0399	-0.0939	0.0358	0.0357	0.0014	0.0010	0.0004
11	-0.0884	0.0428	0.0391	-0.0907	0.0441	0.0405	-0.0904	0.0373	0.0367	-0.0860	0.0422	0.0389	-0.0882	0.0435	0.0402	-0.0882	0.0366	0.0360	0.0014	0.0010	0.0004
12	-0.0965	0.0416	0.0387	-0.0988	0.0429	0.0401	-0.0985	0.0364	0.0364	-0.0942	0.0410	0.0385	-0.0963	0.0423	0.0398	-0.0963	0.0357	0.0356	0.0014	0.0010	0.0004
13	-0.0925	0.0236	0.0238	-0.0929	0.0238	0.0240	-0.0928	0.0225	0.0231	-0.0904	0.0233	0.0235	-0.0908	0.0235	0.0237	-0.0909	0.0223	0.0228	0.0008	0.0005	0.0001
14	-0.0867	0.0238	0.0239	-0.0871	0.0239	0.0242	-0.0870	0.0226	0.0233	-0.0846	0.0235	0.0236	-0.0849	0.0237	0.0238	-0.0851	0.0224	0.0230	0.0008	0.0005	0.0001
15	-0.0948	0.0235	0.0237	-0.0952	0.0237	0.0239	-0.0951	0.0224	0.0230	-0.0927	0.0233	0.0234	-0.0930	0.0234	0.0236	-0.0932	0.0222	0.0227	0.0008	0.0005	0.0001
16	-0.0922	0.0339	0.0337	-0.0927	0.0342	0.0341	-0.0925	0.0327	0.0330	-0.0904	0.0335	0.0334	-0.0908	0.0338	0.0338	-0.0907	0.0322	0.0325	0.0011	0.0008	0.0002
17	-0.0866	0.0346	0.0340	-0.0871	0.0348	0.0344	-0.0869	0.0333	0.0333	-0.0847	0.0342	0.0337	-0.0851	0.0345	0.0341	-0.0850	0.0329	0.0328	0.0011	0.0008	0.0002
18	-0.0947	0.0337	0.0336	-0.0952	0.0339	0.0340	-0.0950	0.0324	0.0329	-0.0929	0.0333	0.0333	-0.0933	0.0336	0.0337	-0.0932	0.0320	0.0324	0.0011	0.0008	0.0002
19	-0.0949	0.0242	0.0238	-0.0954	0.0244	0.0240	-0.0954	0.0224	0.0229	-0.0941	0.0240	0.0236	-0.0946	0.0241	0.0238	-0.0947	0.0222	0.0226	0.0008	0.0006	0.0001
20	-0.0892	0.0243	0.0240	-0.0897	0.0245	0.0242	-0.0897	0.0224	0.0230	-0.0885	0.0240	0.0237	-0.0889	0.0242	0.0239	-0.0890	0.0223	0.0228	0.0008	0.0005	0.0001
21	-0.0973	0.0244	0.0239	-0.0978	0.0245	0.0241	-0.0978	0.0222	0.0228	-0.0965	0.0241	0.0237	-0.0970	0.0242	0.0238	-0.0971	0.0221	0.0226	0.0008	0.0005	0.0001
22	-0.0942	0.0331	0.0338	-0.0948	0.0333	0.0342	-0.0950	0.0318	0.0328	-0.0935	0.0325	0.0335	-0.0940	0.0328	0.0338	-0.0943	0.0315	0.0324	0.0011	0.0007	0.0002
23	-0.0886	0.0339	0.0340	-0.0892	0.0341	0.0343	-0.0894	0.0326	0.0330	-0.0879	0.0333	0.0336	-0.0884	0.0336	0.0340	-0.0887	0.0322	0.0326	0.0011	0.0008	0.0002
24	-0.0964	0.0331	0.0340	-0.0971	0.0334	0.0343	-0.0974	0.0315	0.0328	-0.0957	0.0326	0.0336	-0.0963	0.0328	0.0339	-0.0967	0.0312	0.0324	0.0011	0.0008	0.0002
25	-0.0945	0.0328	0.0307	-0.0961	0.0337	0.0316	-0.0961	0.0261	0.0268	-0.0931	0.0324	0.0302	-0.0945	0.0332	0.0310	-0.0946	0.0256	0.0262	0.0011	0.0008	0.0003
26	-0.0890	0.0332	0.0307	-0.0905	0.0345	0.0317	-0.0905	0.0261	0.0269	-0.0877	0.0325	0.0302	-0.0890	0.0336	0.0311	-0.0890	0.0255	0.0263	0.0011	0.0008	0.0004
27	-0.0970	0.0337	0.0311	-0.0987	0.0347	0.0321	-0.0986	0.0262	0.0269	-0.0956	0.0331	0.0306	-0.0972	0.0340	0.0315	-0.0971	0.0256	0.0263	0.0011	0.0008	0.0004
28	-0.0935	0.0459	0.0427	-0.0953	0.0473	0.0443	-0.0953	0.0379	0.0384	-0.0921	0.0453	0.0424	-0.0938	0.0467	0.0439	-0.0940	0.0373	0.0375	0.0015	0.0011	0.0005
29	-0.0877	0.0461	0.0427	-0.0893	0.0474	0.0443	-0.0895	0.0385	0.0385	-0.0864	0.0456	0.0424	-0.0879	0.0470	0.0439	-0.0883	0.0378	0.0377	0.0015	0.0011	0.0005
30	-0.0956	0.0470	0.0432	-0.0976	0.0484	0.0448	-0.0977	0.0379	0.0385	-0.0943	0.0462	0.0429	-0.0961	0.0476	0.0444	-0.0964	0.0373	0.0376	0.0016	0.0011	0.0005
31	-0.0938	0.0249	0.0253	-0.0943	0.0251	0.0255	-0.0942	0.0231	0.0240	-0.0925	0.0245	0.0250	-0.0929	0.0247	0.0252	-0.0931	0.0227	0.0236	0.0008	0.0006	0.0001
32	-0.0884	0.0250	0.0253	-0.0889	0.0252	0.0256	-0.0889	0.0233	0.0241	-0.0873	0.0246	0.0250	-0.0877	0.0248	0.0252	-0.0878	0.0230	0.0238	0.0008	0.0006	0.0001
33	-0.0959	0.0251	0.0254	-0.0964	0.0252	0.0256	-0.0963	0.0232	0.0240	-0.0946	0.0246	0.0250	-0.0950	0.0248	0.0252	-0.0952	0.0228	0.0236	0.0008	0.0006	0.0001
34	-0.0933	0.0365	0.0360	-0.0938	0.0368	0.0365	-0.0938	0.0336	0.0344	-0.0924	0.0359	0.0355	-0.0929	0.0362	0.0359	-0.0927	0.0331	0.0338	0.0012	0.0008	0.0002
35	-0.0880	0.0363	0.0360	-0.0885	0.0367	0.0365	-0.0884	0.0338	0.0346	-0.0870	0.0360	0.0356	-0.0875	0.0363	0.0360	-0.0873	0.0333	0.0340	0.0012	0.0008	0.0002
36	-0.0955	0.0370	0.0362	-0.0960	0.0373	0.0366	-0.0960	0.0336	0.0344	-0.0946	0.0363	0.0357	-0.0951	0.0366	0.0361	-0.0949	0.0330	0.0338	0.0012	0.0008	0.0003
37	-0.0627	0.0237	0.0240	-0.0632	0.0238	0.0241	-0.0633	0.0225	0.0233	-0.0613	0.0234	0.0238	-0.0619	0.0235	0.0239	-0.0621	0.0224	0.0231	0.0008	0.0005	0.0001
38	-0.0550	0.0238	0.0242	-0.0556	0.0239	0.0244	-0.0557	0.0227	0.0236	-0.0537	0.0235	0.0240	-0.0542	0.0236	0.0241	-0.0545	0.0226	0.0234	0.0008	0.0005	0.0001
39	-0.0660	0.0237	0.0239	-0.0666	0.0238	0.0240	-0.0667	0.0226	0.0233	-0.0647	0.0234	0.0237	-0.0652	0.0235	0.0238	-0.0655	0.0224	0.0231	0.0008	0.0005	0.0001
40	-0.0627	0.0345	0.0339	-0.0633	0.0347	0.0342	-0.0636	0.0334	0.0333	-0.0614	0.0341	0.0337	-0.0620	0.0342	0.0339	-0.0624	0.0332	0.0330	0.0011	0.0008	0.0001
41	-0.0552	0.0344	0.0343	-0.0558	0.0346	0.0346	-0.0560	0.0333	0.0337	-0.0538	0.0340	0.0340	-0.0544	0.0342	0.0343	-0.0548	0.0330	0.0334	0.0011	0.0008	0.0002
42	-0.0664	0.0341	0.0337	-0.0670	0.0343	0.0340	-0.0673	0.0330	0.0332	-0.0650	0.0336	0.0335	-0.0656	0.0338	0.0337	-0.0661	0.0327	0.0328	0.0011	0.0008	0.0001
43	-0.0622	0.0309	0.0292	-0.0641	0.0323	0.0301	-0.0645	0.0257	0.0264	-0.0595	0.0303	0.0287	-0.0613	0.0314	0.0296	-0.0622	0.0252	0.0259	0.0010	0.0007	0.0004
44	-0.0544	0.0302	0.0292	-0.0562	0.0311	0.0300	-0.0570	0.0257	0.0266	-0.0517	0.0298	0.0288	-0.0534	0.0305	0.0296	-0.0546	0.0253	0.0261	0.0010	0.0007	0.0003
45	-0.0656	0.0310	0.0291	-0.0674	0.0324	0.0301	-0.0679	0.0258	0.0264	-0.0628	0.0304	0.0286	-0.0646	0.0315	0.0295	-0.0655	0.0253	0.0259	0.0010	0.0007	0.0004
46	-0.0622	0.0442	0.0405	-0.0643	0.0454	0.0418	-0.0641	0.0387	0.0377	-0.0598	0.0437	0.0402	-0.0618	0.0450	0.0414	-0.0620	0.0380	0.0371	0.0014	0.0010	0.0004
47	-0.0546	0.0443	0.0408	-0.0566	0.0455	0.0421	-0.0562	0.0384	0.0380	-0.0521	0.0438	0.0406	-0.0542	0.0450	0.0418	-0.0541	0.0377	0.0373	0.0014	0.0010	0.0004
48	-0.0658	0.0439	0.0403	-0.0679	0.0451	0.0416	-0.0676	0.0383	0.0376	-0.0634	0.0433	0.0400	-0.0655	0.0445	0.0413	-0.0655	0.0377	0.0369	0.0014	0.0010	0.0004
49	-0.0603	0.0249	0.0249	-0.0607	0.0250	0.0251	-0.0606	0.0237	0.0241	-0.0581	0.0246	0.0246	-0.0585	0.0248	0.0247	-0.0587	0.0235	0.0238	0.0008	0.0006	0.0001
50	-0.0526	0.0251	0.0251	-0.0530	0.0252	0.0253	-0.0529	0.0239	0.0243	-0.0505	0.0248	0.0248	-0.0508	0.0250	0.0250	-0.0510	0.0237	0.0241	0.0008	0.0006	0.0001
51	-0.0636	0.0249	0.0248	-0.0640	0.0250	0.0250	-0.0640	0.0237	0.0241	-0.0615	0.0246	0.0245	-0.0618	0.0248	0.0247	-0.0621	0.0234	0.0238	0.0008	0.0006	0.0001
52	-0.0602	0.0359	0.0354	-0.0606	0.0362	0.0358	-0.0604	0.0347	0.0345	-0.0583	0.0356	0.0351	-0.0587	0.0359	0.0354	-0.0586	0.0343	0.0341	0.0011	0.0008	0.0002
53	-0.0526	0.0362	0.0358	-0.0531	0.0364	0.0361	-0.0527	0.0349	0.0349	-0.0508	0.0358	0.0355	-0.0511	0.0361	0.0358	-0.0509	0.0345	0.0344	0.0012	0.0008	0.0002
54	-0.0639	0.0358	0.0352	-0.0643	0.0360	0.0356	-0.0641	0.0345	0.0343	-0.0620	0.0354	0.0349	-0.0624	0.0356	0.0353	-0.0623	0.0341	0.0339	0.0011	0.0008	0.0002
55	-0.0629	0.0258	0.0254	-0.0633	0.0259	0.0256	-0.0632	0.0237	0.0242	-0.0623	0.0255	0.0251	-0.0627	0.0257	0.0253	-0.0627	0.0235	0.0240	0.0008	0.0006	0.0001
56	-0.0551	0.0257	0.0255	-0.0554	0.0258	0.0257	-0.0553	0.0238	0.0244	-0.0546	0.0254	0.0252	-0.0549	0.0255	0.0254	-0.0549	0.0236	0.0242	0.0008	0.0006	0.0001
57	-0.0663	0.0258	0.0255	-0.0668	0.0260	0.0257	-0.0667	0.0237	0.0242	-0.0658	0.0255	0.0252	-0.0662	0.0257	0.0254	-0.0662	0.0235	0.0240	0.0008	0.0006	0.0001
58	-0.0624	0.0363	0.0360	-0.0628	0.0366	0.0363	-0.0631	0.0346	0.0347	-0.0619	0.0357	0.0356	-0.0623	0.0359	0.0359	-0.0626	0.0343	0.0343	0.0012	0.0008	0.0002
59	-0.0544	0.0365	0.0362	-0.0548	0.0368	0.0366	-0.0550	0.0348	0.0350	-0.0540	0.0358	0.0358	-0.0543	0.0360	0.0361	-0.0546	0.0344	0.0346	0.0012	0.0008	0.0002
60	-0.0658	0.0364	0.0360	-0.0663	0.0366	0.0363	-0.0666	0.0346	0.0347	-0.0653	0.0357	0.0356	-0.0657	0.0359	0.0359	-0.0661	0.0342	0.0342	0.0012	0.0008	0.0002
61	-0.0627	0.0357	0.0329	-0.0639	0.0369	0.0339	-0.0638	0.0274	0.0282	-0.0618	0.0351	0.0323	-0.0629	0.0361	0.0332	-0.0628	0.0269	0.0276	0.0012	0.0008	0.0004
62	-0.0546	0.0348	0.0326	-0.0556	0.0357	0.0335	-0.0560	0.0274	0.0283	-0.0538	0.0341	0.0321	-0.0546	0.0349	0.0329	-0.0551	0.0269	0.0277	0.0011	0.0008	0.0003
63	-0.0667	0.0351	0.0331	-0.0680	0.0358	0.0341	-0.0679	0.0276	0.0284	-0.0657	0.0345	0.0326	-0.0669	0.0352	0.0334	-0.0668	0.0271	0.0277	0.0012	0.0008	0.0003
64	-0.0613	0.0496	0.0456	-0.0625	0.0511	0.0474	-0.0634	0.0402	0.0404	-0.0604	0.0489	0.0454	-0.0616	0.0504	0.0470	-0.0625	0.0395	0.0395	0.0016	0.0011	0.0005
65	-0.0537	0.0492	0.0455	-0.0547	0.0507	0.0472	-0.0552	0.0402	0.0406	-0.0527	0.0486	0.0452	-0.0537	0.0501	0.0468	-0.0544	0.0395	0.0396	0.0016	0.0011	0.0005
66	-0.0649	0.0502	0.0462	-0.0664	0.0517	0.0479	-0.0671	0.0403	0.0407	-0.0639	0.0494	0.0460	-0.0653	0.0507	0.0476	-0.0662	0.0397	0.0397	0.0016	0.0012	0.0005
67	-0.0625	0.0265	0.0268	-0.0629	0.0267	0.0270	-0.0628	0.0246	0.0254	-0.0615	0.0261	0.0265	-0.0619	0.0262	0.0266	-0.0620	0.0242	0.0250	0.0008	0.0006	0.0001
68	-0.0551	0.0266	0.0269	-0.0555	0.0268	0.0271	-0.0554	0.0248	0.0255	-0.0542	0.0262	0.0265	-0.0545	0.0264	0.0267	-0.0546	0.0244	0.0251	0.0008	0.0006	0.0001
69	-0.0654	0.0267	0.0270	-0.0658	0.0269	0.0273	-0.0658	0.0247	0.0255	-0.0643	0.0264	0.0267	-0.0647	0.0265	0.0269	-0.0649	0.0244	0.0251	0.0009	0.0006	0.0001
70	-0.0617	0.0393	0.0383	-0.0621	0.0396	0.0388	-0.0623	0.0359	0.0365	-0.0612	0.0386	0.0378	-0.0616	0.0389	0.0383	-0.0616	0.0352	0.0358	0.0013	0.0009	0.0002
71	-0.0547	0.0393	0.0384	-0.0551	0.0396	0.0389	-0.0551	0.0361	0.0367	-0.0541	0.0387	0.0379	-0.0544	0.0390	0.0384	-0.0544	0.0354	0.0360	0.0013	0.0009	0.0002
72	-0.0650	0.0397	0.0386	-0.0655	0.0399	0.0390	-0.0657	0.0359	0.0365	-0.0643	0.0390	0.0381	-0.0648	0.0392	0.0385	-0.0648	0.0352	0.0359	0.0013	0.0009	0.0002

Table B7. Table for estimates by IPCW when the outcome analysis model does not account for time-varying treatment effect and does not do covariate adjustment.

	IPCWu_NUC			IPCWs_t_NUC			IPCWs_tL_NUC			IPCWu_RC			IPCWs_t_RC			IPCWs_tL_RC			Max_MCSE		
	Estimate	EmpSE	ModSE	Estimate	EmpSE	ModSE	Estimate	EmpSE	ModSE	Estimate	EmpSE	ModSE	Estimate	EmpSE	ModSE	Estimate	EmpSE	ModSE	Mean	EmpSE	ModSE
1	-0.0943	0.0228	0.0231	-0.0943	0.0227	0.0231	-0.0907	0.0210	0.0216	-0.0930	0.0224	0.0228	-0.0930	0.0224	0.0227	-0.0896	0.0209	0.0215	0.0007	0.0005	0.0002
2	-0.0880	0.0229	0.0233	-0.0868	0.0228	0.0232	-0.0832	0.0211	0.0218	-0.0867	0.0226	0.0230	-0.0855	0.0225	0.0229	-0.0822	0.0210	0.0216	0.0007	0.0005	0.0002
3	-0.0974	0.0226	0.0230	-0.0986	0.0225	0.0230	-0.0949	0.0208	0.0215	-0.0960	0.0222	0.0227	-0.0973	0.0222	0.0227	-0.0938	0.0207	0.0213	0.0007	0.0005	0.0002
4	-0.0939	0.0322	0.0327	-0.0938	0.0320	0.0327	-0.0902	0.0298	0.0307	-0.0925	0.0317	0.0325	-0.0924	0.0315	0.0324	-0.0891	0.0296	0.0305	0.0010	0.0007	0.0003
5	-0.0875	0.0329	0.0330	-0.0863	0.0327	0.0329	-0.0828	0.0306	0.0310	-0.0862	0.0324	0.0327	-0.0850	0.0323	0.0327	-0.0817	0.0304	0.0308	0.0010	0.0007	0.0003
6	-0.0971	0.0320	0.0326	-0.0983	0.0318	0.0326	-0.0946	0.0295	0.0305	-0.0957	0.0315	0.0323	-0.0969	0.0313	0.0323	-0.0935	0.0293	0.0303	0.0010	0.0007	0.0003
7	-0.0934	0.0301	0.0293	-0.0934	0.0306	0.0292	-0.0872	0.0228	0.0235	-0.0910	0.0300	0.0288	-0.0906	0.0292	0.0283	-0.0851	0.0225	0.0232	0.0010	0.0007	0.0006
8	-0.0873	0.0314	0.0297	-0.0842	0.0304	0.0291	-0.0781	0.0228	0.0236	-0.0846	0.0301	0.0289	-0.0815	0.0290	0.0283	-0.0761	0.0226	0.0233	0.0010	0.0007	0.0006
9	-0.0965	0.0301	0.0293	-0.0991	0.0295	0.0289	-0.0930	0.0228	0.0235	-0.0941	0.0301	0.0288	-0.0966	0.0294	0.0284	-0.0909	0.0225	0.0231	0.0010	0.0007	0.0005
10	-0.0933	0.0451	0.0419	-0.0929	0.0441	0.0414	-0.0857	0.0330	0.0334	-0.0906	0.0432	0.0409	-0.0905	0.0438	0.0410	-0.0838	0.0326	0.0329	0.0014	0.0010	0.0010
11	-0.0870	0.0459	0.0421	-0.0838	0.0447	0.0414	-0.0769	0.0336	0.0335	-0.0842	0.0439	0.0410	-0.0815	0.0445	0.0410	-0.0749	0.0331	0.0331	0.0015	0.0010	0.0010
12	-0.0966	0.0452	0.0419	-0.0991	0.0441	0.0415	-0.0917	0.0327	0.0333	-0.0939	0.0432	0.0409	-0.0967	0.0438	0.0411	-0.0897	0.0323	0.0329	0.0014	0.0010	0.0010
13	-0.0917	0.0238	0.0240	-0.0911	0.0237	0.0239	-0.0854	0.0215	0.0221	-0.0899	0.0236	0.0237	-0.0893	0.0234	0.0236	-0.0840	0.0214	0.0218	0.0008	0.0005	0.0001
14	-0.0852	0.0240	0.0241	-0.0831	0.0238	0.0240	-0.0776	0.0217	0.0222	-0.0834	0.0238	0.0238	-0.0814	0.0236	0.0237	-0.0761	0.0216	0.0220	0.0008	0.0005	0.0001
15	-0.0947	0.0238	0.0239	-0.0957	0.0236	0.0238	-0.0900	0.0214	0.0220	-0.0928	0.0235	0.0236	-0.0938	0.0234	0.0235	-0.0884	0.0213	0.0218	0.0008	0.0005	0.0001
16	-0.0910	0.0352	0.0350	-0.0903	0.0349	0.0348	-0.0845	0.0310	0.0314	-0.0893	0.0342	0.0346	-0.0887	0.0339	0.0344	-0.0830	0.0306	0.0311	0.0011	0.0008	0.0008
17	-0.0846	0.0358	0.0352	-0.0825	0.0355	0.0350	-0.0768	0.0316	0.0316	-0.0830	0.0349	0.0349	-0.0809	0.0346	0.0346	-0.0753	0.0312	0.0313	0.0011	0.0008	0.0008
18	-0.0942	0.0351	0.0349	-0.0952	0.0347	0.0347	-0.0892	0.0306	0.0313	-0.0925	0.0340	0.0345	-0.0935	0.0337	0.0344	-0.0877	0.0303	0.0309	0.0011	0.0008	0.0008
19	-0.0951	0.0243	0.0240	-0.0954	0.0239	0.0238	-0.0931	0.0216	0.0220	-0.0943	0.0239	0.0237	-0.0946	0.0236	0.0234	-0.0925	0.0215	0.0219	0.0008	0.0005	0.0002
20	-0.0887	0.0244	0.0242	-0.0871	0.0242	0.0240	-0.0849	0.0219	0.0223	-0.0880	0.0241	0.0238	-0.0864	0.0238	0.0236	-0.0844	0.0218	0.0221	0.0008	0.0005	0.0002
21	-0.0982	0.0244	0.0241	-0.1004	0.0239	0.0238	-0.0982	0.0214	0.0219	-0.0973	0.0240	0.0238	-0.0996	0.0236	0.0234	-0.0975	0.0213	0.0217	0.0008	0.0005	0.0002
22	-0.0944	0.0332	0.0342	-0.0947	0.0327	0.0339	-0.0926	0.0304	0.0314	-0.0937	0.0326	0.0338	-0.0940	0.0322	0.0335	-0.0920	0.0301	0.0311	0.0010	0.0007	0.0003
23	-0.0882	0.0340	0.0343	-0.0866	0.0336	0.0341	-0.0845	0.0313	0.0317	-0.0874	0.0334	0.0339	-0.0858	0.0330	0.0337	-0.0840	0.0310	0.0314	0.0011	0.0008	0.0003
24	-0.0975	0.0332	0.0344	-0.0998	0.0324	0.0339	-0.0978	0.0297	0.0312	-0.0967	0.0325	0.0339	-0.0990	0.0318	0.0335	-0.0972	0.0294	0.0309	0.0011	0.0007	0.0003
25	-0.0950	0.0349	0.0325	-0.0960	0.0329	0.0311	-0.0920	0.0239	0.0245	-0.0934	0.0335	0.0316	-0.0944	0.0314	0.0302	-0.0908	0.0236	0.0241	0.0011	0.0008	0.0007
26	-0.0885	0.0347	0.0324	-0.0858	0.0327	0.0311	-0.0821	0.0241	0.0247	-0.0870	0.0332	0.0315	-0.0844	0.0312	0.0301	-0.0811	0.0238	0.0243	0.0011	0.0008	0.0006
27	-0.0981	0.0344	0.0330	-0.1028	0.0323	0.0313	-0.0987	0.0238	0.0244	-0.0965	0.0335	0.0320	-0.1011	0.0312	0.0303	-0.0975	0.0234	0.0239	0.0011	0.0008	0.0007
28	-0.0940	0.0505	0.0470	-0.0951	0.0474	0.0452	-0.0911	0.0342	0.0348	-0.0925	0.0505	0.0467	-0.0936	0.0474	0.0449	-0.0900	0.0337	0.0342	0.0016	0.0011	0.0014
29	-0.0873	0.0505	0.0467	-0.0847	0.0479	0.0451	-0.0812	0.0350	0.0351	-0.0859	0.0508	0.0465	-0.0834	0.0479	0.0448	-0.0803	0.0344	0.0345	0.0016	0.0011	0.0014
30	-0.0970	0.0527	0.0481	-0.1020	0.0486	0.0460	-0.0980	0.0334	0.0347	-0.0956	0.0521	0.0476	-0.1005	0.0480	0.0454	-0.0969	0.0329	0.0340	0.0017	0.0012	0.0015
31	-0.0938	0.0250	0.0254	-0.0940	0.0245	0.0250	-0.0906	0.0219	0.0227	-0.0927	0.0245	0.0251	-0.0929	0.0241	0.0246	-0.0897	0.0216	0.0224	0.0008	0.0006	0.0002
32	-0.0877	0.0251	0.0255	-0.0856	0.0248	0.0251	-0.0824	0.0222	0.0229	-0.0868	0.0247	0.0251	-0.0847	0.0244	0.0247	-0.0817	0.0220	0.0226	0.0008	0.0006	0.0002
33	-0.0967	0.0251	0.0255	-0.0991	0.0245	0.0250	-0.0957	0.0218	0.0225	-0.0955	0.0246	0.0251	-0.0979	0.0240	0.0246	-0.0947	0.0215	0.0223	0.0008	0.0006	0.0002
34	-0.0935	0.0380	0.0375	-0.0937	0.0370	0.0369	-0.0903	0.0314	0.0322	-0.0926	0.0369	0.0370	-0.0928	0.0359	0.0364	-0.0894	0.0310	0.0318	0.0012	0.0009	0.0009
35	-0.0875	0.0377	0.0375	-0.0855	0.0369	0.0369	-0.0821	0.0318	0.0326	-0.0865	0.0369	0.0371	-0.0846	0.0361	0.0365	-0.0813	0.0314	0.0322	0.0012	0.0008	0.0009
36	-0.0964	0.0383	0.0378	-0.0990	0.0370	0.0369	-0.0955	0.0312	0.0321	-0.0955	0.0369	0.0371	-0.0980	0.0357	0.0363	-0.0945	0.0307	0.0317	0.0012	0.0009	0.0009
37	-0.0623	0.0238	0.0241	-0.0622	0.0237	0.0240	-0.0586	0.0221	0.0227	-0.0610	0.0236	0.0239	-0.0609	0.0235	0.0238	-0.0575	0.0220	0.0226	0.0008	0.0005	0.0002
38	-0.0537	0.0239	0.0243	-0.0522	0.0238	0.0242	-0.0486	0.0223	0.0229	-0.0524	0.0237	0.0241	-0.0508	0.0236	0.0239	-0.0476	0.0222	0.0228	0.0008	0.0005	0.0002
39	-0.0666	0.0239	0.0241	-0.0681	0.0238	0.0240	-0.0644	0.0221	0.0226	-0.0653	0.0236	0.0238	-0.0667	0.0235	0.0237	-0.0633	0.0220	0.0225	0.0008	0.0005	0.0002
40	-0.0618	0.0344	0.0343	-0.0616	0.0341	0.0341	-0.0581	0.0322	0.0323	-0.0605	0.0339	0.0340	-0.0603	0.0337	0.0339	-0.0570	0.0320	0.0321	0.0011	0.0008	0.0003
41	-0.0533	0.0344	0.0346	-0.0516	0.0341	0.0344	-0.0482	0.0322	0.0326	-0.0520	0.0339	0.0343	-0.0503	0.0336	0.0342	-0.0470	0.0320	0.0324	0.0011	0.0008	0.0003
42	-0.0665	0.0340	0.0341	-0.0678	0.0337	0.0340	-0.0642	0.0317	0.0321	-0.0652	0.0335	0.0338	-0.0665	0.0333	0.0338	-0.0631	0.0315	0.0319	0.0011	0.0008	0.0003
43	-0.0616	0.0320	0.0304	-0.0609	0.0309	0.0297	-0.0547	0.0237	0.0244	-0.0589	0.0307	0.0296	-0.0581	0.0296	0.0289	-0.0527	0.0235	0.0241	0.0010	0.0007	0.0006
44	-0.0528	0.0305	0.0301	-0.0492	0.0305	0.0296	-0.0432	0.0237	0.0245	-0.0504	0.0305	0.0297	-0.0465	0.0291	0.0288	-0.0412	0.0235	0.0242	0.0010	0.0007	0.0006
45	-0.0659	0.0323	0.0304	-0.0687	0.0313	0.0299	-0.0624	0.0239	0.0245	-0.0632	0.0310	0.0296	-0.0659	0.0300	0.0291	-0.0603	0.0236	0.0242	0.0010	0.0007	0.0006
46	-0.0613	0.0468	0.0430	-0.0604	0.0454	0.0422	-0.0533	0.0349	0.0347	-0.0590	0.0466	0.0427	-0.0580	0.0452	0.0418	-0.0514	0.0345	0.0343	0.0015	0.0010	0.0010
47	-0.0526	0.0467	0.0432	-0.0485	0.0450	0.0421	-0.0417	0.0347	0.0349	-0.0502	0.0465	0.0428	-0.0462	0.0447	0.0418	-0.0398	0.0343	0.0344	0.0015	0.0010	0.0010
48	-0.0660	0.0466	0.0430	-0.0686	0.0454	0.0424	-0.0613	0.0348	0.0347	-0.0633	0.0446	0.0420	-0.0662	0.0451	0.0420	-0.0593	0.0344	0.0343	0.0015	0.0010	0.0010
49	-0.0595	0.0250	0.0250	-0.0588	0.0248	0.0248	-0.0532	0.0227	0.0231	-0.0577	0.0249	0.0247	-0.0569	0.0247	0.0245	-0.0517	0.0226	0.0229	0.0008	0.0006	0.0001
50	-0.0509	0.0252	0.0252	-0.0483	0.0249	0.0249	-0.0429	0.0230	0.0233	-0.0491	0.0251	0.0249	-0.0466	0.0248	0.0246	-0.0414	0.0229	0.0231	0.0008	0.0006	0.0001
51	-0.0638	0.0250	0.0249	-0.0650	0.0248	0.0248	-0.0593	0.0227	0.0231	-0.0620	0.0248	0.0247	-0.0631	0.0246	0.0245	-0.0578	0.0226	0.0229	0.0008	0.0006	0.0001
52	-0.0589	0.0370	0.0364	-0.0581	0.0366	0.0361	-0.0523	0.0329	0.0329	-0.0573	0.0361	0.0360	-0.0564	0.0357	0.0358	-0.0508	0.0326	0.0326	0.0012	0.0008	0.0007
53	-0.0504	0.0373	0.0367	-0.0477	0.0367	0.0364	-0.0420	0.0331	0.0332	-0.0487	0.0363	0.0363	-0.0461	0.0358	0.0360	-0.0406	0.0327	0.0329	0.0012	0.0008	0.0008
54	-0.0636	0.0368	0.0363	-0.0647	0.0364	0.0361	-0.0588	0.0328	0.0328	-0.0619	0.0358	0.0359	-0.0630	0.0354	0.0357	-0.0573	0.0324	0.0325	0.0012	0.0008	0.0007
55	-0.0631	0.0258	0.0255	-0.0633	0.0254	0.0251	-0.0617	0.0231	0.0233	-0.0625	0.0255	0.0251	-0.0628	0.0251	0.0248	-0.0613	0.0229	0.0231	0.0008	0.0006	0.0002
56	-0.0544	0.0256	0.0255	-0.0522	0.0253	0.0252	-0.0506	0.0232	0.0236	-0.0539	0.0253	0.0252	-0.0517	0.0250	0.0249	-0.0503	0.0231	0.0234	0.0008	0.0006	0.0002
57	-0.0675	0.0258	0.0256	-0.0702	0.0254	0.0252	-0.0686	0.0230	0.0233	-0.0669	0.0255	0.0252	-0.0696	0.0250	0.0248	-0.0682	0.0229	0.0231	0.0008	0.0006	0.0002
58	-0.0627	0.0363	0.0362	-0.0629	0.0356	0.0358	-0.0615	0.0331	0.0332	-0.0622	0.0356	0.0358	-0.0624	0.0350	0.0354	-0.0612	0.0328	0.0329	0.0012	0.0008	0.0003
59	-0.0537	0.0366	0.0364	-0.0516	0.0360	0.0361	-0.0503	0.0334	0.0336	-0.0532	0.0358	0.0360	-0.0511	0.0352	0.0356	-0.0499	0.0331	0.0333	0.0012	0.0008	0.0003
60	-0.0671	0.0362	0.0363	-0.0697	0.0354	0.0357	-0.0684	0.0328	0.0331	-0.0665	0.0354	0.0358	-0.0691	0.0347	0.0353	-0.0680	0.0325	0.0328	0.0012	0.0008	0.0003
61	-0.0629	0.0362	0.0346	-0.0637	0.0337	0.0327	-0.0610	0.0254	0.0259	-0.0619	0.0351	0.0335	-0.0626	0.0325	0.0317	-0.0602	0.0250	0.0254	0.0012	0.0008	0.0007
62	-0.0540	0.0364	0.0343	-0.0502	0.0338	0.0326	-0.0481	0.0255	0.0261	-0.0530	0.0348	0.0334	-0.0493	0.0323	0.0316	-0.0474	0.0251	0.0257	0.0012	0.0008	0.0007
63	-0.0682	0.0362	0.0350	-0.0737	0.0337	0.0330	-0.0706	0.0253	0.0258	-0.0670	0.0350	0.0339	-0.0724	0.0325	0.0319	-0.0696	0.0250	0.0254	0.0012	0.0008	0.0008
64	-0.0613	0.0546	0.0505	-0.0621	0.0506	0.0480	-0.0603	0.0364	0.0368	-0.0605	0.0538	0.0497	-0.0613	0.0497	0.0473	-0.0596	0.0358	0.0361	0.0017	0.0012	0.0014
65	-0.0525	0.0543	0.0500	-0.0487	0.0504	0.0477	-0.0472	0.0369	0.0370	-0.0516	0.0536	0.0494	-0.0479	0.0496	0.0470	-0.0466	0.0362	0.0364	0.0017	0.0012	0.0014
66	-0.0663	0.0551	0.0511	-0.0717	0.0509	0.0485	-0.0696	0.0361	0.0367	-0.0653	0.0541	0.0503	-0.0707	0.0498	0.0477	-0.0687	0.0356	0.0360	0.0017	0.0012	0.0014
67	-0.0626	0.0264	0.0268	-0.0627	0.0259	0.0263	-0.0604	0.0233	0.0240	-0.0618	0.0260	0.0265	-0.0619	0.0255	0.0259	-0.0597	0.0231	0.0237	0.0008	0.0006	0.0002
68	-0.0542	0.0266	0.0269	-0.0516	0.0261	0.0264	-0.0494	0.0236	0.0242	-0.0535	0.0261	0.0265	-0.0510	0.0257	0.0260	-0.0489	0.0233	0.0239	0.0008	0.0006	0.0001
69	-0.0665	0.0266	0.0271	-0.0696	0.0260	0.0264	-0.0672	0.0233	0.0239	-0.0655	0.0263	0.0267	-0.0686	0.0256	0.0261	-0.0665	0.0231	0.0236	0.0008	0.0006	0.0002
70	-0.0620	0.0403	0.0397	-0.0622	0.0391	0.0389	-0.0600	0.0335	0.0342	-0.0615	0.0391	0.0391	-0.0616	0.0380	0.0383	-0.0594	0.0330	0.0337	0.0013	0.0009	0.0008
71	-0.0538	0.0404	0.0398	-0.0513	0.0392	0.0390	-0.0493	0.0338	0.0345	-0.0533	0.0394	0.0392	-0.0508	0.0382	0.0385	-0.0487	0.0332	0.0341	0.0013	0.0009	0.0008
72	-0.0664	0.0406	0.0399	-0.0695	0.0392	0.0390	-0.0672	0.0333	0.0341	-0.0657	0.0393	0.0393	-0.0687	0.0380	0.0384	-0.0665	0.0327	0.0336	0.0013	0.0009	0.0008

Table B8. Table for estimates by IPCW when the outcome analysis model accounts for time-varying treatment effect and does not do covariate adjustment.

	IPCWu_NUC			IPCWs_t_NUC			IPCWs_tL_NUC			IPCWu_RC			IPCWs_t_RC			IPCWs_tL_RC			Max_MCSE		
	Estimate	EmpSE	ModSE	Estimate	EmpSE	ModSE	Estimate	EmpSE	ModSE	Estimate	EmpSE	ModSE	Estimate	EmpSE	ModSE	Estimate	EmpSE	ModSE	Mean	EmpSE	ModSE
1	-0.0944	0.0229	0.0232	-0.0949	0.0229	0.0233	-0.0911	0.0210	0.0217	-0.0930	0.0225	0.0229	-0.0935	0.0226	0.0230	-0.0900	0.0209	0.0216	0.0007	0.0005	0.0002
2	-0.0887	0.0230	0.0234	-0.0893	0.0231	0.0235	-0.0855	0.0212	0.0219	-0.0874	0.0227	0.0231	-0.0879	0.0228	0.0232	-0.0844	0.0211	0.0218	0.0007	0.0005	0.0002
3	-0.0967	0.0227	0.0231	-0.0973	0.0227	0.0232	-0.0935	0.0209	0.0216	-0.0954	0.0223	0.0228	-0.0959	0.0224	0.0229	-0.0924	0.0208	0.0215	0.0007	0.0005	0.0002
4	-0.0939	0.0324	0.0329	-0.0945	0.0325	0.0330	-0.0907	0.0301	0.0308	-0.0926	0.0319	0.0326	-0.0931	0.0320	0.0327	-0.0896	0.0299	0.0306	0.0010	0.0007	0.0003
5	-0.0883	0.0331	0.0332	-0.0888	0.0332	0.0333	-0.0851	0.0309	0.0311	-0.0869	0.0327	0.0329	-0.0875	0.0328	0.0330	-0.0840	0.0307	0.0309	0.0011	0.0007	0.0003
6	-0.0965	0.0322	0.0328	-0.0970	0.0323	0.0329	-0.0933	0.0298	0.0307	-0.0951	0.0317	0.0325	-0.0957	0.0318	0.0326	-0.0922	0.0297	0.0305	0.0010	0.0007	0.0003
7	-0.0935	0.0304	0.0295	-0.0951	0.0307	0.0298	-0.0888	0.0231	0.0239	-0.0910	0.0303	0.0291	-0.0926	0.0306	0.0294	-0.0868	0.0228	0.0235	0.0010	0.0007	0.0005
8	-0.0880	0.0318	0.0301	-0.0896	0.0321	0.0304	-0.0830	0.0233	0.0240	-0.0853	0.0305	0.0292	-0.0868	0.0308	0.0295	-0.0810	0.0230	0.0237	0.0010	0.0007	0.0006
9	-0.0959	0.0303	0.0294	-0.0975	0.0306	0.0298	-0.0912	0.0231	0.0238	-0.0934	0.0303	0.0290	-0.0950	0.0306	0.0293	-0.0892	0.0228	0.0234	0.0010	0.0007	0.0005
10	-0.0934	0.0456	0.0423	-0.0951	0.0460	0.0427	-0.0874	0.0336	0.0339	-0.0907	0.0436	0.0413	-0.0923	0.0439	0.0417	-0.0855	0.0332	0.0334	0.0015	0.0010	0.0010
11	-0.0878	0.0465	0.0425	-0.0895	0.0468	0.0430	-0.0817	0.0343	0.0342	-0.0850	0.0444	0.0415	-0.0871	0.0466	0.0426	-0.0798	0.0339	0.0337	0.0015	0.0010	0.0011
12	-0.0960	0.0455	0.0422	-0.0977	0.0459	0.0426	-0.0899	0.0334	0.0338	-0.0932	0.0435	0.0411	-0.0949	0.0439	0.0416	-0.0880	0.0330	0.0333	0.0015	0.0010	0.0010
13	-0.0918	0.0240	0.0241	-0.0922	0.0241	0.0242	-0.0862	0.0217	0.0222	-0.0900	0.0237	0.0238	-0.0903	0.0238	0.0239	-0.0847	0.0215	0.0220	0.0008	0.0005	0.0001
14	-0.0860	0.0242	0.0243	-0.0863	0.0243	0.0244	-0.0804	0.0219	0.0224	-0.0842	0.0240	0.0240	-0.0845	0.0240	0.0241	-0.0789	0.0217	0.0222	0.0008	0.0005	0.0001
15	-0.0941	0.0239	0.0240	-0.0945	0.0240	0.0241	-0.0885	0.0216	0.0221	-0.0923	0.0236	0.0237	-0.0926	0.0237	0.0238	-0.0870	0.0214	0.0219	0.0008	0.0005	0.0001
16	-0.0911	0.0355	0.0352	-0.0914	0.0356	0.0353	-0.0852	0.0313	0.0316	-0.0894	0.0345	0.0348	-0.0897	0.0345	0.0349	-0.0837	0.0309	0.0313	0.0011	0.0008	0.0008
17	-0.0854	0.0361	0.0354	-0.0857	0.0362	0.0356	-0.0796	0.0320	0.0319	-0.0837	0.0351	0.0351	-0.0840	0.0352	0.0352	-0.0781	0.0316	0.0316	0.0011	0.0008	0.0008
18	-0.0936	0.0353	0.0350	-0.0939	0.0354	0.0352	-0.0878	0.0310	0.0315	-0.0919	0.0342	0.0347	-0.0922	0.0343	0.0348	-0.0863	0.0307	0.0311	0.0011	0.0008	0.0008
19	-0.0951	0.0244	0.0242	-0.0955	0.0245	0.0243	-0.0932	0.0218	0.0223	-0.0943	0.0241	0.0238	-0.0947	0.0242	0.0239	-0.0926	0.0217	0.0221	0.0008	0.0006	0.0002
20	-0.0894	0.0246	0.0243	-0.0898	0.0247	0.0244	-0.0874	0.0220	0.0224	-0.0886	0.0243	0.0240	-0.0890	0.0244	0.0241	-0.0869	0.0219	0.0223	0.0008	0.0006	0.0002
21	-0.0975	0.0246	0.0243	-0.0979	0.0246	0.0244	-0.0958	0.0217	0.0222	-0.0967	0.0242	0.0239	-0.0971	0.0243	0.0240	-0.0952	0.0216	0.0220	0.0008	0.0006	0.0002
22	-0.0944	0.0335	0.0344	-0.0948	0.0336	0.0346	-0.0927	0.0308	0.0317	-0.0937	0.0328	0.0340	-0.0941	0.0330	0.0341	-0.0921	0.0306	0.0314	0.0011	0.0008	0.0003
23	-0.0889	0.0342	0.0346	-0.0892	0.0344	0.0347	-0.0870	0.0316	0.0320	-0.0881	0.0336	0.0342	-0.0885	0.0337	0.0343	-0.0864	0.0313	0.0317	0.0011	0.0008	0.0003
24	-0.0967	0.0335	0.0346	-0.0972	0.0336	0.0348	-0.0953	0.0304	0.0317	-0.0960	0.0329	0.0342	-0.0964	0.0329	0.0343	-0.0946	0.0301	0.0313	0.0011	0.0008	0.0003
25	-0.0949	0.0354	0.0330	-0.0961	0.0357	0.0333	-0.0921	0.0245	0.0252	-0.0933	0.0341	0.0321	-0.0944	0.0343	0.0324	-0.0909	0.0242	0.0248	0.0011	0.0008	0.0007
26	-0.0891	0.0353	0.0329	-0.0902	0.0356	0.0332	-0.0863	0.0246	0.0253	-0.0877	0.0338	0.0319	-0.0887	0.0341	0.0323	-0.0852	0.0243	0.0249	0.0011	0.0008	0.0007
27	-0.0973	0.0350	0.0335	-0.0986	0.0353	0.0338	-0.0947	0.0246	0.0252	-0.0958	0.0340	0.0325	-0.0970	0.0343	0.0328	-0.0935	0.0242	0.0248	0.0011	0.0008	0.0008
28	-0.0940	0.0515	0.0478	-0.0952	0.0519	0.0484	-0.0912	0.0353	0.0359	-0.0925	0.0515	0.0475	-0.0937	0.0519	0.0480	-0.0901	0.0348	0.0352	0.0016	0.0012	0.0015
29	-0.0880	0.0515	0.0475	-0.0891	0.0519	0.0481	-0.0853	0.0358	0.0361	-0.0866	0.0518	0.0473	-0.0876	0.0522	0.0479	-0.0842	0.0352	0.0354	0.0016	0.0012	0.0015
30	-0.0961	0.0539	0.0490	-0.0974	0.0543	0.0496	-0.0939	0.0351	0.0359	-0.0948	0.0532	0.0484	-0.0960	0.0535	0.0489	-0.0928	0.0347	0.0352	0.0018	0.0012	0.0016
31	-0.0939	0.0252	0.0257	-0.0942	0.0253	0.0258	-0.0908	0.0222	0.0230	-0.0927	0.0247	0.0253	-0.0930	0.0248	0.0254	-0.0899	0.0219	0.0227	0.0008	0.0006	0.0002
32	-0.0885	0.0254	0.0257	-0.0888	0.0255	0.0258	-0.0853	0.0224	0.0231	-0.0874	0.0249	0.0253	-0.0878	0.0250	0.0254	-0.0845	0.0222	0.0228	0.0008	0.0006	0.0002
33	-0.0960	0.0253	0.0257	-0.0964	0.0254	0.0258	-0.0931	0.0222	0.0230	-0.0948	0.0248	0.0254	-0.0951	0.0249	0.0254	-0.0921	0.0219	0.0227	0.0008	0.0006	0.0002
34	-0.0935	0.0384	0.0379	-0.0937	0.0385	0.0380	-0.0903	0.0320	0.0327	-0.0926	0.0373	0.0373	-0.0929	0.0374	0.0374	-0.0893	0.0316	0.0323	0.0012	0.0009	0.0009
35	-0.0882	0.0380	0.0378	-0.0884	0.0381	0.0379	-0.0848	0.0323	0.0330	-0.0872	0.0372	0.0374	-0.0874	0.0373	0.0375	-0.0838	0.0318	0.0325	0.0012	0.0009	0.0009
36	-0.0957	0.0388	0.0381	-0.0960	0.0389	0.0383	-0.0927	0.0321	0.0327	-0.0948	0.0373	0.0374	-0.0951	0.0374	0.0376	-0.0917	0.0315	0.0322	0.0012	0.0009	0.0009
37	-0.0623	0.0240	0.0243	-0.0629	0.0240	0.0244	-0.0591	0.0222	0.0229	-0.0610	0.0237	0.0240	-0.0615	0.0238	0.0241	-0.0580	0.0222	0.0227	0.0008	0.0005	0.0002
38	-0.0546	0.0241	0.0245	-0.0552	0.0242	0.0246	-0.0514	0.0224	0.0231	-0.0533	0.0239	0.0242	-0.0538	0.0239	0.0243	-0.0503	0.0224	0.0230	0.0008	0.0005	0.0002
39	-0.0657	0.0240	0.0242	-0.0662	0.0241	0.0243	-0.0625	0.0222	0.0228	-0.0643	0.0237	0.0239	-0.0649	0.0238	0.0240	-0.0614	0.0221	0.0226	0.0008	0.0005	0.0002
40	-0.0619	0.0347	0.0345	-0.0625	0.0348	0.0346	-0.0587	0.0325	0.0325	-0.0606	0.0342	0.0342	-0.0611	0.0343	0.0343	-0.0576	0.0323	0.0323	0.0011	0.0008	0.0003
41	-0.0543	0.0347	0.0348	-0.0548	0.0348	0.0349	-0.0511	0.0326	0.0329	-0.0530	0.0342	0.0346	-0.0535	0.0343	0.0347	-0.0500	0.0323	0.0327	0.0011	0.0008	0.0003
42	-0.0656	0.0342	0.0343	-0.0661	0.0343	0.0344	-0.0624	0.0321	0.0323	-0.0643	0.0338	0.0340	-0.0648	0.0339	0.0341	-0.0613	0.0319	0.0321	0.0011	0.0008	0.0003
43	-0.0616	0.0324	0.0307	-0.0632	0.0327	0.0310	-0.0567	0.0242	0.0249	-0.0589	0.0312	0.0299	-0.0604	0.0314	0.0302	-0.0546	0.0240	0.0246	0.0010	0.0007	0.0006
44	-0.0537	0.0311	0.0306	-0.0553	0.0314	0.0309	-0.0491	0.0243	0.0251	-0.0513	0.0311	0.0301	-0.0528	0.0313	0.0304	-0.0470	0.0241	0.0248	0.0010	0.0007	0.0005
45	-0.0649	0.0326	0.0307	-0.0665	0.0328	0.0310	-0.0600	0.0242	0.0248	-0.0622	0.0313	0.0299	-0.0638	0.0315	0.0302	-0.0580	0.0240	0.0245	0.0010	0.0007	0.0006
46	-0.0614	0.0475	0.0435	-0.0631	0.0479	0.0439	-0.0554	0.0358	0.0354	-0.0591	0.0473	0.0432	-0.0607	0.0476	0.0436	-0.0535	0.0354	0.0350	0.0015	0.0011	0.0011
47	-0.0536	0.0476	0.0438	-0.0554	0.0480	0.0442	-0.0476	0.0358	0.0358	-0.0513	0.0474	0.0435	-0.0530	0.0477	0.0439	-0.0457	0.0354	0.0353	0.0015	0.0011	0.0010
48	-0.0651	0.0471	0.0434	-0.0668	0.0475	0.0438	-0.0591	0.0355	0.0352	-0.0628	0.0469	0.0430	-0.0644	0.0472	0.0434	-0.0572	0.0352	0.0348	0.0015	0.0011	0.0011
49	-0.0596	0.0252	0.0252	-0.0600	0.0253	0.0252	-0.0540	0.0229	0.0233	-0.0578	0.0250	0.0249	-0.0581	0.0251	0.0249	-0.0525	0.0228	0.0231	0.0008	0.0006	0.0001
50	-0.0519	0.0255	0.0254	-0.0523	0.0255	0.0255	-0.0463	0.0232	0.0236	-0.0501	0.0253	0.0251	-0.0504	0.0254	0.0252	-0.0448	0.0231	0.0234	0.0008	0.0006	0.0001
51	-0.0630	0.0252	0.0251	-0.0633	0.0253	0.0252	-0.0574	0.0229	0.0233	-0.0611	0.0250	0.0248	-0.0614	0.0251	0.0249	-0.0559	0.0228	0.0230	0.0008	0.0006	0.0001
52	-0.0591	0.0374	0.0366	-0.0594	0.0375	0.0368	-0.0532	0.0334	0.0332	-0.0574	0.0364	0.0363	-0.0577	0.0365	0.0364	-0.0517	0.0330	0.0329	0.0012	0.0008	0.0008
53	-0.0515	0.0377	0.0370	-0.0518	0.0378	0.0371	-0.0455	0.0337	0.0336	-0.0498	0.0367	0.0367	-0.0500	0.0368	0.0368	-0.0440	0.0333	0.0333	0.0012	0.0009	0.0008
54	-0.0628	0.0371	0.0365	-0.0631	0.0372	0.0366	-0.0569	0.0332	0.0330	-0.0611	0.0360	0.0361	-0.0614	0.0361	0.0362	-0.0554	0.0328	0.0327	0.0012	0.0008	0.0008
55	-0.0631	0.0260	0.0257	-0.0634	0.0261	0.0258	-0.0618	0.0233	0.0236	-0.0625	0.0257	0.0253	-0.0628	0.0258	0.0254	-0.0614	0.0232	0.0234	0.0008	0.0006	0.0002
56	-0.0553	0.0259	0.0258	-0.0556	0.0260	0.0259	-0.0538	0.0235	0.0239	-0.0548	0.0256	0.0254	-0.0550	0.0257	0.0255	-0.0534	0.0234	0.0237	0.0008	0.0006	0.0002
57	-0.0666	0.0261	0.0259	-0.0669	0.0262	0.0260	-0.0655	0.0233	0.0236	-0.0660	0.0257	0.0255	-0.0663	0.0258	0.0256	-0.0650	0.0232	0.0234	0.0008	0.0006	0.0002
58	-0.0627	0.0367	0.0365	-0.0630	0.0368	0.0367	-0.0616	0.0337	0.0337	-0.0622	0.0360	0.0361	-0.0625	0.0361	0.0362	-0.0612	0.0333	0.0333	0.0012	0.0008	0.0003
59	-0.0546	0.0370	0.0368	-0.0549	0.0371	0.0369	-0.0534	0.0340	0.0340	-0.0542	0.0362	0.0363	-0.0544	0.0363	0.0365	-0.0530	0.0336	0.0337	0.0012	0.0008	0.0003
60	-0.0661	0.0366	0.0366	-0.0665	0.0368	0.0367	-0.0653	0.0335	0.0336	-0.0656	0.0359	0.0361	-0.0659	0.0360	0.0363	-0.0649	0.0332	0.0333	0.0012	0.0008	0.0003
61	-0.0629	0.0369	0.0352	-0.0637	0.0372	0.0356	-0.0611	0.0262	0.0267	-0.0618	0.0358	0.0342	-0.0626	0.0361	0.0345	-0.0603	0.0258	0.0263	0.0012	0.0008	0.0008
62	-0.0549	0.0372	0.0350	-0.0556	0.0375	0.0353	-0.0532	0.0263	0.0269	-0.0539	0.0356	0.0340	-0.0546	0.0359	0.0344	-0.0525	0.0259	0.0264	0.0012	0.0008	0.0007
63	-0.0671	0.0369	0.0357	-0.0682	0.0372	0.0361	-0.0654	0.0263	0.0269	-0.0660	0.0358	0.0346	-0.0670	0.0360	0.0349	-0.0645	0.0259	0.0264	0.0012	0.0008	0.0008
64	-0.0613	0.0560	0.0515	-0.0622	0.0565	0.0521	-0.0606	0.0377	0.0381	-0.0605	0.0551	0.0507	-0.0613	0.0554	0.0513	-0.0598	0.0372	0.0373	0.0018	0.0013	0.0014
65	-0.0535	0.0557	0.0511	-0.0542	0.0561	0.0517	-0.0523	0.0380	0.0383	-0.0526	0.0549	0.0504	-0.0533	0.0553	0.0510	-0.0516	0.0374	0.0376	0.0018	0.0013	0.0015
66	-0.0652	0.0565	0.0522	-0.0662	0.0569	0.0528	-0.0646	0.0377	0.0382	-0.0642	0.0554	0.0514	-0.0652	0.0557	0.0519	-0.0638	0.0372	0.0374	0.0018	0.0013	0.0014
67	-0.0626	0.0268	0.0271	-0.0629	0.0269	0.0272	-0.0605	0.0237	0.0244	-0.0618	0.0262	0.0267	-0.0620	0.0263	0.0268	-0.0598	0.0234	0.0241	0.0008	0.0006	0.0002
68	-0.0552	0.0269	0.0272	-0.0555	0.0270	0.0273	-0.0529	0.0239	0.0246	-0.0544	0.0264	0.0268	-0.0546	0.0265	0.0269	-0.0524	0.0236	0.0243	0.0009	0.0006	0.0002
69	-0.0656	0.0270	0.0274	-0.0659	0.0271	0.0275	-0.0637	0.0237	0.0244	-0.0646	0.0266	0.0270	-0.0649	0.0267	0.0271	-0.0630	0.0235	0.0241	0.0009	0.0006	0.0002
70	-0.0620	0.0409	0.0402	-0.0623	0.0410	0.0403	-0.0601	0.0343	0.0348	-0.0615	0.0396	0.0395	-0.0617	0.0397	0.0397	-0.0594	0.0337	0.0343	0.0013	0.0009	0.0008
71	-0.0548	0.0410	0.0403	-0.0550	0.0411	0.0404	-0.0527	0.0345	0.0351	-0.0542	0.0399	0.0397	-0.0544	0.0400	0.0398	-0.0520	0.0340	0.0346	0.0013	0.0009	0.0009
72	-0.0654	0.0412	0.0404	-0.0657	0.0413	0.0405	-0.0636	0.0342	0.0348	-0.0647	0.0398	0.0398	-0.0650	0.0399	0.0399	-0.0629	0.0336	0.0343	0.0013	0.0009	0.0008

Appendix C Nestedloop plots of performance measures for estimates by IPCW implementations with NUC

C.1 Summary of performance measures when outcome analysis model does covariate adjustment

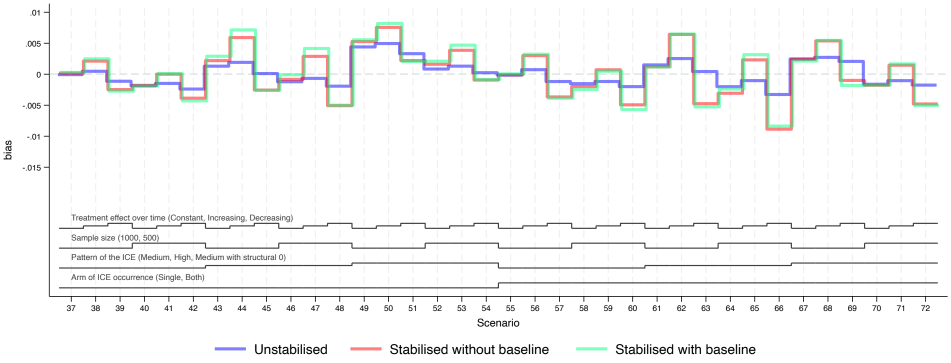

(a) Performance measure: bias. MCSE $\leq$ 0.002.

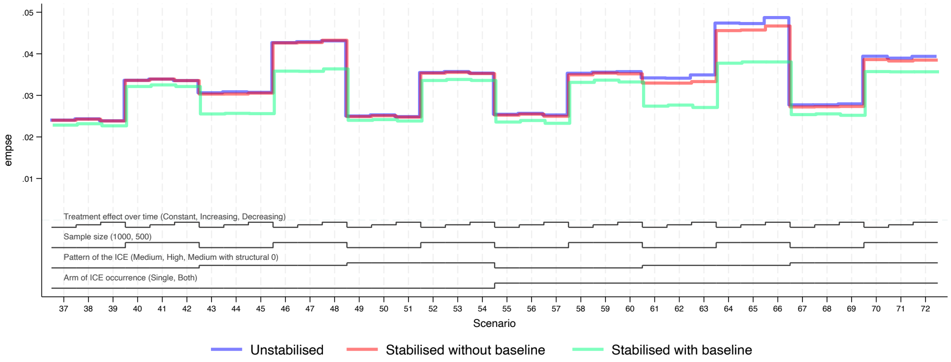

(b) Performance measure: EmpSE. MCSE $\leq$ 0.001.

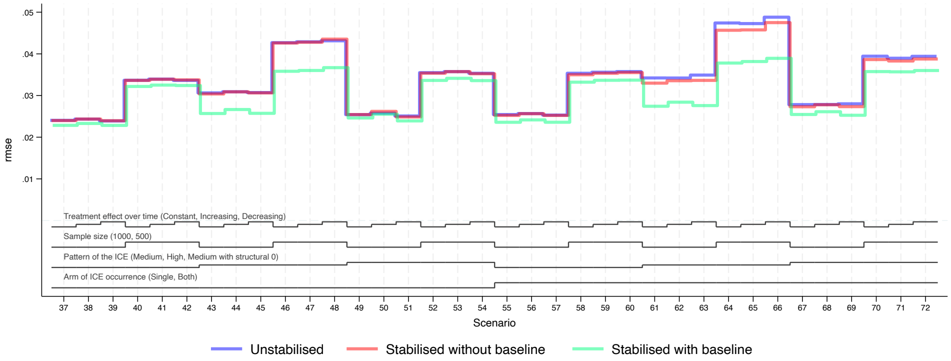

(c) Performance measure: RMSE. MCSE $\leq$ 0.001.

Figure C1. Performance measures of IPCW implementations in Scenario 37 to Scenario 72 when outcome analysis model does not account for time-varying treatment effect and does covariate adjustment.

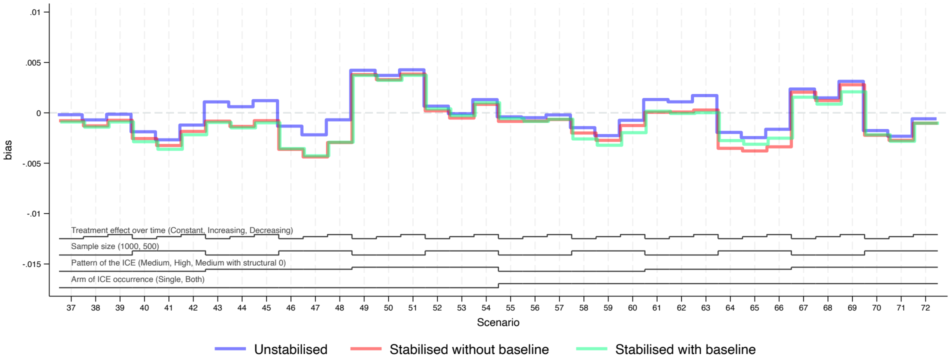

(a) Performance measure: bias. MCSE $\leq$ 0.002.

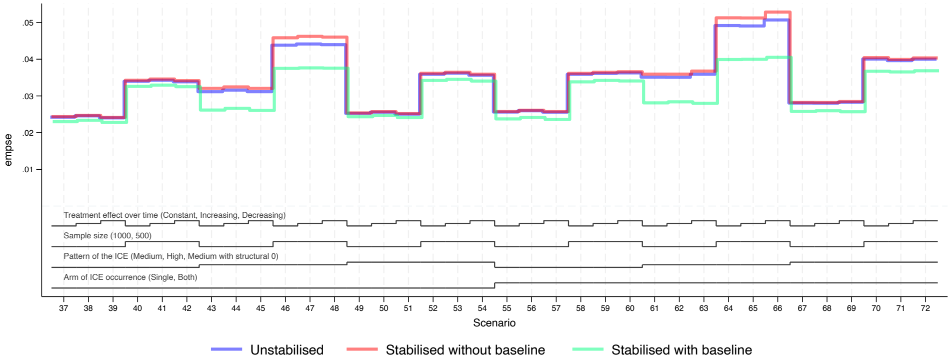

(b) Performance measure: EmpSE. MCSE $\leq$ 0.001.

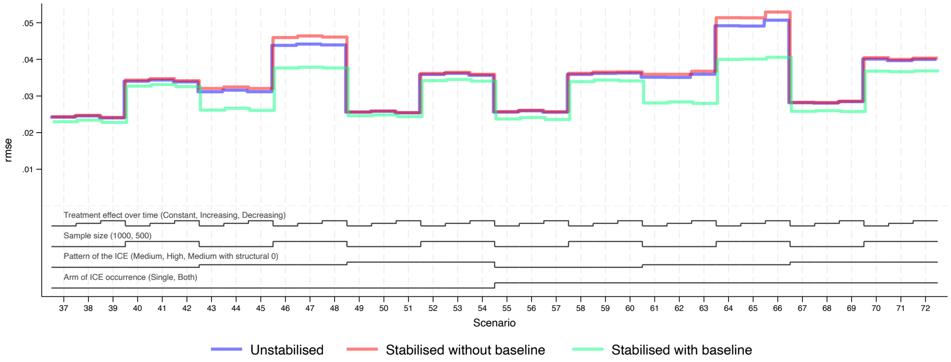

(c) Performance measure: RMSE. MCSE $\leq$ 0.001.

Figure C2. Performance measures of IPCW implementations in Scenario 37 to Scenario 72 when outcome analysis model accounts for time-varying treatment effect and does covariate adjustment.

C.2 Summary of performance measures when outcome analysis model does not do covariate adjustment

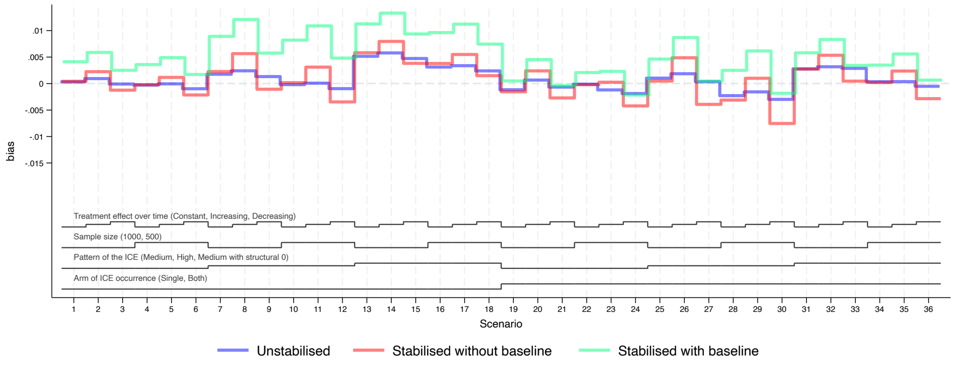

(a) Performance measure: bias. MCSE $\leq$ 0.002.

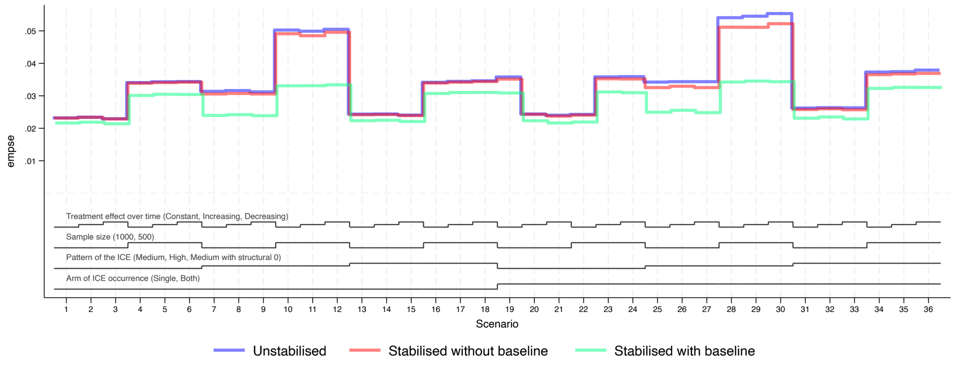

(b) Performance measure: EmpSE. MCSE $\leq$ 0.001.

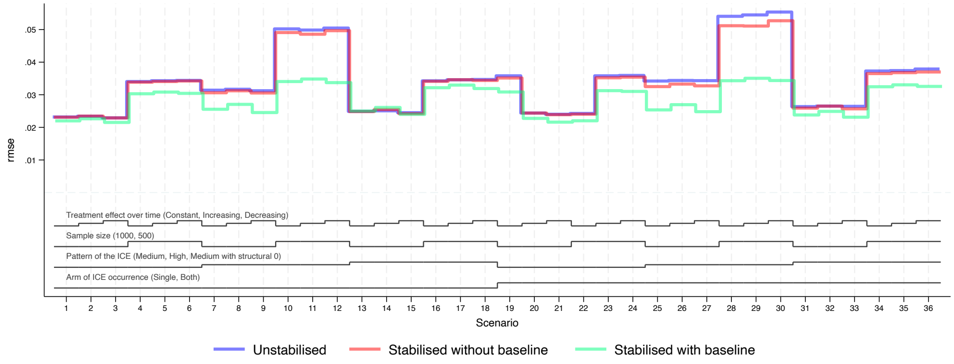

(c) Performance measure: RMSE. MCSE $\leq$ 0.001.

Figure C3. Performance measures of IPCW implementations in Scenario 1 to Scenario 36 when outcome analysis model does not account for time-varying treatment effect and does not do covariate adjustment.

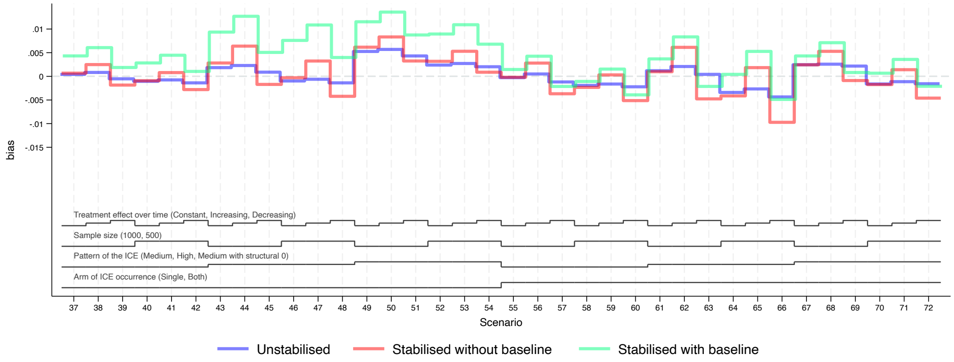

(a) Performance measure: bias. MCSE $\leq$ 0.002.

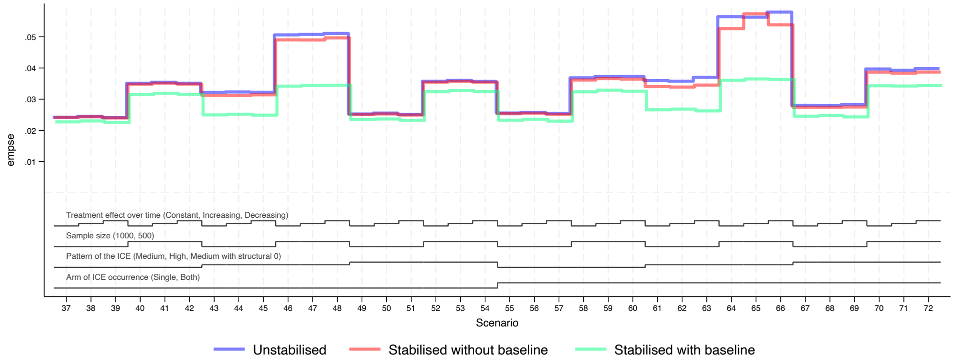

(b) Performance measure: EmpSE. MCSE $\leq$ 0.001.

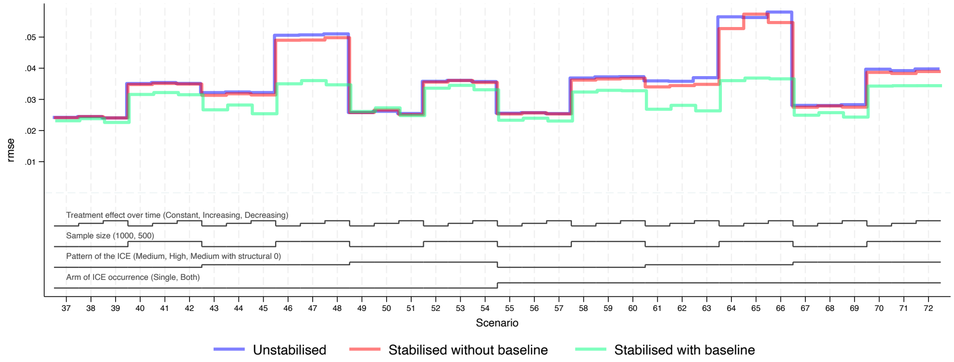

(c) Performance measure: RMSE. MCSE $\leq$ 0.001.

Figure C4. Performance measures of IPCW implementations in Scenario 37 to Scenario 72 when outcome analysis model does not account for time-varying treatment effect and does not do covariate adjustment.

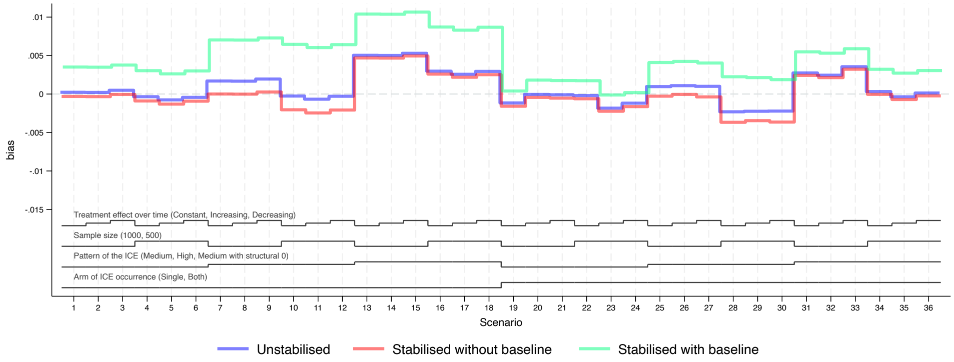

(a) Performance measure: bias. MCSE $\leq$ 0.002.

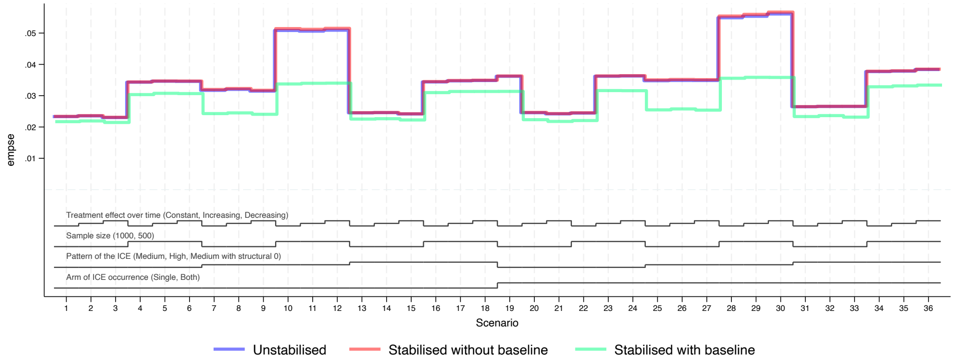

(b) Performance measure: EmpSE. MCSE $\leq$ 0.001.

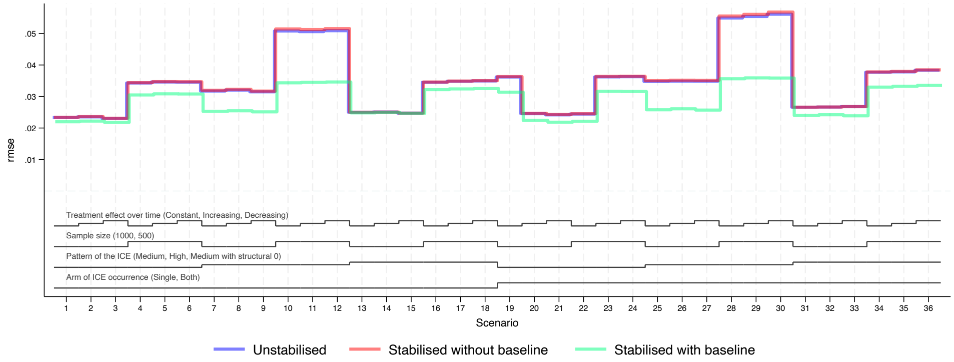

(c) Performance measure: RMSE. MCSE $\leq$ 0.001.

Figure C5. Performance measures of IPCW implementations in Scenario 1 to Scenario 36 when outcome analysis model accounts for time-varying treatment effect and does not do covariate adjustment.

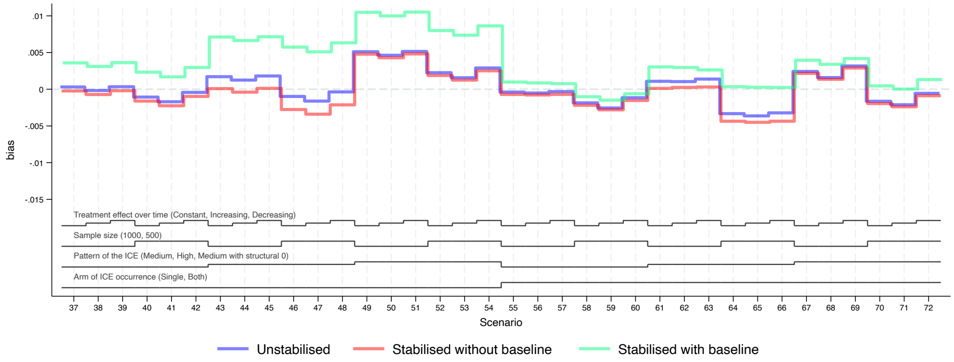

(a) Performance measure: bias. MCSE $\leq$ 0.002.

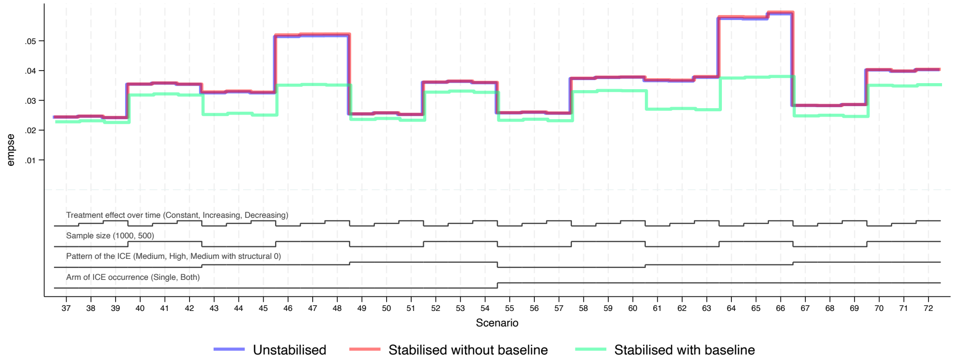

(b) Performance measure: EmpSE. MCSE $\leq$ 0.001.

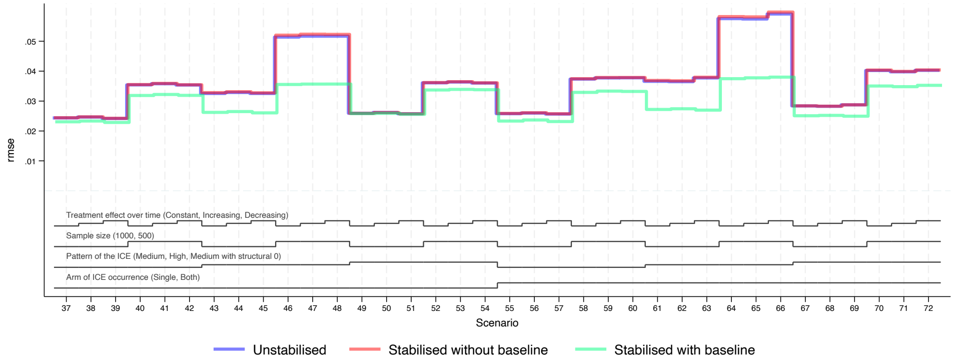

(c) Performance measure: RMSE. MCSE $\leq$ 0.001.

Figure C6. Performance measures of IPCW implementations in Scenario 37 to Scenario 72 when outcome analysis model accounts for time-varying treatment effect and does not do covariate adjustment.

Appendix D Nestedloop plots of performance measures for estimates by IPCW implementations with residual confounding (RC)

D.1 *Summary of performance measures when outcome analysis model does covariate adjustment*

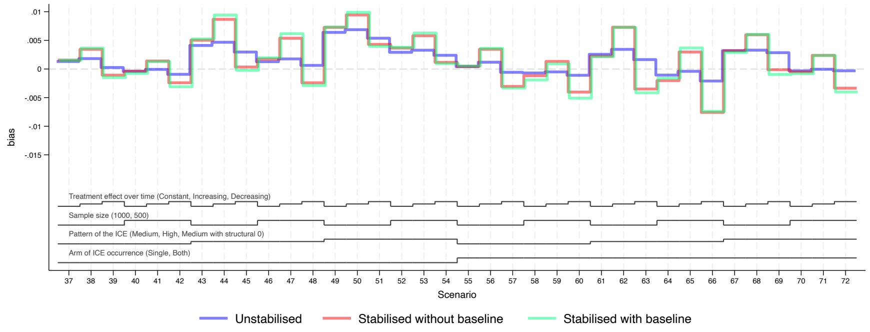

(a) Performance measure: bias. MCSE $\leq$ 0.002.

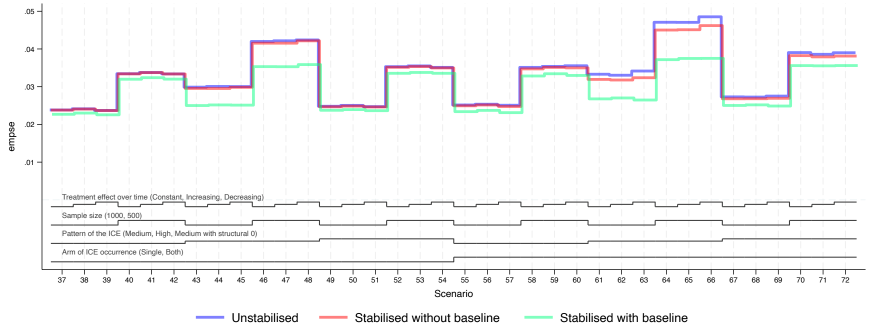

(b) Performance measure: EmpSE. MCSE $\leq$ 0.001.

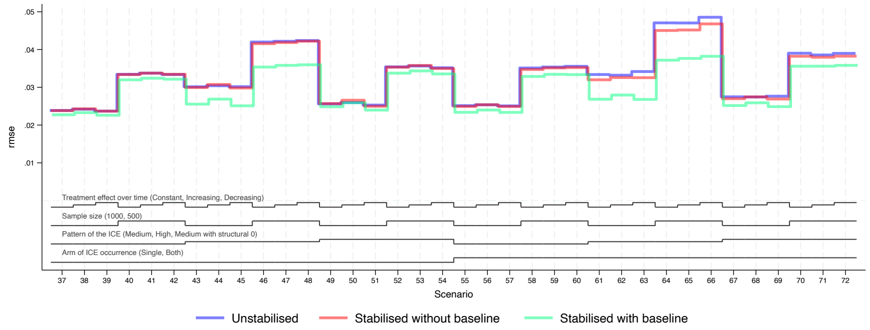

(c) Performance measure: RMSE. MCSE $\leq$ 0.001.

Figure D1. Performance measures of IPCW implementations in Scenario 37 to Scenario 72 when outcome analysis model does not account for time-varying treatment effect and does covariate adjustment.

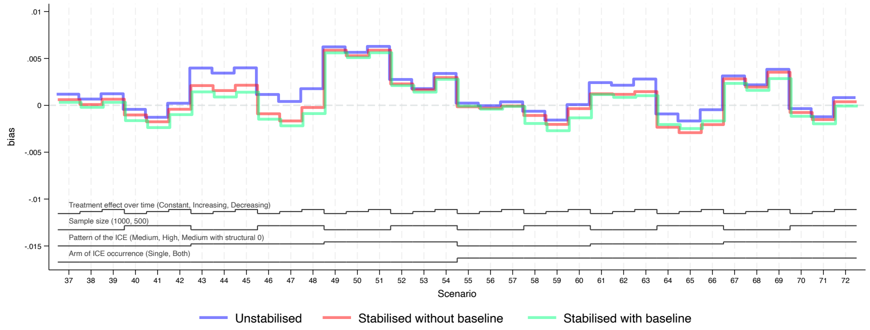

(a) Performance measure: bias. MCSE $\leq$ 0.002.

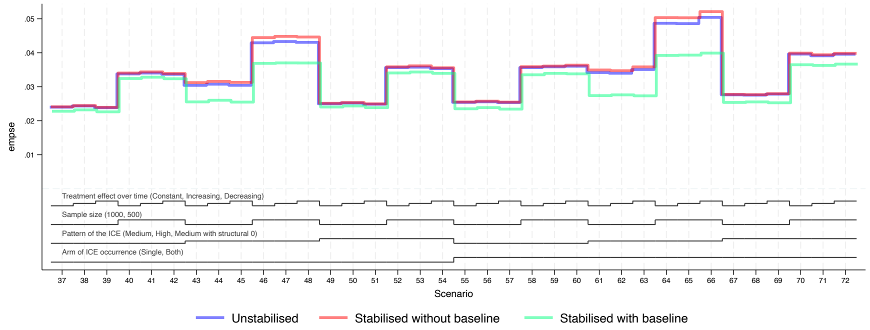

(b) Performance measure: EmpSE. MCSE $\leq$ 0.001.

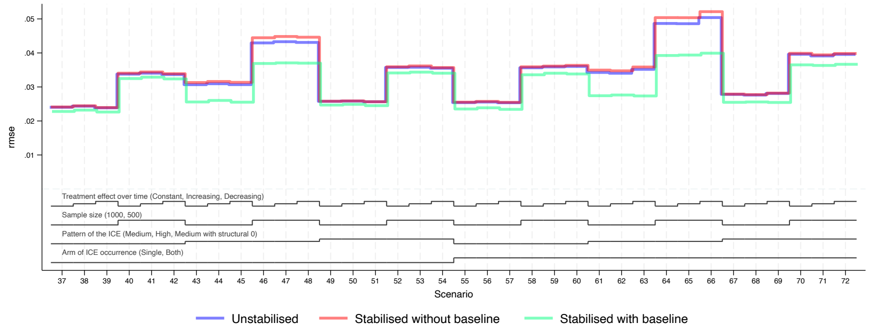

(c) Performance measure: RMSE. MCSE $\leq$ 0.001.

Figure D2. Performance measures of IPCW implementations in Scenario 37 to Scenario 72 when outcome analysis model accounts for time-varying treatment effect and does covariate adjustment.

D.2 Summary of performance measures when outcome analysis model does not do covariate adjustment

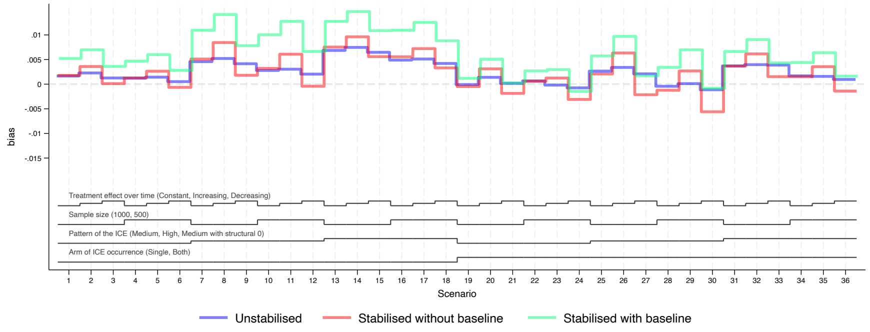

(a) Performance measure: bias. MCSE $\leq$ 0.002.

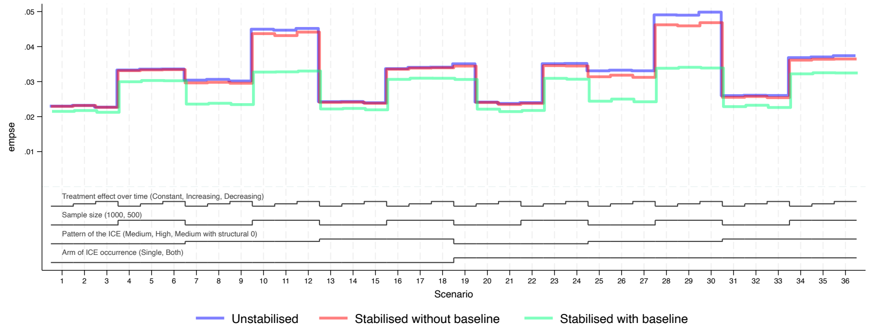

(b) Performance measure: EmpSE. MCSE $\leq$ 0.001.

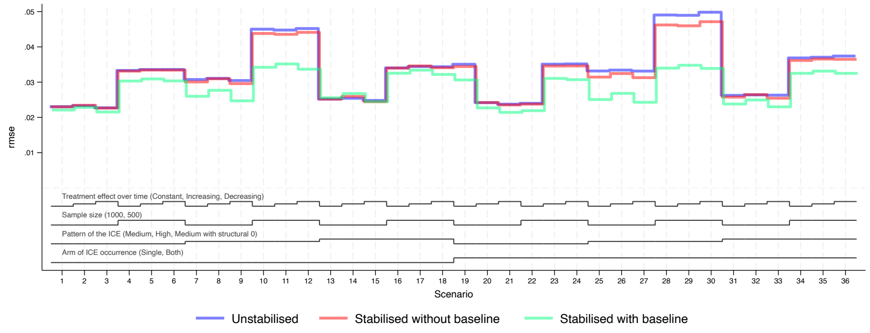

(c) Performance measure: RMSE. MCSE $\leq$ 0.001.

Figure D3. Performance measures of IPCW implementations in Scenario 1 to Scenario 36 when outcome analysis model does not account for time-varying treatment effect and does not do covariate adjustment.

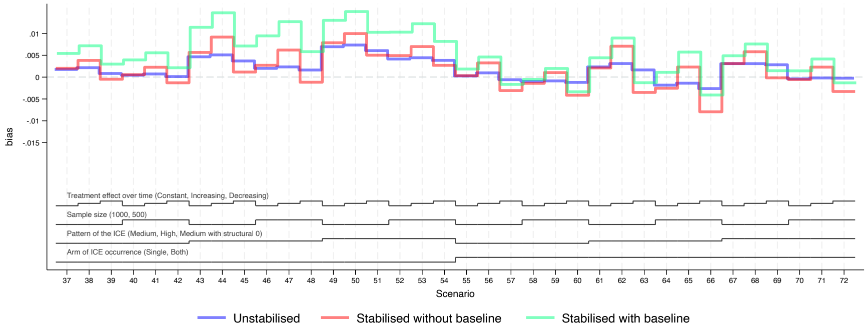

(a) Performance measure: bias. MCSE $\leq$ 0.002.

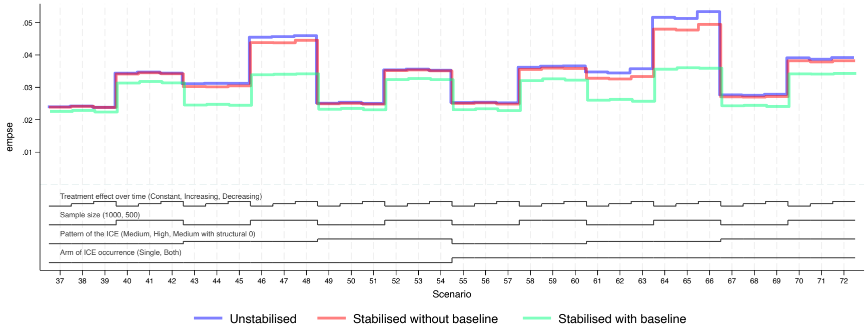

(b) Performance measure: EmpSE. MCSE $\leq$ 0.001.

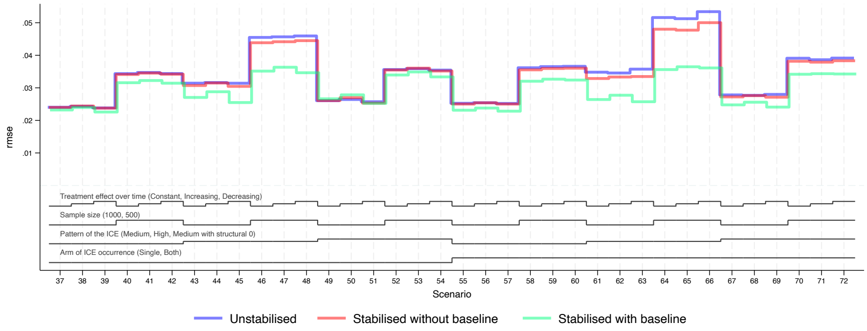

(c) Performance measure: RMSE. MCSE $\leq$ 0.001.

Figure D4. Performance measures of IPCW implementations in Scenario 37 to Scenario 72 when outcome analysis model does not account for time-varying treatment effect and does not do covariate adjustment.

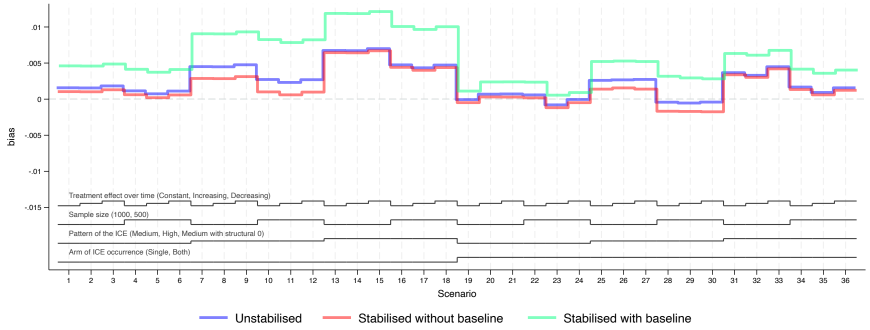

(a) Performance measure: bias. MCSE $\leq$ 0.002.

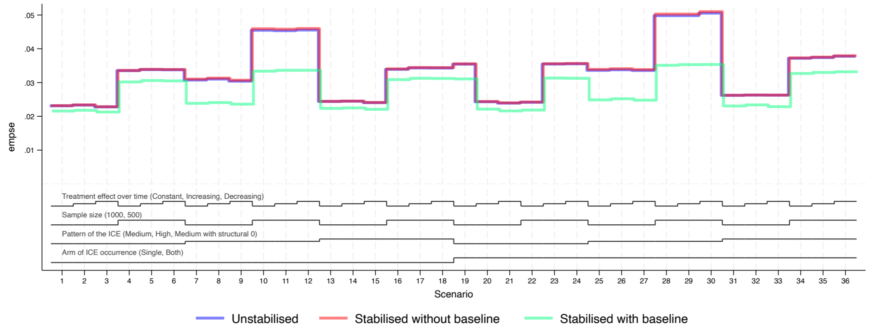

(b) Performance measure: EmpSE. MCSE $\leq$ 0.001.

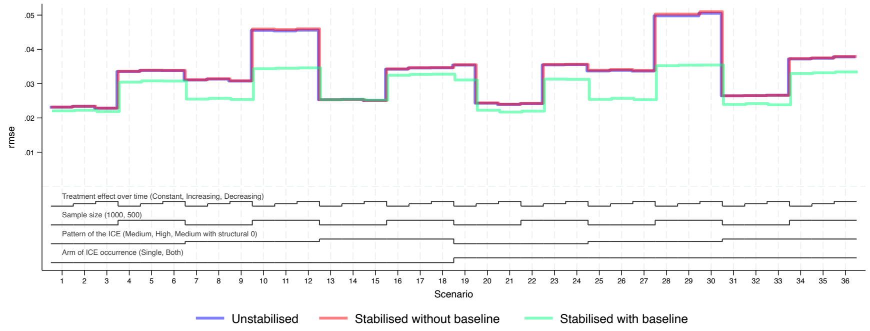

(c) Performance measure: RMSE. MCSE $\leq$ 0.001.

Figure D5. Performance measures of IPCW implementations in Scenario 1 to Scenario 36 when outcome analysis model accounts for time-varying treatment effect and does not do covariate adjustment.

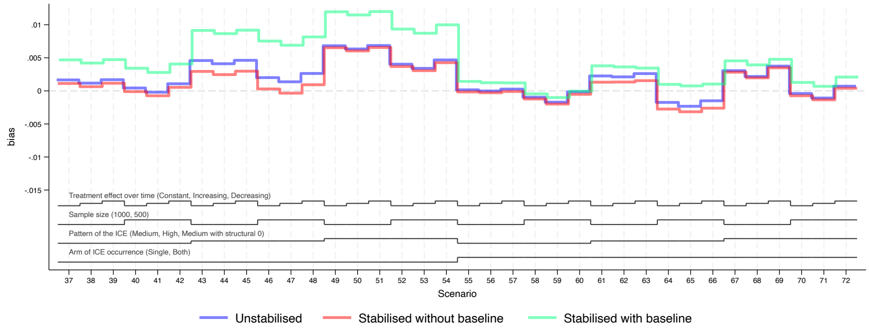

(a) Performance measure: bias. MCSE $\leq$ 0.002.

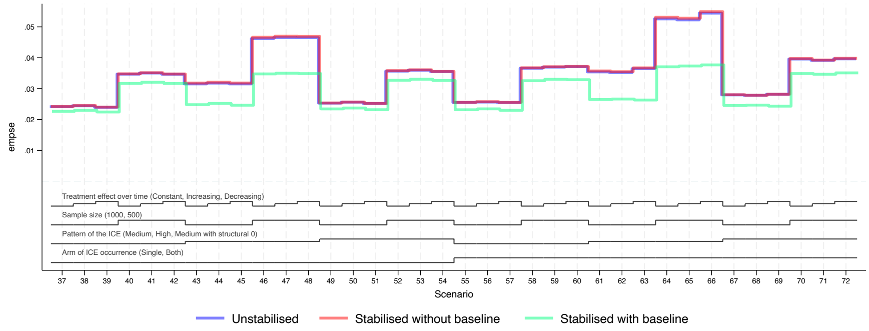

(b) Performance measure: EmpSE. MCSE $\leq$ 0.001.

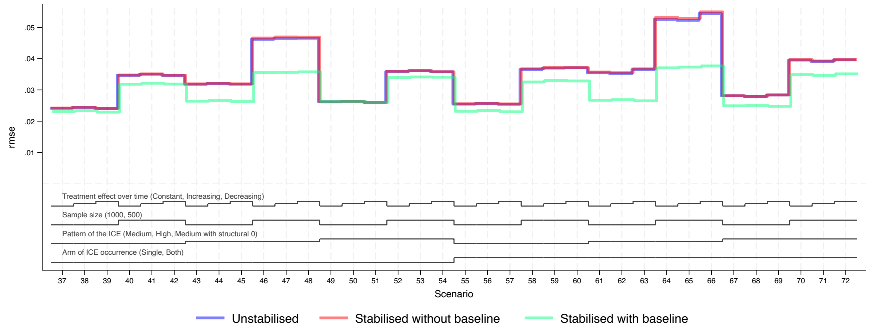

(c) Performance measure: RMSE. MCSE $\leq$ 0.001.

Figure D6. Performance measures of IPCW implementations in Scenario 37 to Scenario 72 when outcome analysis model accounts for time-varying treatment effect and does not do covariate adjustment.

Appendix E Nestedloop plots of performance measures for scenarios 73-144 with smaller baseline effect

E.1 Summary of performance measures for estimates by IPCW implementations with NUC

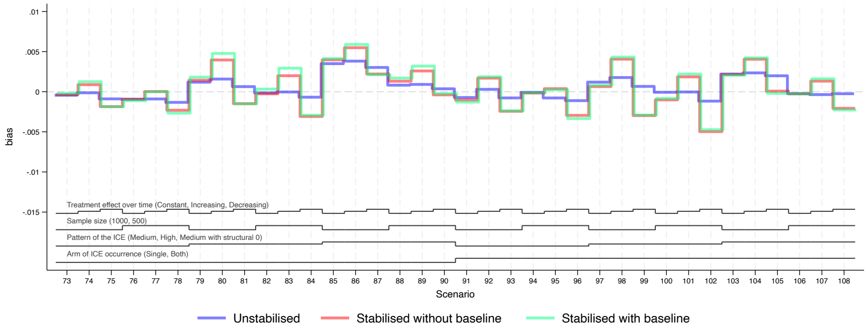

(a) Performance measure: bias. MCSE $\leq$ 0.002.

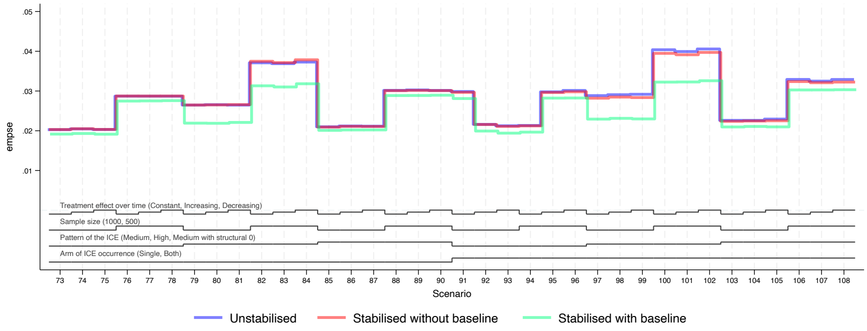

(b) Performance measure: EmpSE. MCSE $\leq$ 0.001.

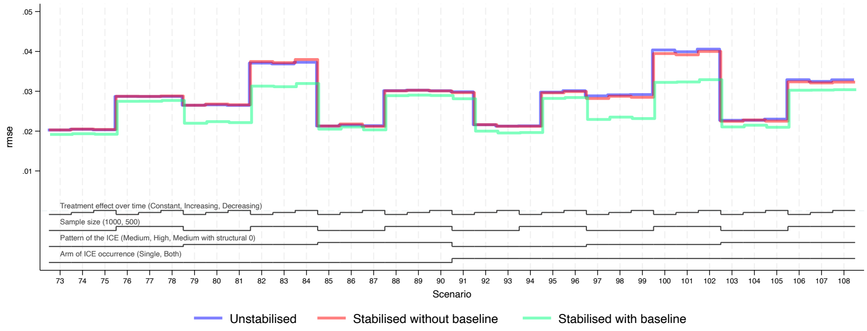

(c) Performance measure: RMSE. MCSE $\leq$ 0.001.

Figure E1. Performance measures of IPCW implementations with NUC in Scenario 73 to Scenario 108 when outcome analysis model does not account for time-varying treatment effect and does covariate adjustment.

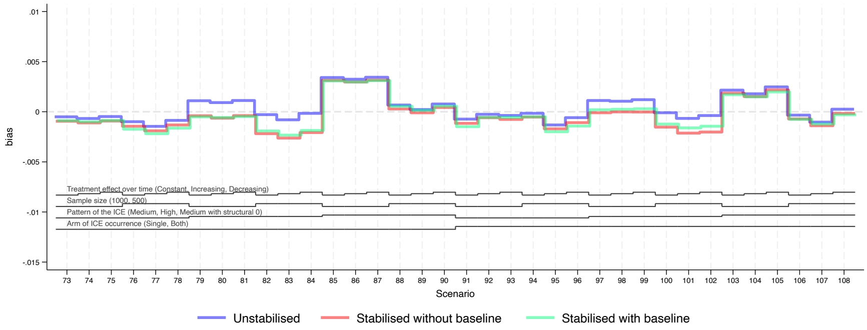

(a) Performance measure: bias. MCSE $\leq$ 0.002.

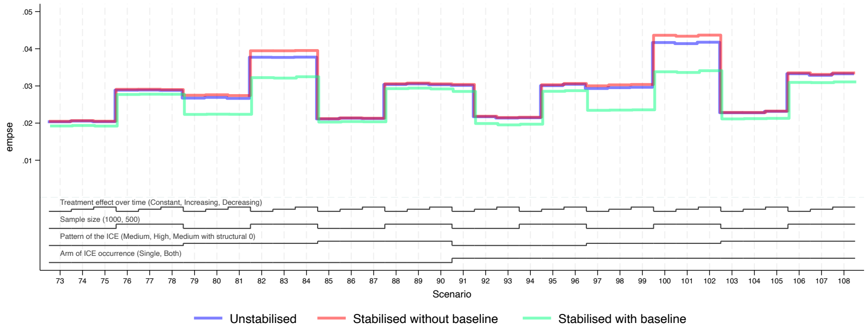

(b) Performance measure: EmpSE. MCSE $\leq$ 0.001.

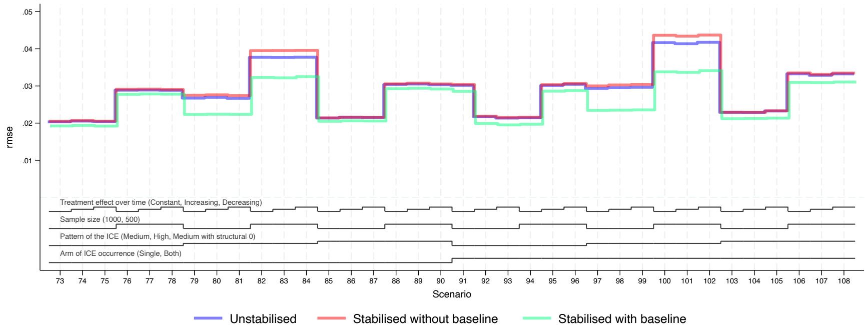

(c) Performance measure: RMSE. MCSE $\leq$ 0.001.

Figure E2. Performance measures of IPCW implementations with NUC in Scenario 73 to Scenario 108 when outcome analysis model accounts for time-varying treatment effect and does covariate adjustment.

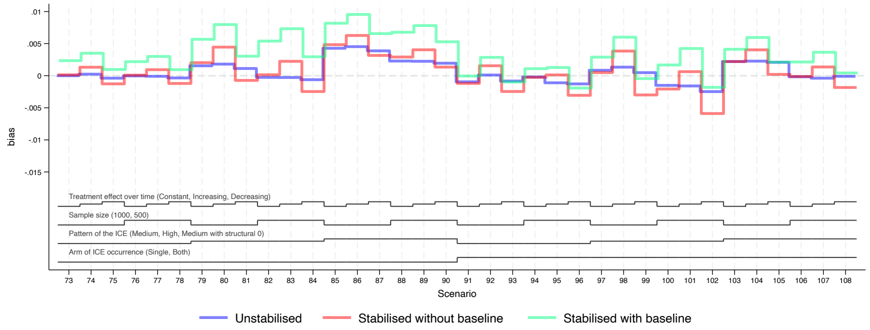

(a) Performance measure: bias. MCSE $\leq$ 0.002.

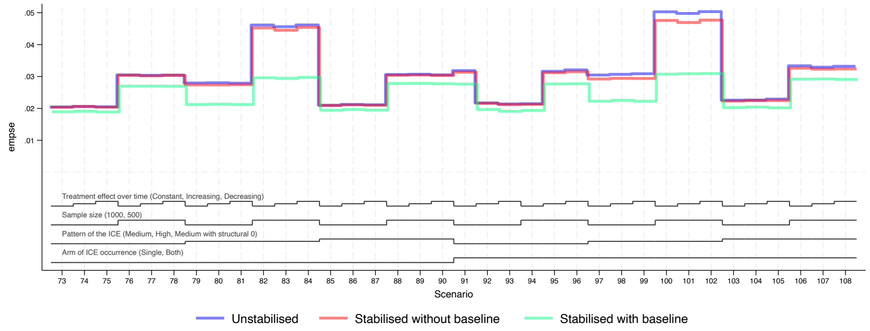

(b) Performance measure: EmpSE. MCSE $\leq$ 0.001.

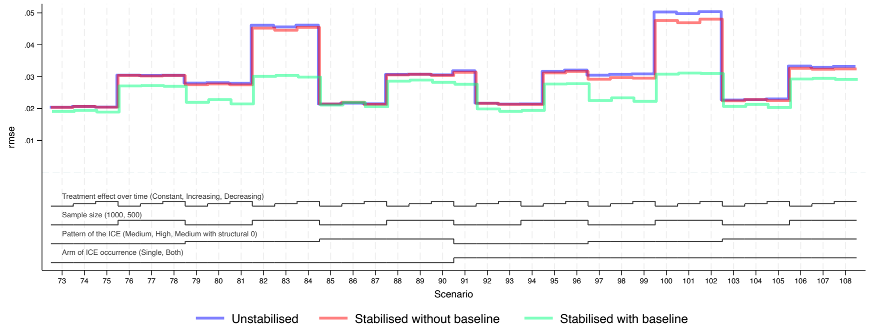

(c) Performance measure: RMSE. MCSE $\leq$ 0.001.

Figure E3. Performance measures of IPCW implementations with NUC in Scenario 73 to Scenario 108 when outcome analysis model does not account for time-varying treatment effect and does not do covariate adjustment.

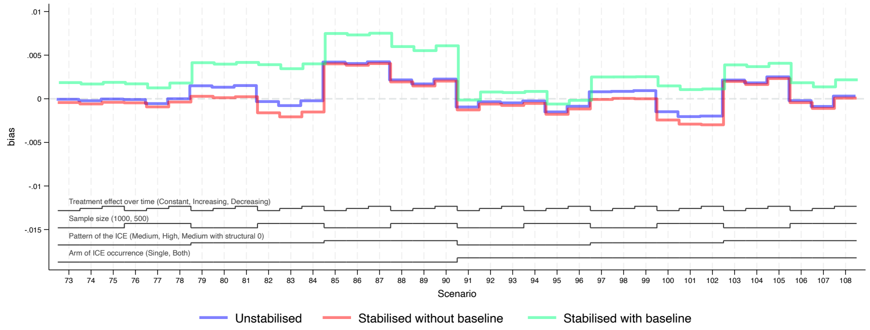

(a) Performance measure: bias. MCSE $\leq$ 0.002.

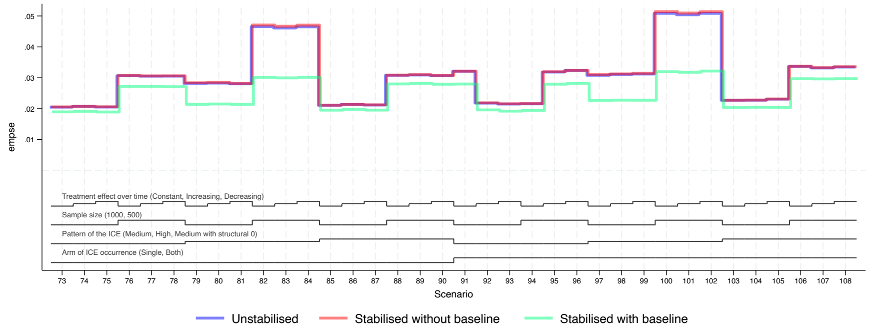

(b) Performance measure: EmpSE. MCSE $\leq$ 0.001.

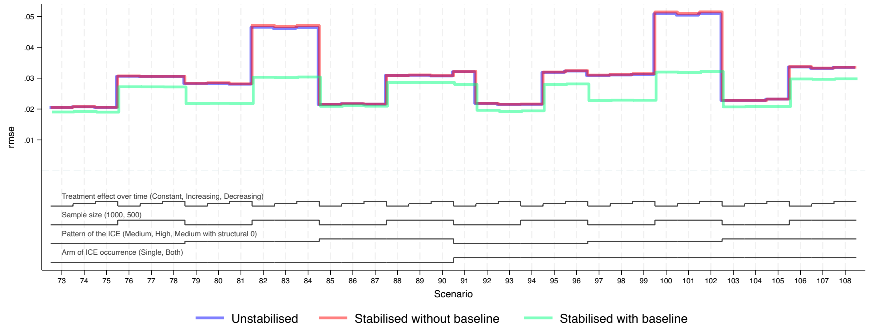

(c) Performance measure: RMSE. MCSE $\leq$ 0.001.

Figure E4. Performance measures of IPCW implementations with NUC in Scenario 73 to Scenario 108 when outcome analysis model does not account for time-varying treatment effect and does not do covariate adjustment.

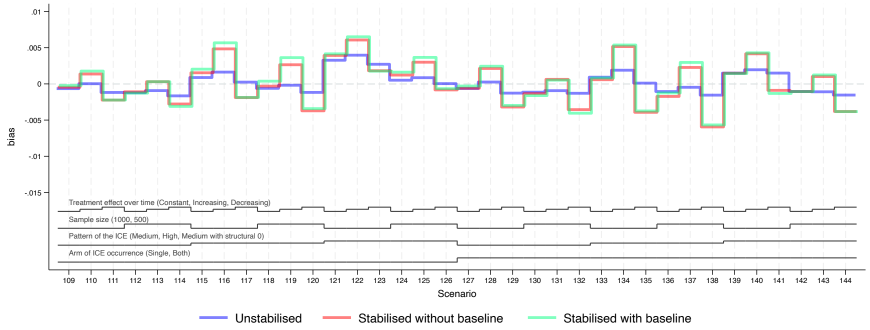

(a) Performance measure: bias. MCSE $\leq$ 0.002.

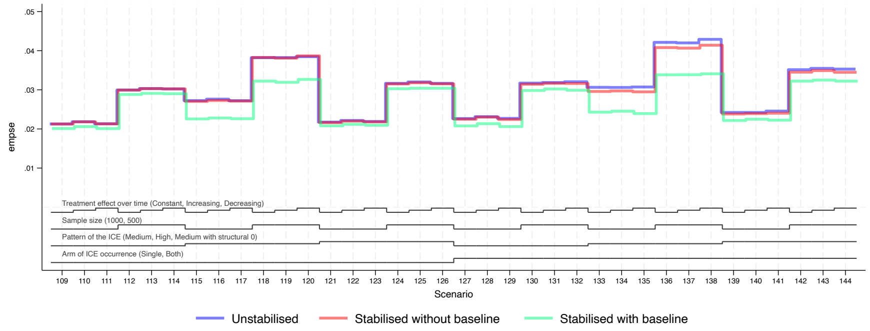

(b) Performance measure: EmpSE. MCSE $\leq$ 0.001.

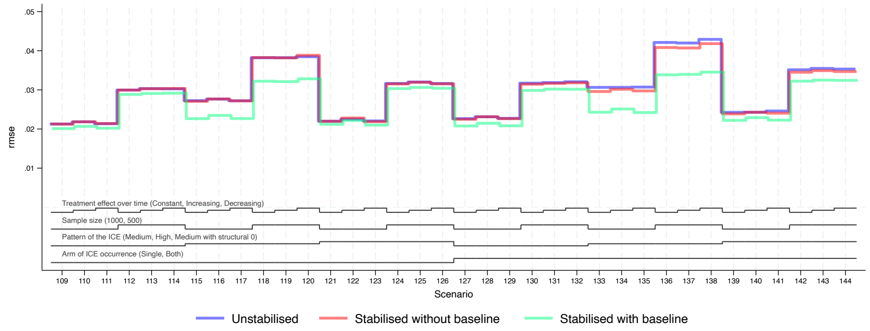

(c) Performance measure: RMSE. MCSE $\leq$ 0.001.

Figure E5. Performance measures of IPCW implementations with NUC in Scenario 109 to Scenario 144 when outcome analysis model does not account for time-varying treatment effect and does covariate adjustment.

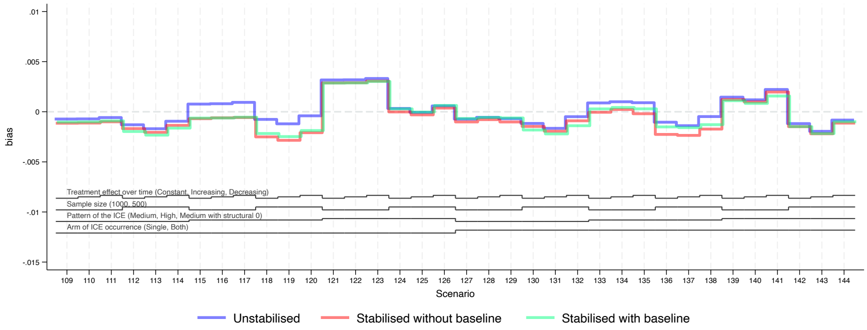

(a) Performance measure: bias. MCSE $\leq$ 0.002.

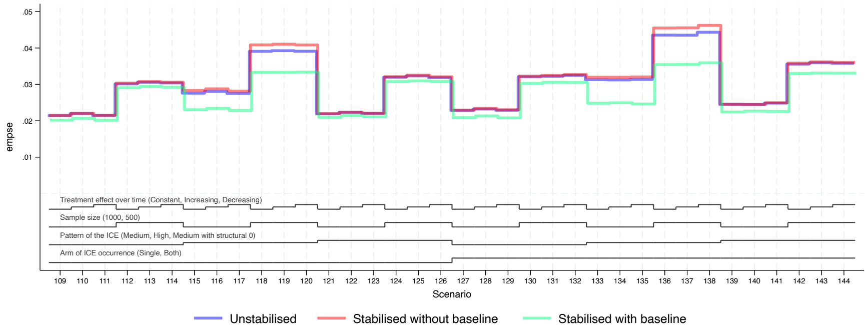

(b) Performance measure: EmpSE. MCSE $\leq$ 0.001.

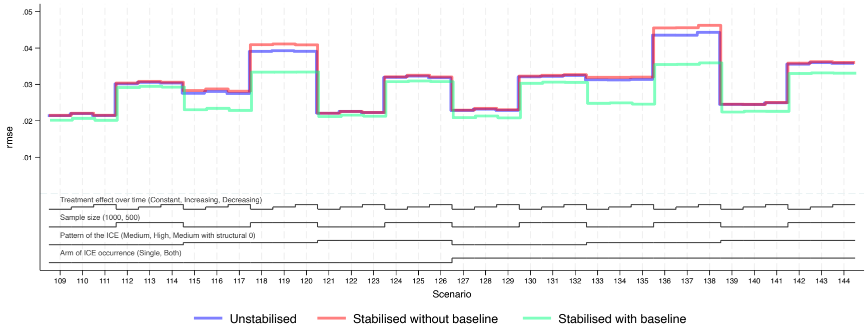

(c) Performance measure: RMSE. MCSE $\leq$ 0.001.

Figure E6. Performance measures of IPCW implementations with NUC in Scenario 109 to Scenario 144 when outcome analysis model accounts for time-varying treatment effect and does covariate adjustment.

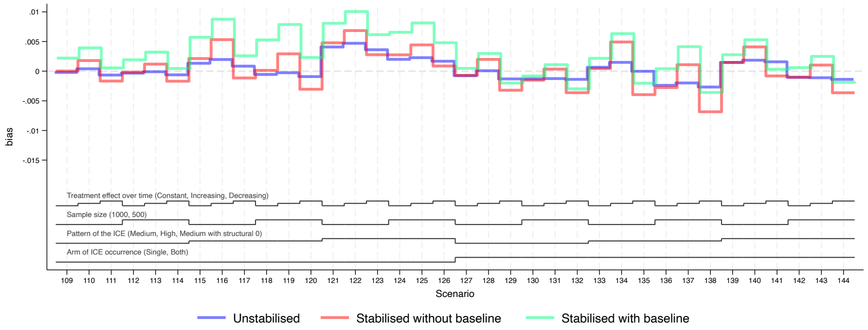

(a) Performance measure: bias. MCSE $\leq$ 0.002.

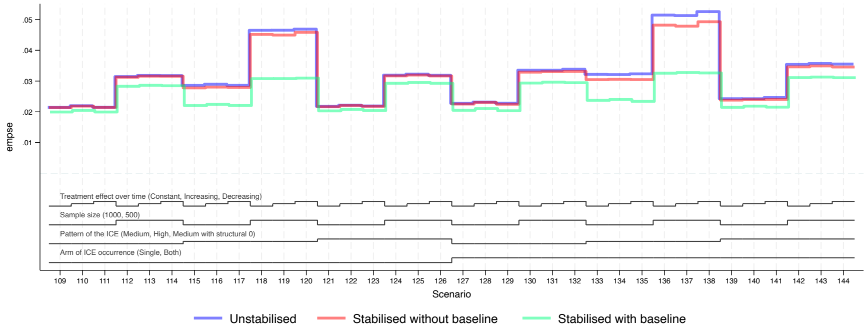

(b) Performance measure: EmpSE. MCSE $\leq$ 0.001.

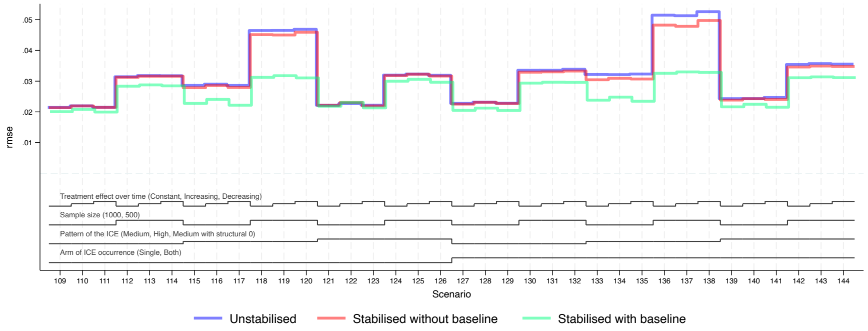

(c) Performance measure: RMSE. MCSE $\leq$ 0.001.

Figure E7. Performance measures of IPCW implementations with NUC in Scenario 109 to Scenario 144 when outcome analysis model does not account for time-varying treatment effect and does not do covariate adjustment.

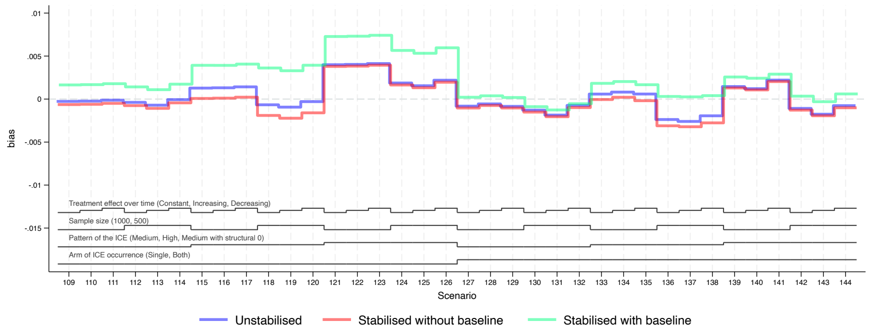

(a) Performance measure: bias. MCSE $\leq$ 0.002.

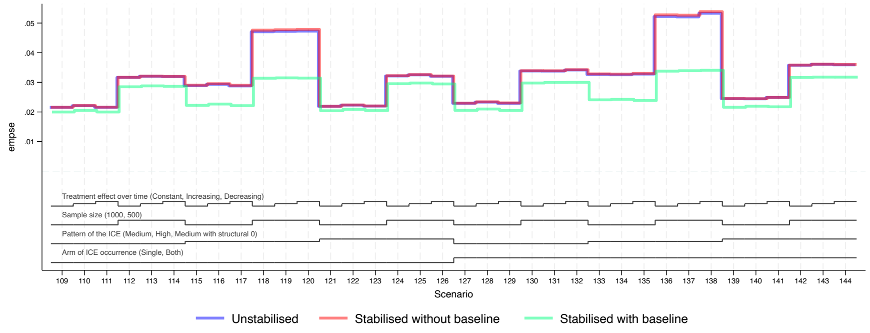

(b) Performance measure: EmpSE. MCSE $\leq$ 0.001.

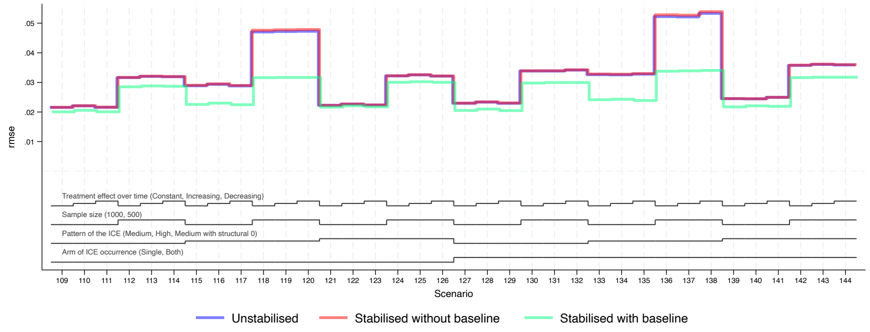

(c) Performance measure: RMSE. MCSE $\leq$ 0.001.

Figure E8. Performance measures of IPCW implementations with NUC in Scenario 109 to Scenario 144 when outcome analysis model does not account for time-varying treatment effect and does not do covariate adjustment.

E.2 Summary of performance measures for estimates by IPCW implementations with RC

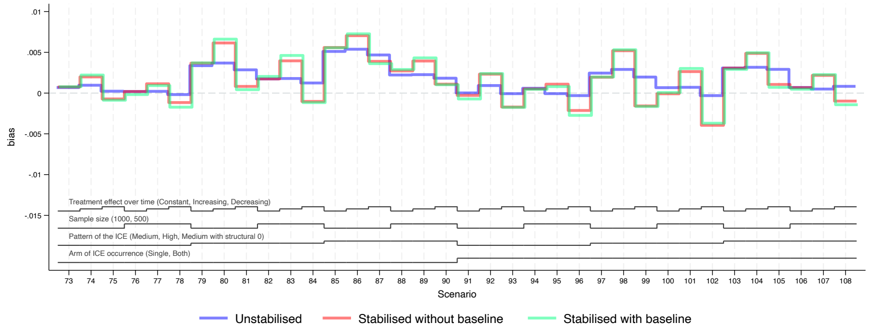

(a) Performance measure: bias. MCSE $\leq$ 0.002.

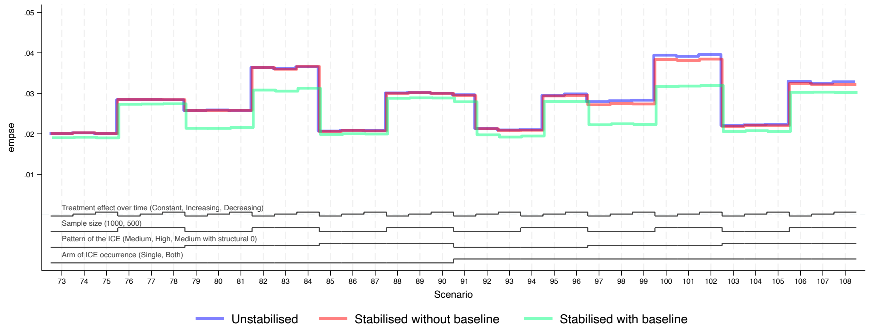

(b) Performance measure: EmpSE. MCSE $\leq$ 0.001.

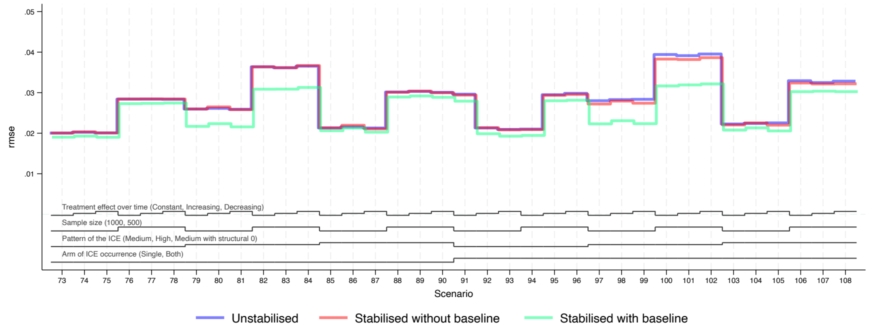

(c) Performance measure: RMSE. MCSE $\leq$ 0.001.

Figure E9. Performance measures of IPCW implementations with RC in Scenario 73 to Scenario 108 when outcome analysis model does not account for time-varying treatment effect and does covariate adjustment.

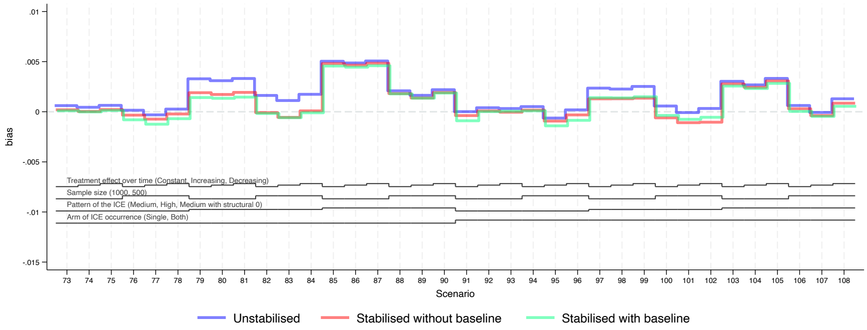

(a) Performance measure: bias. MCSE $\leq$ 0.002.

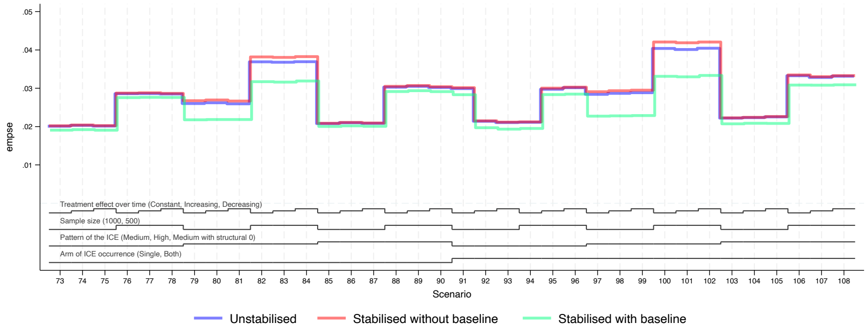

(b) Performance measure: EmpSE. MCSE $\leq$ 0.001.

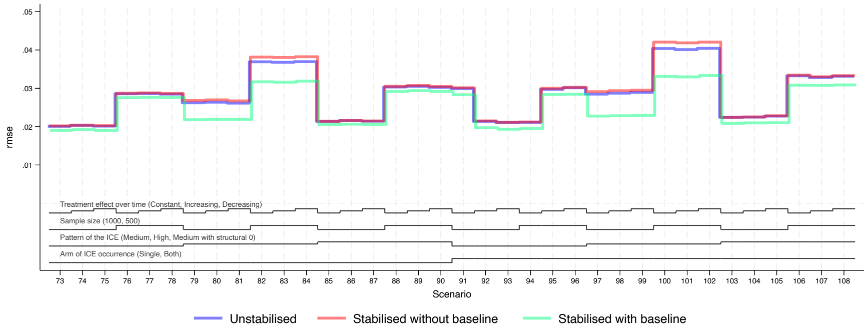

(c) Performance measure: RMSE. MCSE $\leq$ 0.001.

Figure E10. Performance measures of IPCW implementations with RC in Scenario 73 to Scenario 108 when outcome analysis model accounts for time-varying treatment effect and does covariate adjustment.

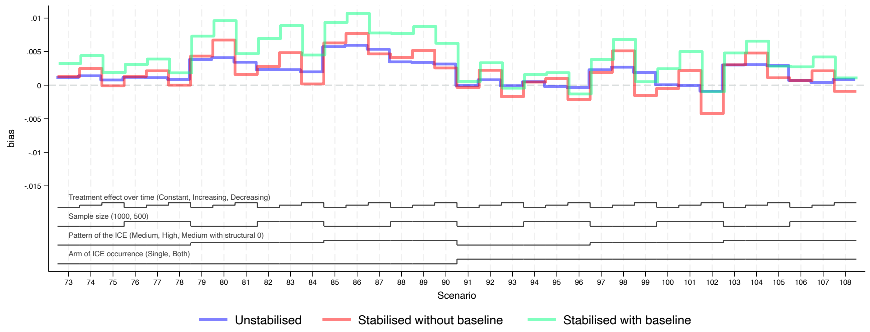

(a) Performance measure: bias. MCSE $\leq$ 0.002.

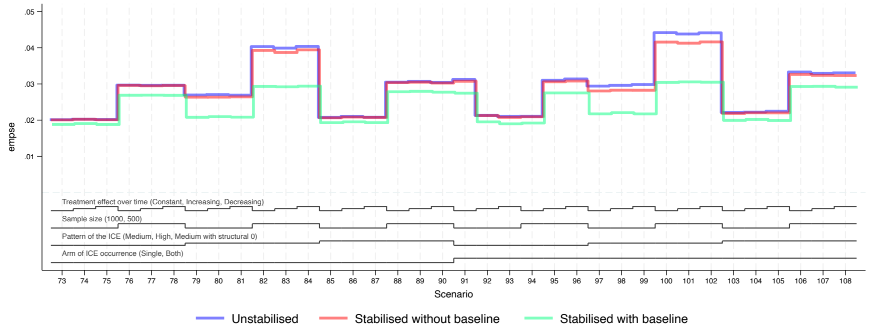

(b) Performance measure: EmpSE. MCSE $\leq$ 0.001.

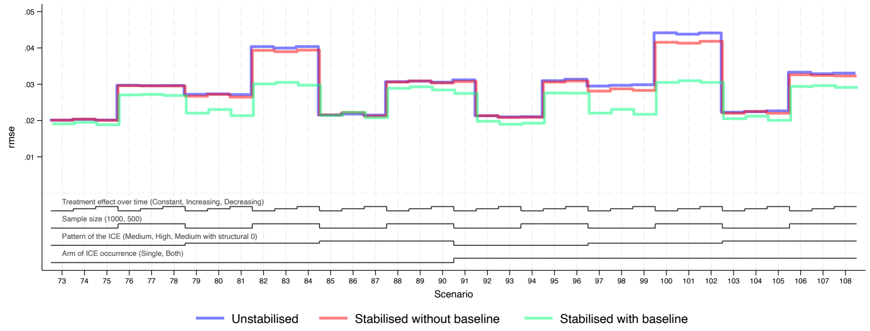

(c) Performance measure: RMSE. MCSE $\leq$ 0.001.

Figure E11. Performance measures of IPCW implementations with RC in Scenario 73 to Scenario 108 when outcome analysis model does not account for time-varying treatment effect and does not do covariate adjustment.

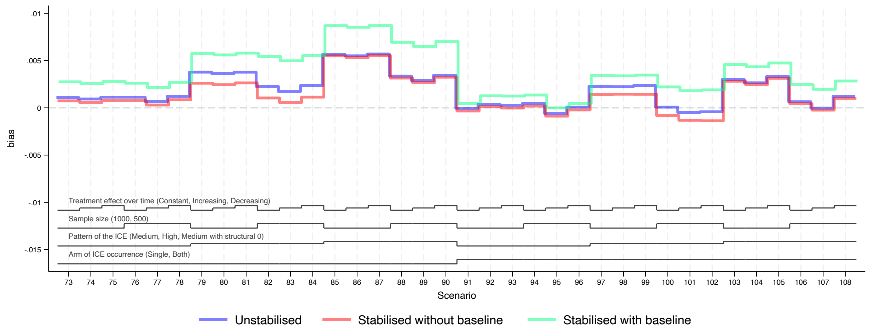

(a) Performance measure: bias. MCSE $\leq$ 0.002.

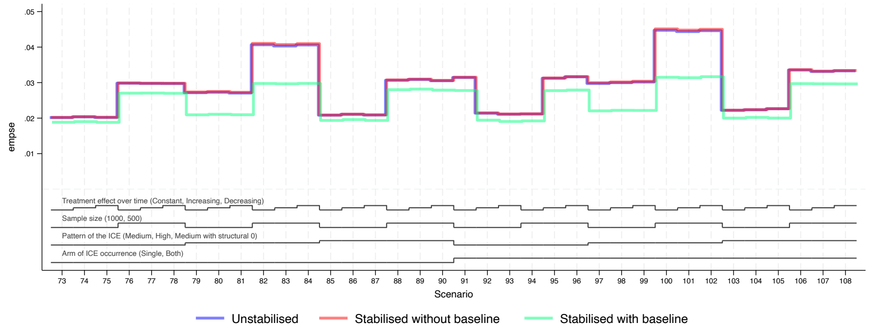

(b) Performance measure: EmpSE. MCSE $\leq$ 0.001.

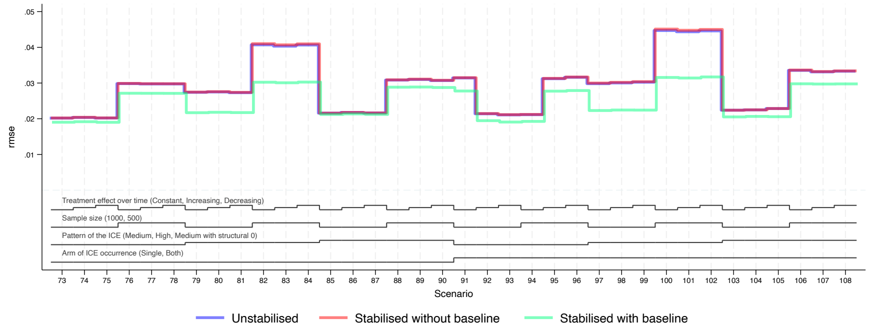

(c) Performance measure: RMSE. MCSE $\leq$ 0.001.

Figure E12. Performance measures of IPCW implementations with RC in Scenario 73 to Scenario 108 when outcome analysis model does not account for time-varying treatment effect and does not do covariate adjustment.

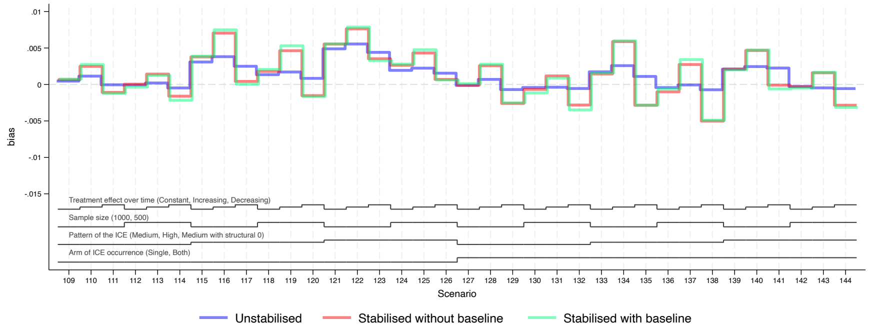

(a) Performance measure: bias. MCSE $\leq$ 0.002.

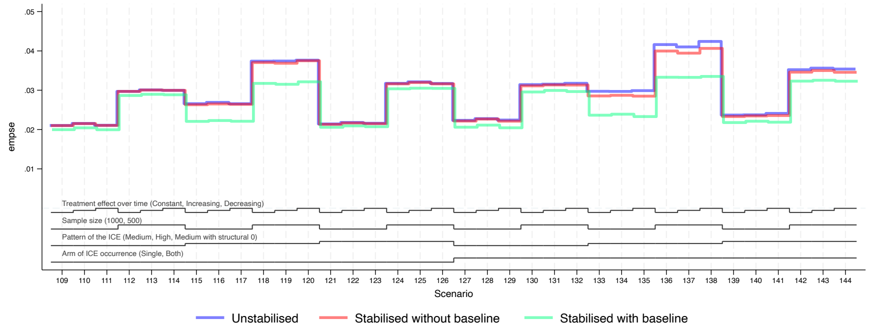

(b) Performance measure: EmpSE. MCSE $\leq$ 0.001.

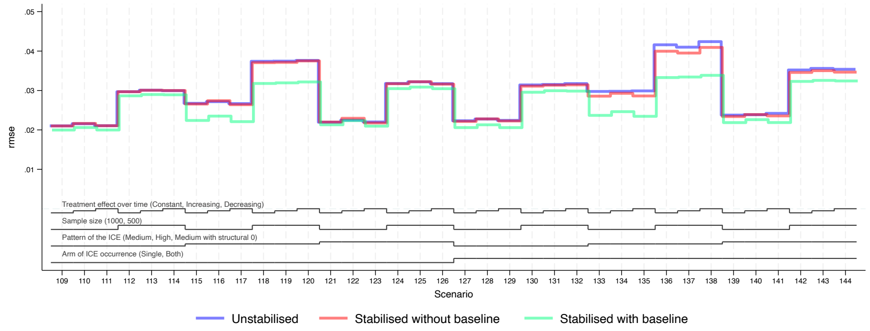

(c) Performance measure: RMSE. MCSE $\leq$ 0.001.

Figure E13. Performance measures of IPCW implementations with RC in Scenario 109 to Scenario 144 when outcome analysis model does not account for time-varying treatment effect and does covariate adjustment.

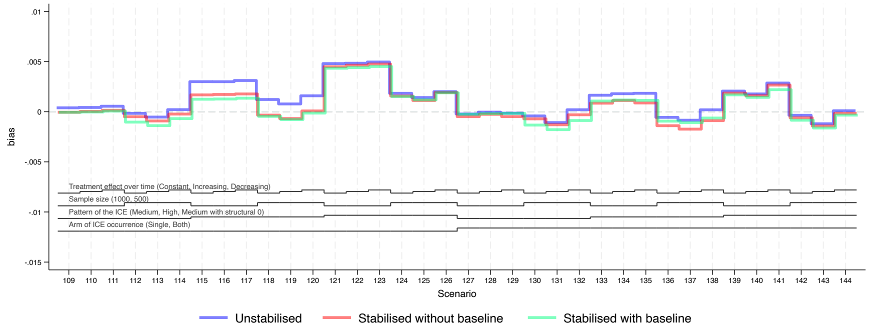

(a) Performance measure: bias. MCSE $\leq$ 0.002.

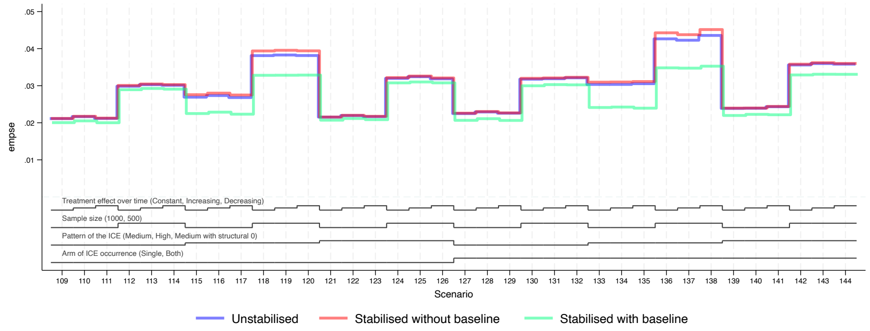

(b) Performance measure: EmpSE. MCSE $\leq$ 0.001.

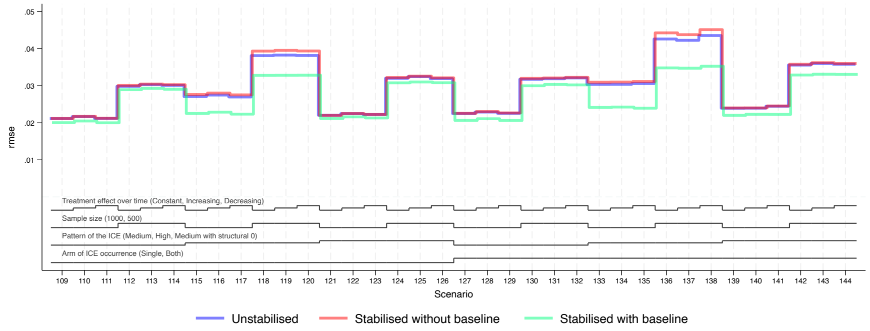

(c) Performance measure: RMSE. MCSE $\leq$ 0.001.

Figure E14. Performance measures of IPCW implementations with RC in Scenario 109 to Scenario 144 when outcome analysis model accounts for time-varying treatment effect and does covariate adjustment.

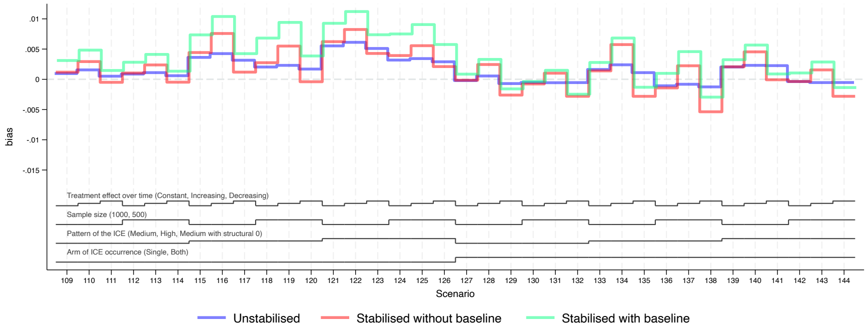

(a) Performance measure: bias. MCSE $\leq$ 0.002.

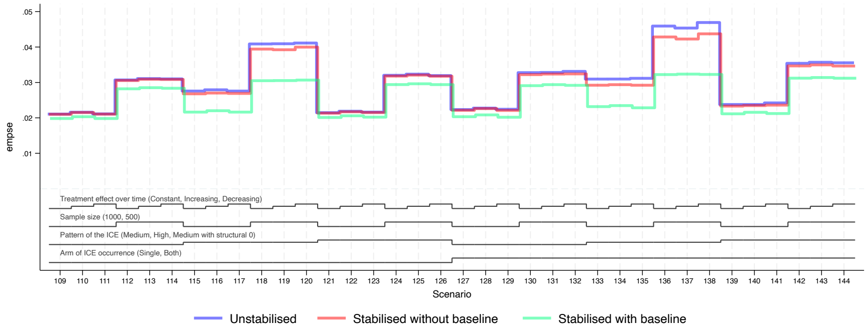

(b) Performance measure: EmpSE. MCSE $\leq$ 0.001.

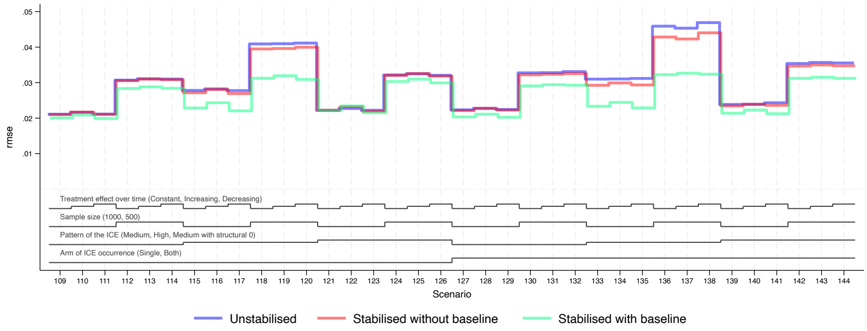

(c) Performance measure: RMSE. MCSE $\leq$ 0.001.

Figure E15. Performance measures of IPCW implementations with RC in Scenario 109 to Scenario 144 when outcome analysis model does not account for time-varying treatment effect and does not do covariate adjustment.

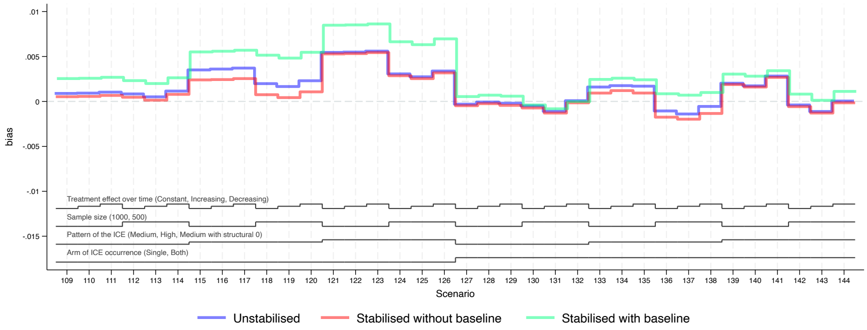

(a) Performance measure: bias. MCSE $\leq$ 0.002.

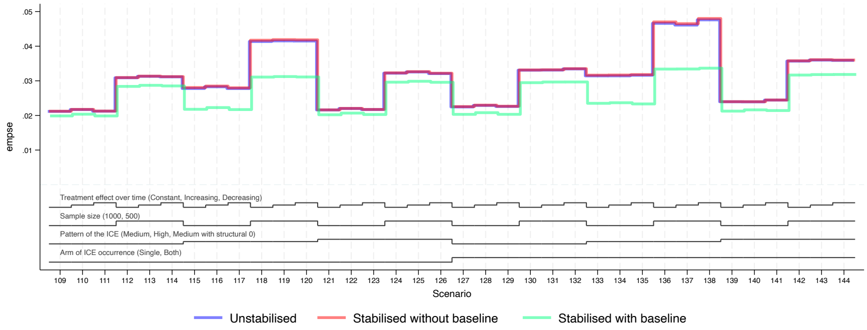

(b) Performance measure: EmpSE. MCSE $\leq$ 0.001.

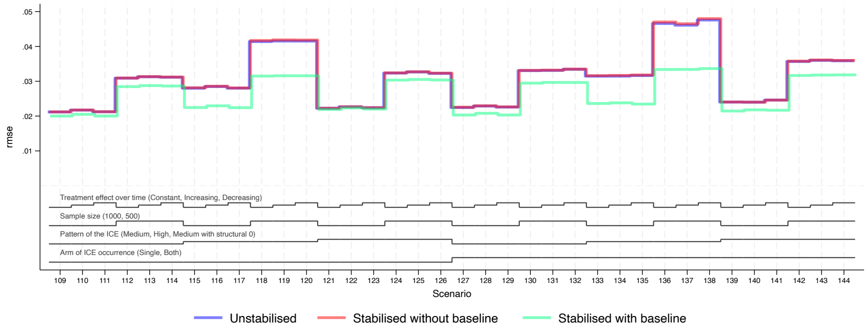

(c) Performance measure: RMSE. MCSE $\leq$ 0.001.

Figure E16. Performance measures of IPCW implementations with RC in Scenario 109 to Scenario 144 when outcome analysis model does not account for time-varying treatment effect and does not do covariate adjustment.

Appendix F Summary of weights of IPCW

F.1 Tables summarising the calculated IPW in Scenarios 1-72

Table F1. Table for summary of weights in the control arm by weighting model with NUC across scenarios where ICE occurs in the control arm.

	IPCWu_NUC			IPCWs_t_NUC			IPCWs_tL_NUC		
	Mean	SD	Max/Min	Mean	SD	Max/Min	Mean	SD	Max/Min
1	1.21	0.53	14.68	1.00	0.36	14.93	1.00	0.08	4.17
2	1.21	0.53	14.68	1.00	0.36	14.93	1.00	0.08	4.17
3	1.21	0.53	14.68	1.00	0.36	14.93	1.00	0.08	4.17
4	1.21	0.53	11.47	1.00	0.36	11.66	1.00	0.08	3.87
5	1.21	0.53	11.47	1.00	0.36	11.66	1.00	0.08	3.87
6	1.21	0.53	11.47	1.00	0.36	11.66	1.00	0.08	3.87
7	1.47	1.60	49.63	0.99	0.79	52.95	1.00	0.13	6.89
8	1.47	1.60	49.63	0.99	0.79	52.95	1.00	0.13	6.89
9	1.47	1.60	49.63	0.99	0.79	52.95	1.00	0.13	6.89
10	1.47	1.53	35.85	0.99	0.75	38.02	1.00	0.13	5.94
11	1.47	1.53	35.85	0.99	0.75	38.02	1.00	0.13	5.94
12	1.47	1.53	35.85	0.99	0.75	38.02	1.00	0.13	5.94
13	1.26	0.59	13.97	0.99	0.38	14.21	0.99	0.14	7.69
14	1.26	0.59	13.97	0.99	0.38	14.21	0.99	0.14	7.69
15	1.26	0.59	13.97	0.99	0.38	14.21	0.99	0.14	7.69
16	1.26	0.59	11.50	0.98	0.38	11.78	0.99	0.14	6.50
17	1.26	0.59	11.50	0.98	0.38	11.78	0.99	0.14	6.50
18	1.26	0.59	11.50	0.98	0.38	11.78	0.99	0.14	6.50
37	1.21	0.53	14.68	1.00	0.36	14.93	1.00	0.08	4.17
38	1.21	0.53	14.68	1.00	0.36	14.93	1.00	0.08	4.17
39	1.21	0.53	14.68	1.00	0.36	14.93	1.00	0.08	4.17
40	1.21	0.53	11.47	1.00	0.36	11.66	1.00	0.08	3.87
41	1.21	0.53	11.47	1.00	0.36	11.66	1.00	0.08	3.87
42	1.21	0.53	11.47	1.00	0.36	11.66	1.00	0.08	3.87
43	1.47	1.60	49.63	0.99	0.79	52.95	1.00	0.13	6.89
44	1.47	1.60	49.63	0.99	0.79	52.95	1.00	0.13	6.89
45	1.47	1.60	49.63	0.99	0.79	52.95	1.00	0.13	6.89
46	1.47	1.53	35.85	0.99	0.75	38.02	1.00	0.13	5.94
47	1.47	1.53	35.85	0.99	0.75	38.02	1.00	0.13	5.94
48	1.47	1.53	35.85	0.99	0.75	38.02	1.00	0.13	5.94
49	1.26	0.59	13.97	0.99	0.38	14.21	0.99	0.14	7.69
50	1.26	0.59	13.97	0.99	0.38	14.21	0.99	0.14	7.69
51	1.26	0.59	13.97	0.99	0.38	14.21	0.99	0.14	7.69
52	1.26	0.59	11.50	0.98	0.38	11.78	0.99	0.14	6.50
53	1.26	0.59	11.50	0.98	0.38	11.78	0.99	0.14	6.50
54	1.26	0.59	11.50	0.98	0.38	11.78	0.99	0.14	6.50

Table F2. Table for summary of weights in the control arm by weighting model with RC across scenarios where ICE occurs in the control arm.

	IPCWu_NUC			IPCWs_t_NUC			IPCWs_tL_NUC		
	Mean	SD	Max/Min	Mean	SD	Max/Min	Mean	SD	Max/Min
1	1.21	0.53	14.34	1.00	0.36	14.56	1.00	0.06	3.14
2	1.21	0.53	14.34	1.00	0.36	14.56	1.00	0.06	3.14
3	1.21	0.53	14.34	1.00	0.36	14.56	1.00	0.06	3.14
4	1.21	0.53	11.59	1.00	0.36	11.75	1.00	0.06	2.88
5	1.21	0.53	11.59	1.00	0.36	11.75	1.00	0.06	2.88
6	1.21	0.53	11.59	1.00	0.36	11.75	1.00	0.06	2.88
7	1.47	1.57	49.09	0.99	0.77	52.29	1.00	0.10	4.75
8	1.47	1.57	49.09	0.99	0.77	52.29	1.00	0.10	4.75
9	1.47	1.57	49.09	0.99	0.77	52.29	1.00	0.10	4.75
10	1.47	1.54	36.61	0.99	0.75	38.66	1.00	0.10	4.13
11	1.47	1.54	36.61	0.99	0.75	38.66	1.00	0.10	4.13
12	1.47	1.54	36.61	0.99	0.75	38.66	1.00	0.10	4.13
13	1.27	0.61	14.60	0.99	0.39	14.81	1.00	0.13	6.38
14	1.27	0.61	14.60	0.99	0.39	14.81	1.00	0.13	6.38
15	1.27	0.61	14.60	0.99	0.39	14.81	1.00	0.13	6.38
16	1.27	0.60	11.60	0.99	0.38	11.82	1.00	0.13	5.23
17	1.27	0.60	11.60	0.99	0.38	11.82	1.00	0.13	5.23
18	1.27	0.60	11.60	0.99	0.38	11.82	1.00	0.13	5.23
37	1.21	0.53	14.34	1.00	0.36	14.56	1.00	0.06	3.14
38	1.21	0.53	14.34	1.00	0.36	14.56	1.00	0.06	3.14
39	1.21	0.53	14.34	1.00	0.36	14.56	1.00	0.06	3.14
40	1.21	0.53	11.59	1.00	0.36	11.75	1.00	0.06	2.88
41	1.21	0.53	11.59	1.00	0.36	11.75	1.00	0.06	2.88
42	1.21	0.53	11.59	1.00	0.36	11.75	1.00	0.06	2.88
43	1.47	1.57	49.09	0.99	0.77	52.29	1.00	0.10	4.75
44	1.47	1.57	49.09	0.99	0.77	52.29	1.00	0.10	4.75
45	1.47	1.57	49.09	0.99	0.77	52.29	1.00	0.10	4.75
46	1.47	1.54	36.61	0.99	0.75	38.66	1.00	0.10	4.13
47	1.47	1.54	36.61	0.99	0.75	38.66	1.00	0.10	4.13
48	1.47	1.54	36.61	0.99	0.75	38.66	1.00	0.10	4.13
49	1.27	0.61	14.60	0.99	0.39	14.81	1.00	0.13	6.38
50	1.27	0.61	14.60	0.99	0.39	14.81	1.00	0.13	6.38
51	1.27	0.61	14.60	0.99	0.39	14.81	1.00	0.13	6.38
52	1.27	0.60	11.60	0.99	0.38	11.82	1.00	0.13	5.23
53	1.27	0.60	11.60	0.99	0.38	11.82	1.00	0.13	5.23
54	1.27	0.60	11.60	0.99	0.38	11.82	1.00	0.13	5.23

Table F3. Table for summary of weights in the control arm by weighting model with NUC across scenarios where ICE occurs in both arms.

	IPCWu_NUC			IPCWs_t_NUC			IPCWs_tL_NUC		
	Mean	SD	Max/Min	Mean	SD	Max/Min	Mean	SD	Max/Min
19	1.21	0.53	14.68	1.00	0.36	14.93	1.00	0.08	4.17
20	1.21	0.53	14.68	1.00	0.36	14.93	1.00	0.08	4.17
21	1.21	0.53	14.68	1.00	0.36	14.93	1.00	0.08	4.17
22	1.21	0.53	11.47	1.00	0.36	11.66	1.00	0.08	3.87
23	1.21	0.53	11.47	1.00	0.36	11.66	1.00	0.08	3.87
24	1.21	0.53	11.47	1.00	0.36	11.66	1.00	0.08	3.87
25	1.47	1.60	49.63	0.99	0.79	52.95	1.00	0.13	6.89
26	1.47	1.60	49.63	0.99	0.79	52.95	1.00	0.13	6.89
27	1.47	1.60	49.63	0.99	0.79	52.95	1.00	0.13	6.89
28	1.47	1.53	35.85	0.99	0.75	38.02	1.00	0.13	5.94
29	1.47	1.53	35.85	0.99	0.75	38.02	1.00	0.13	5.94
30	1.47	1.53	35.85	0.99	0.75	38.02	1.00	0.13	5.94
31	1.26	0.59	13.97	0.99	0.38	14.21	0.99	0.14	7.69
32	1.26	0.59	13.97	0.99	0.38	14.21	0.99	0.14	7.69
33	1.26	0.59	13.97	0.99	0.38	14.21	0.99	0.14	7.69
34	1.26	0.59	11.50	0.98	0.38	11.78	0.99	0.14	6.50
35	1.26	0.59	11.50	0.98	0.38	11.78	0.99	0.14	6.50
36	1.26	0.59	11.50	0.98	0.38	11.78	0.99	0.14	6.50
55	1.21	0.53	14.68	1.00	0.36	14.93	1.00	0.08	4.17
56	1.21	0.53	14.68	1.00	0.36	14.93	1.00	0.08	4.17
57	1.21	0.53	14.68	1.00	0.36	14.93	1.00	0.08	4.17
58	1.21	0.53	11.47	1.00	0.36	11.66	1.00	0.08	3.87
59	1.21	0.53	11.47	1.00	0.36	11.66	1.00	0.08	3.87
60	1.21	0.53	11.47	1.00	0.36	11.66	1.00	0.08	3.87
61	1.47	1.60	49.63	0.99	0.79	52.95	1.00	0.13	6.89
62	1.47	1.60	49.63	0.99	0.79	52.95	1.00	0.13	6.89
63	1.47	1.60	49.63	0.99	0.79	52.95	1.00	0.13	6.89
64	1.47	1.53	35.85	0.99	0.75	38.02	1.00	0.13	5.94
65	1.47	1.53	35.85	0.99	0.75	38.02	1.00	0.13	5.94
66	1.47	1.53	35.85	0.99	0.75	38.02	1.00	0.13	5.94
67	1.26	0.59	13.97	0.99	0.38	14.21	0.99	0.14	7.69
68	1.26	0.59	13.97	0.99	0.38	14.21	0.99	0.14	7.69
69	1.26	0.59	13.97	0.99	0.38	14.21	0.99	0.14	7.69
70	1.26	0.59	11.50	0.98	0.38	11.78	0.99	0.14	6.50
71	1.26	0.59	11.50	0.98	0.38	11.78	0.99	0.14	6.50
72	1.26	0.59	11.50	0.98	0.38	11.78	0.99	0.14	6.50

Table F4. Table for summary of weights in the experimental arm by weighting model with NUC across scenarios where ICE occurs in both arms.

	IPCWu_NUC			IPCWs_t_NUC			IPCWs_tL_NUC		
	Mean	SD	Max/Min	Mean	SD	Max/Min	Mean	SD	Max/Min
19	1.21	0.50	13.57	1.00	0.35	13.76	1.00	0.08	4.06
20	1.20	0.50	13.52	1.00	0.35	13.71	1.00	0.08	4.06
21	1.21	0.50	13.65	1.00	0.35	13.84	1.00	0.08	4.06
22	1.21	0.51	11.25	1.00	0.35	11.45	1.00	0.08	3.70
23	1.21	0.51	11.18	1.00	0.35	11.38	1.00	0.08	3.69
24	1.21	0.51	11.32	1.00	0.35	11.53	1.00	0.08	3.70
25	1.46	1.51	47.29	1.00	0.77	49.76	1.00	0.12	6.74
26	1.46	1.50	46.68	0.99	0.76	49.18	1.00	0.12	6.72
27	1.46	1.51	47.40	1.00	0.77	49.88	1.00	0.12	6.80
28	1.46	1.61	40.47	1.00	0.82	42.42	1.00	0.13	5.90
29	1.46	1.62	40.85	1.00	0.82	42.81	1.00	0.13	5.92
30	1.46	1.63	41.20	1.00	0.83	43.16	1.00	0.13	5.90
31	1.26	0.57	13.29	0.99	0.37	13.49	0.99	0.14	7.44
32	1.26	0.57	13.27	0.99	0.37	13.47	0.99	0.14	7.40
33	1.26	0.57	13.32	0.99	0.37	13.53	0.99	0.14	7.45
34	1.26	0.57	11.05	0.99	0.37	11.24	1.00	0.14	6.32
35	1.25	0.57	11.01	0.99	0.37	11.20	1.00	0.14	6.32
36	1.26	0.57	11.10	0.99	0.37	11.29	1.00	0.14	6.34
55	1.20	0.49	13.40	1.00	0.34	13.59	1.00	0.07	4.02
56	1.20	0.49	13.30	1.00	0.34	13.48	1.00	0.07	3.99
57	1.20	0.49	13.38	1.00	0.34	13.57	1.00	0.08	4.04
58	1.20	0.50	11.05	1.00	0.35	11.25	1.00	0.08	3.66
59	1.20	0.50	10.94	1.00	0.35	11.14	1.00	0.08	3.66
60	1.20	0.50	11.13	1.00	0.35	11.35	1.00	0.08	3.69
61	1.45	1.48	45.99	0.99	0.75	48.43	1.00	0.12	6.69
62	1.45	1.47	45.67	0.99	0.75	48.13	1.00	0.12	6.66
63	1.45	1.49	46.19	0.99	0.76	48.69	1.00	0.12	6.72
64	1.46	1.60	40.14	1.00	0.81	42.09	1.00	0.13	5.86
65	1.45	1.58	39.42	1.00	0.80	41.34	1.00	0.13	5.83
66	1.46	1.61	40.57	1.00	0.82	42.53	1.00	0.13	5.88
67	1.25	0.56	13.09	0.99	0.36	13.30	0.99	0.14	7.35
68	1.25	0.56	13.06	0.99	0.36	13.26	0.99	0.13	7.34
69	1.25	0.56	13.11	0.99	0.36	13.33	0.99	0.14	7.38
70	1.25	0.56	10.91	0.99	0.37	11.11	0.99	0.14	6.31
71	1.25	0.56	10.88	0.99	0.36	11.07	0.99	0.14	6.28
72	1.25	0.57	10.95	0.99	0.37	11.15	1.00	0.14	6.32

Table F5. Table for summary of weights in the control arm by weighting model with RC across scenarios where ICE occurs in both arms.

	IPCWu_NUC			IPCWs_t_NUC			IPCWs_tL_NUC		
	Mean	SD	Max/Min	Mean	SD	Max/Min	Mean	SD	Max/Min
19	1.21	0.53	14.34	1.00	0.36	14.56	1.00	0.06	3.14
20	1.21	0.53	14.34	1.00	0.36	14.56	1.00	0.06	3.14
21	1.21	0.53	14.34	1.00	0.36	14.56	1.00	0.06	3.14
22	1.21	0.53	11.59	1.00	0.36	11.75	1.00	0.06	2.88
23	1.21	0.53	11.59	1.00	0.36	11.75	1.00	0.06	2.88
24	1.21	0.53	11.59	1.00	0.36	11.75	1.00	0.06	2.88
25	1.47	1.57	49.09	0.99	0.77	52.29	1.00	0.10	4.75
26	1.47	1.57	49.09	0.99	0.77	52.29	1.00	0.10	4.75
27	1.47	1.57	49.09	0.99	0.77	52.29	1.00	0.10	4.75
28	1.47	1.54	36.61	0.99	0.75	38.66	1.00	0.10	4.13
29	1.47	1.54	36.61	0.99	0.75	38.66	1.00	0.10	4.13
30	1.47	1.54	36.61	0.99	0.75	38.66	1.00	0.10	4.13
31	1.27	0.61	14.60	0.99	0.39	14.81	1.00	0.13	6.38
32	1.27	0.61	14.60	0.99	0.39	14.81	1.00	0.13	6.38
33	1.27	0.61	14.60	0.99	0.39	14.81	1.00	0.13	6.38
34	1.27	0.60	11.60	0.99	0.38	11.82	1.00	0.13	5.23
35	1.27	0.60	11.60	0.99	0.38	11.82	1.00	0.13	5.23
36	1.27	0.60	11.60	0.99	0.38	11.82	1.00	0.13	5.23
55	1.21	0.53	14.34	1.00	0.36	14.56	1.00	0.06	3.14
56	1.21	0.53	14.34	1.00	0.36	14.56	1.00	0.06	3.14
57	1.21	0.53	14.34	1.00	0.36	14.56	1.00	0.06	3.14
58	1.21	0.53	11.59	1.00	0.36	11.75	1.00	0.06	2.88
59	1.21	0.53	11.59	1.00	0.36	11.75	1.00	0.06	2.88
60	1.21	0.53	11.59	1.00	0.36	11.75	1.00	0.06	2.88
61	1.47	1.57	49.09	0.99	0.77	52.29	1.00	0.10	4.75
62	1.47	1.57	49.09	0.99	0.77	52.29	1.00	0.10	4.75
63	1.47	1.57	49.09	0.99	0.77	52.29	1.00	0.10	4.75
64	1.47	1.54	36.61	0.99	0.75	38.66	1.00	0.10	4.13
65	1.47	1.54	36.61	0.99	0.75	38.66	1.00	0.10	4.13
66	1.47	1.54	36.61	0.99	0.75	38.66	1.00	0.10	4.13
67	1.27	0.61	14.60	0.99	0.39	14.81	1.00	0.13	6.38
68	1.27	0.61	14.60	0.99	0.39	14.81	1.00	0.13	6.38
69	1.27	0.61	14.60	0.99	0.39	14.81	1.00	0.13	6.38
70	1.27	0.60	11.60	0.99	0.38	11.82	1.00	0.13	5.23
71	1.27	0.60	11.60	0.99	0.38	11.82	1.00	0.13	5.23
72	1.27	0.60	11.60	0.99	0.38	11.82	1.00	0.13	5.23

Table F6. Table for summary of weights in the control arm by weighting model with RC across scenarios where ICE occurs in both arms.

	IPCWu_NUC			IPCWs_t_NUC			IPCWs_tL_NUC		
	Mean	SD	Max/Min	Mean	SD	Max/Min	Mean	SD	Max/Min
19	1.20	0.49	13.14	1.00	0.34	13.29	1.00	0.06	3.02
20	1.20	0.48	13.05	1.00	0.34	13.20	1.00	0.06	3.00
21	1.20	0.49	13.13	1.00	0.34	13.29	1.00	0.06	3.03
22	1.20	0.50	10.89	1.00	0.34	11.07	1.00	0.06	2.78
23	1.20	0.50	10.85	1.00	0.34	11.02	1.00	0.06	2.77
24	1.20	0.50	10.99	1.00	0.35	11.18	1.00	0.06	2.80
25	1.45	1.48	46.34	1.00	0.75	48.56	1.00	0.10	4.61
26	1.45	1.47	45.79	0.99	0.74	48.01	1.00	0.10	4.59
27	1.45	1.48	46.54	1.00	0.75	48.81	1.00	0.10	4.63
28	1.46	1.56	38.55	1.00	0.79	40.30	1.00	0.10	4.08
29	1.46	1.55	38.35	1.00	0.78	40.06	1.00	0.10	4.07
30	1.46	1.57	39.03	1.00	0.79	40.80	1.00	0.10	4.08
31	1.26	0.56	13.03	0.99	0.36	13.19	1.00	0.12	5.91
32	1.26	0.56	12.97	0.99	0.36	13.13	1.00	0.12	5.89
33	1.26	0.56	13.06	1.00	0.36	13.23	1.00	0.12	5.92
34	1.26	0.56	10.57	0.99	0.36	10.72	1.00	0.12	5.00
35	1.26	0.56	10.55	0.99	0.36	10.70	1.00	0.12	4.98
36	1.26	0.56	10.59	0.99	0.36	10.74	1.00	0.12	5.02
55	1.21	0.49	13.27	1.00	0.34	13.42	1.00	0.06	3.05
56	1.21	0.49	13.24	1.00	0.34	13.39	1.00	0.06	3.04
57	1.21	0.49	13.34	1.00	0.34	13.49	1.00	0.06	3.05
58	1.21	0.50	11.13	1.00	0.35	11.30	1.00	0.06	2.81
59	1.21	0.50	11.05	1.00	0.35	11.22	1.00	0.06	2.80
60	1.21	0.51	11.20	1.00	0.35	11.38	1.00	0.06	2.81
61	1.46	1.50	47.35	1.00	0.76	49.60	1.00	0.10	4.65
62	1.46	1.49	46.92	1.00	0.76	49.18	1.00	0.10	4.64
63	1.46	1.50	47.40	1.00	0.76	49.66	1.00	0.10	4.67
64	1.46	1.57	38.94	1.00	0.79	40.69	1.00	0.10	4.09
65	1.46	1.57	39.03	1.00	0.79	40.81	1.00	0.10	4.09
66	1.47	1.58	39.38	1.00	0.80	41.17	1.00	0.10	4.09
67	1.26	0.57	13.22	1.00	0.37	13.38	1.00	0.12	5.95
68	1.26	0.57	13.19	1.00	0.37	13.34	1.00	0.12	5.94
69	1.26	0.57	13.25	1.00	0.37	13.41	1.00	0.12	5.96
70	1.26	0.57	10.68	0.99	0.37	10.82	1.00	0.13	5.02
71	1.26	0.57	10.66	0.99	0.36	10.80	1.00	0.13	5.02
72	1.26	0.57	10.71	0.99	0.37	10.85	1.00	0.13	5.04

F.2 Boxplots of SD and Min/Max of calculated IPW in Scenarios 1-72

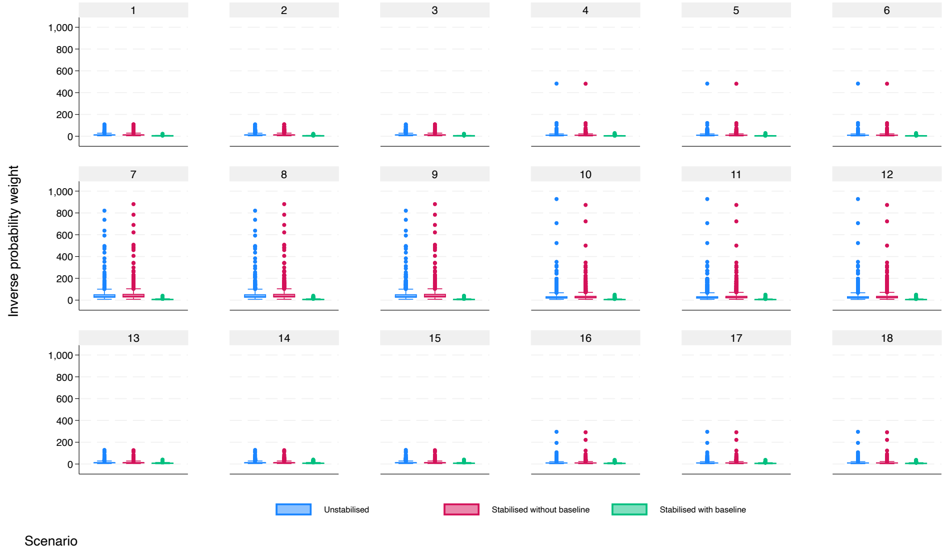

(a) Max/Min of IPW in the control arm.

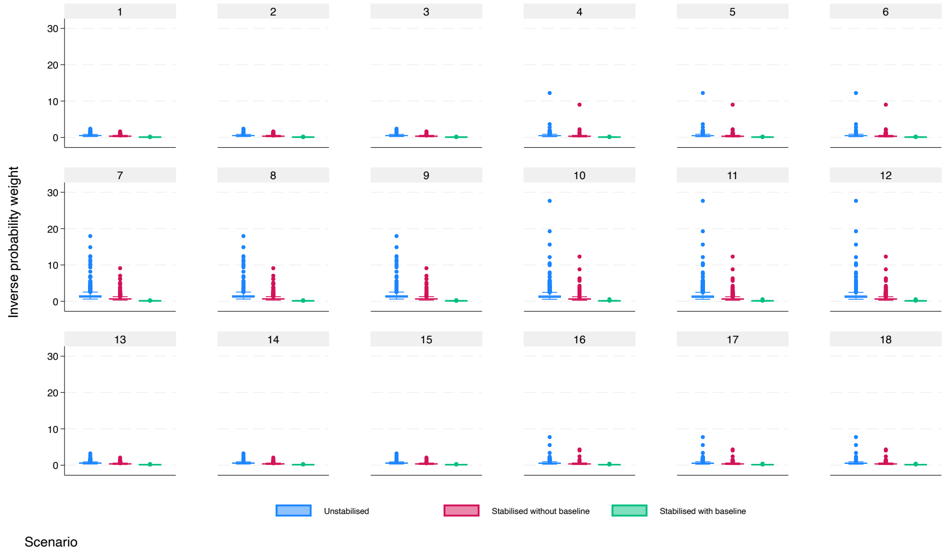

(b) SD of IPW in the control arm.

Figure F1. IPW by different weighting models with NUC in the control arm in Scenario 1-18.

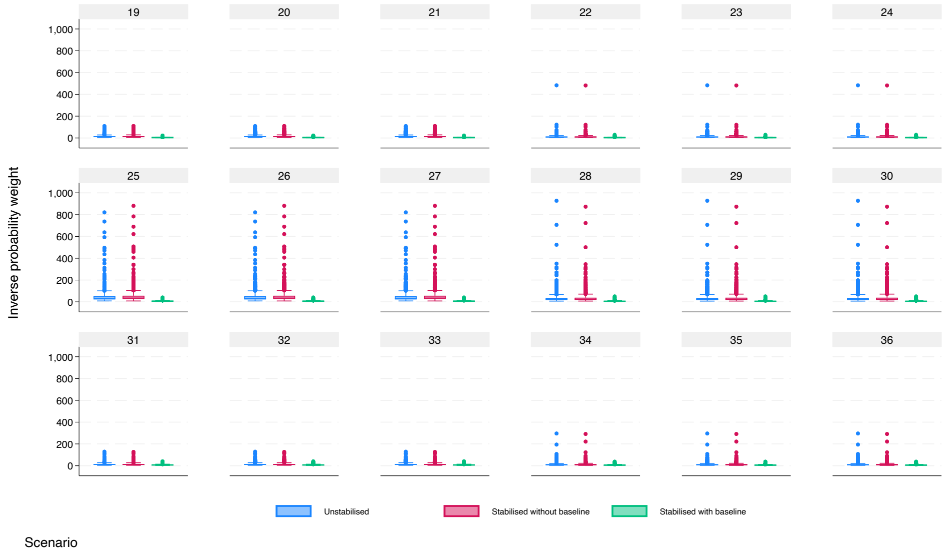

(a) Max/Min of IPW in the control arm.

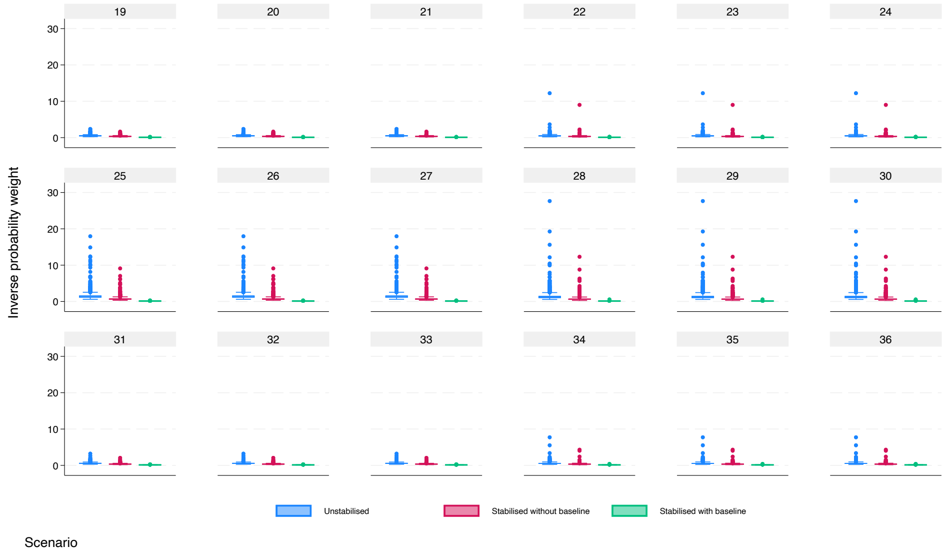

(b) SD of IPW in the control arm.

Figure F2. IPW by different weighting models with NUC in the control arm in Scenario 19-36.

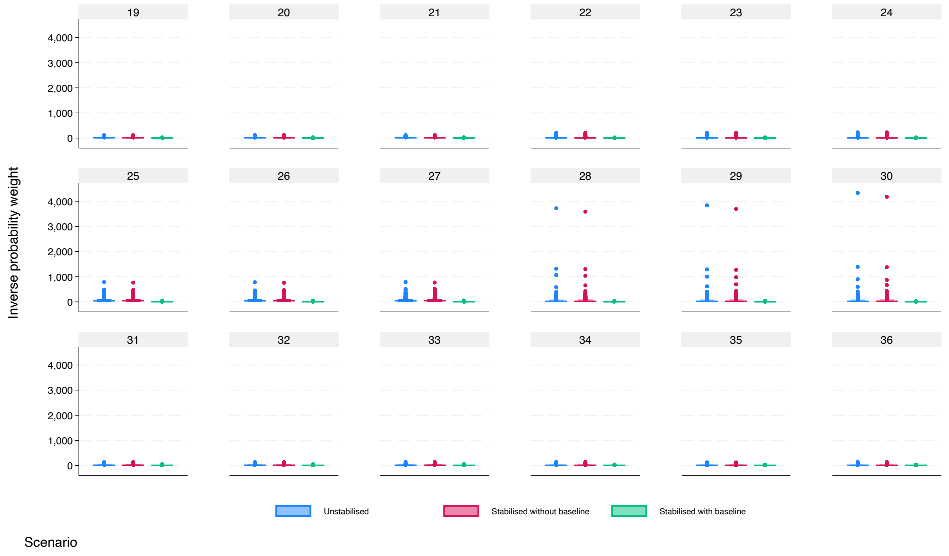

(a) Max/Min of IPW in the experimental arm.

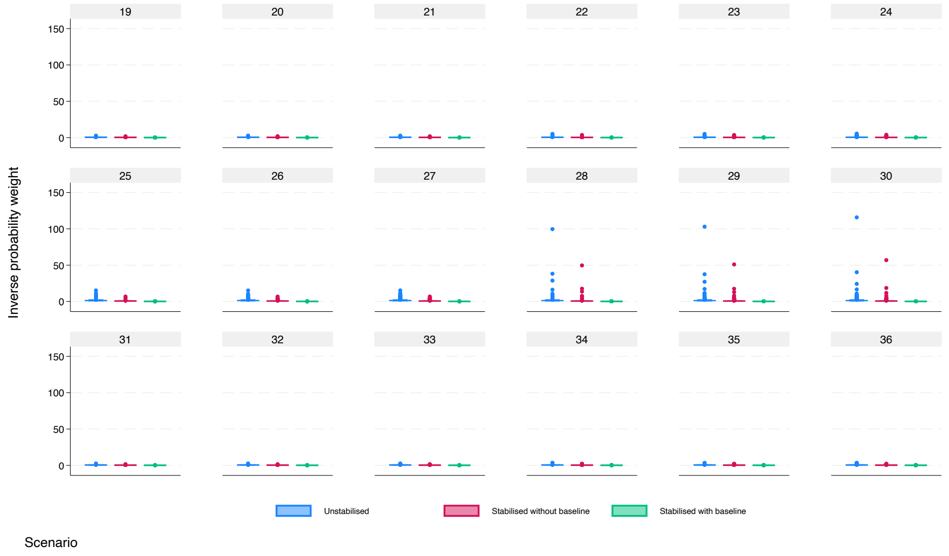

(b) SD of IPW in the experimental arm.

Figure F3. IPW by different weighting models with NUC in the experimental arm in Scenario 19-36.

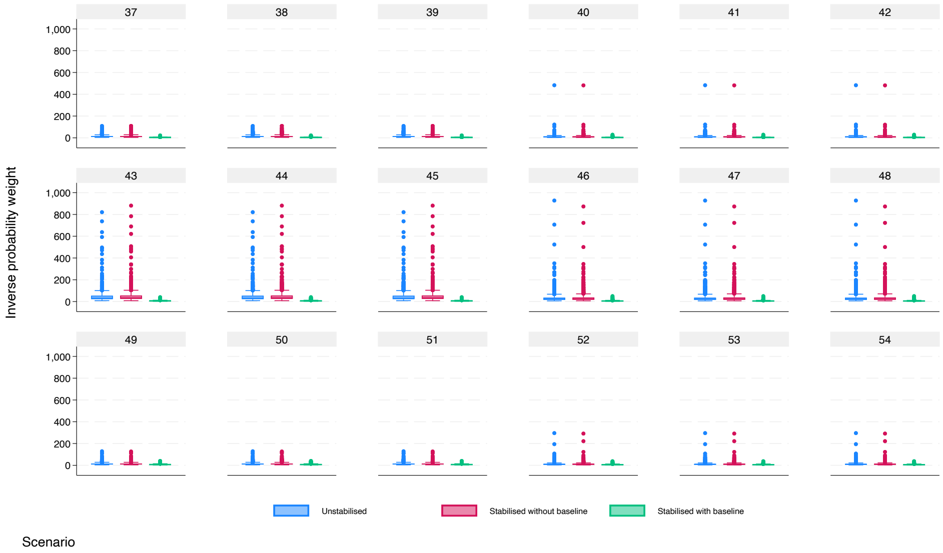

(a) Max/Min of IPW in the control arm.

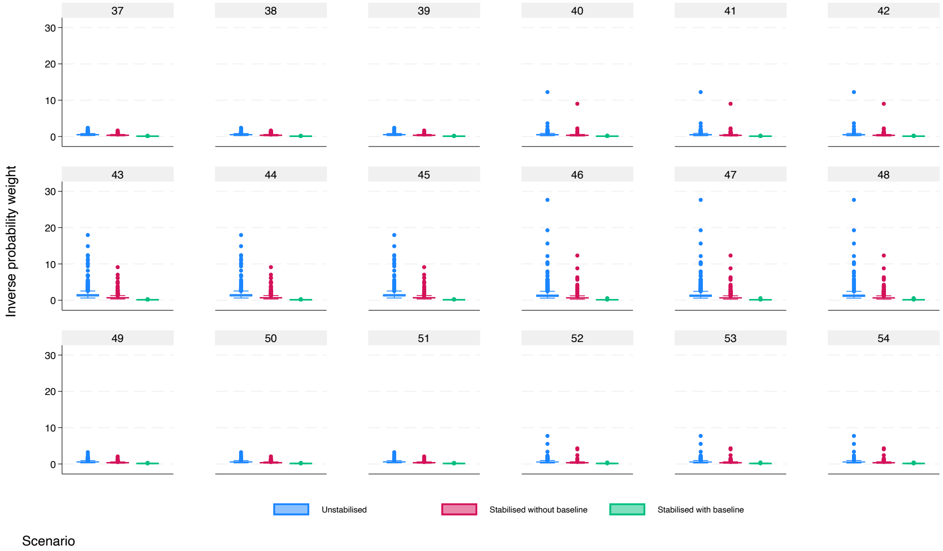

(b) SD of IPW in the control arm.

Figure F4. IPW by different weighting models with NUC in the control arm in Scenario 37-54.

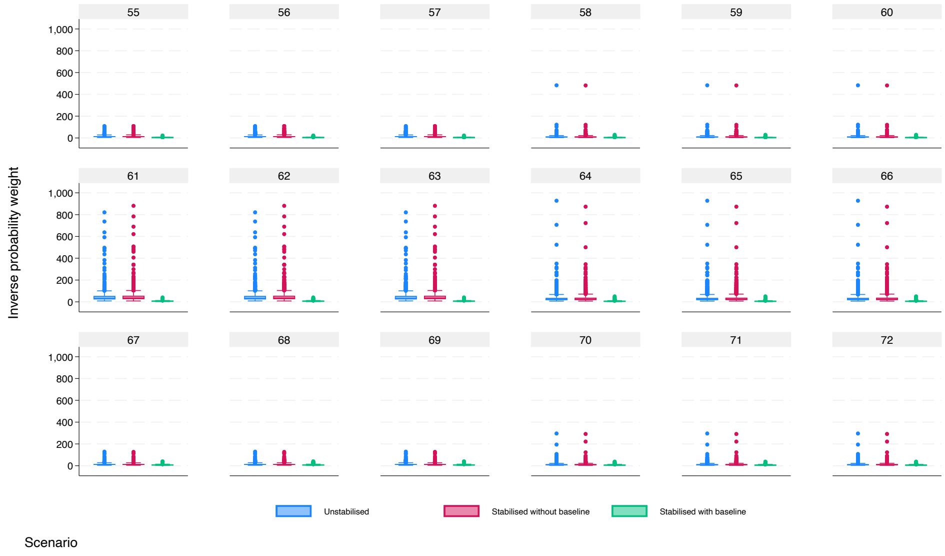

(a) Max/Min of IPW in the control arm.

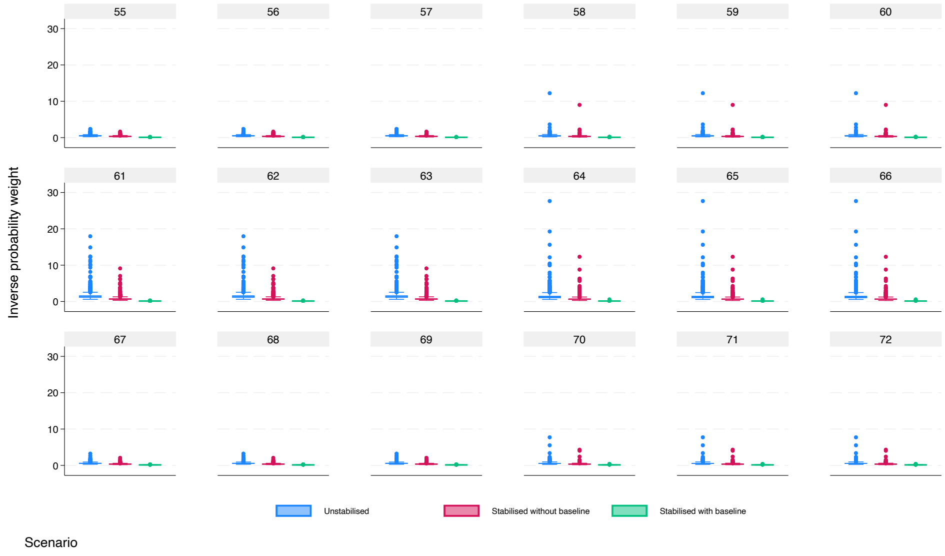

(b) SD of IPW in the control arm.

Figure F5. IPW by different weighting models with NUC in the control arm in Scenario 55-72.

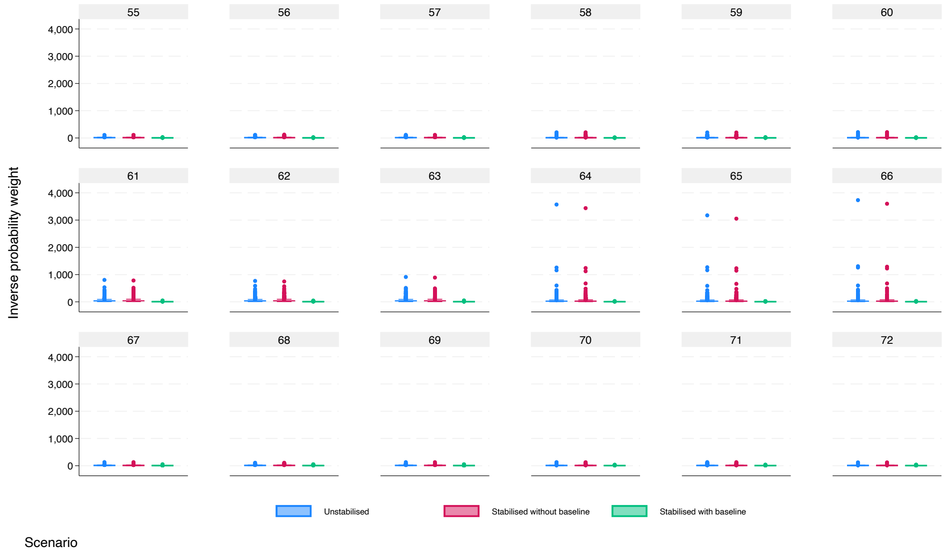

(a) Max/Min of IPW in the experimental arm.

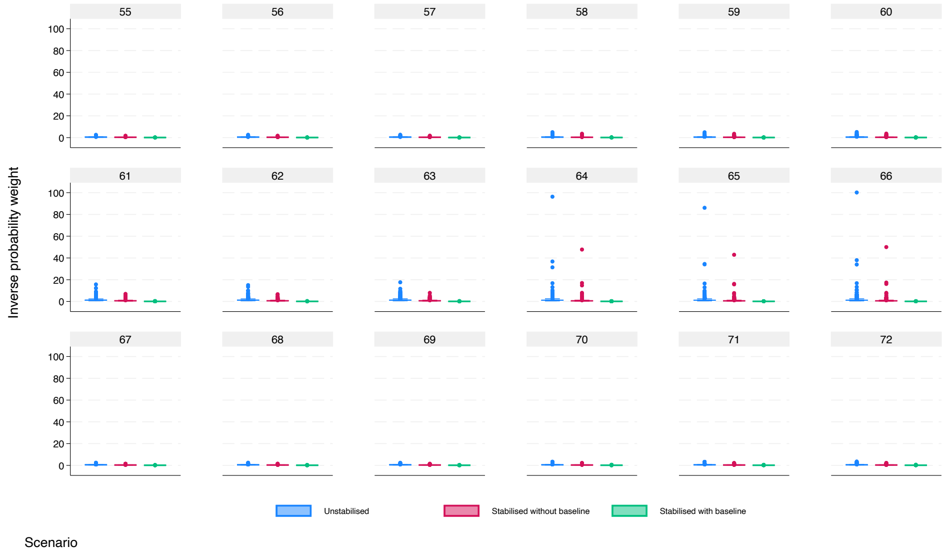

(b) SD of IPW in the experimental arm.

Figure F6. IPW by different weighting models with NUC in the experimental arm in Scenario 55-72.

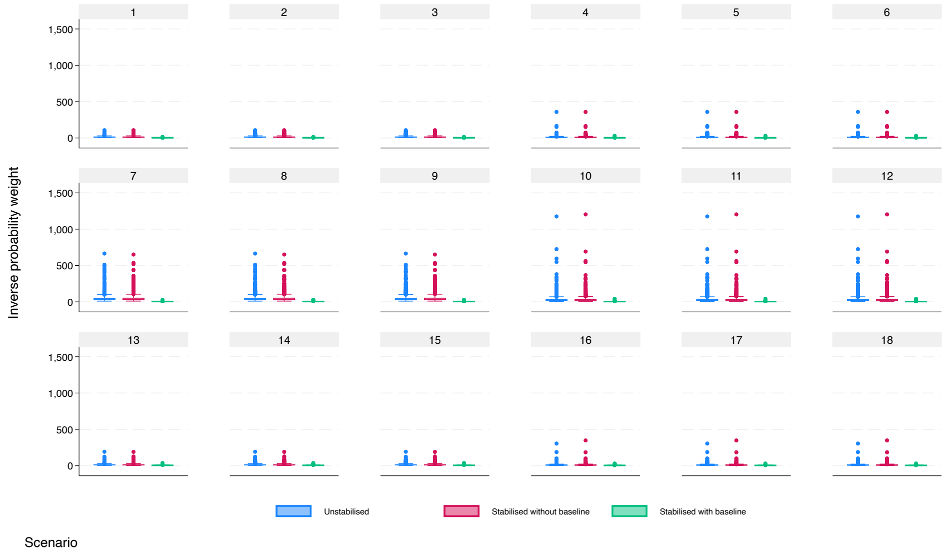

(a) Max/Min of IPW in the control arm.

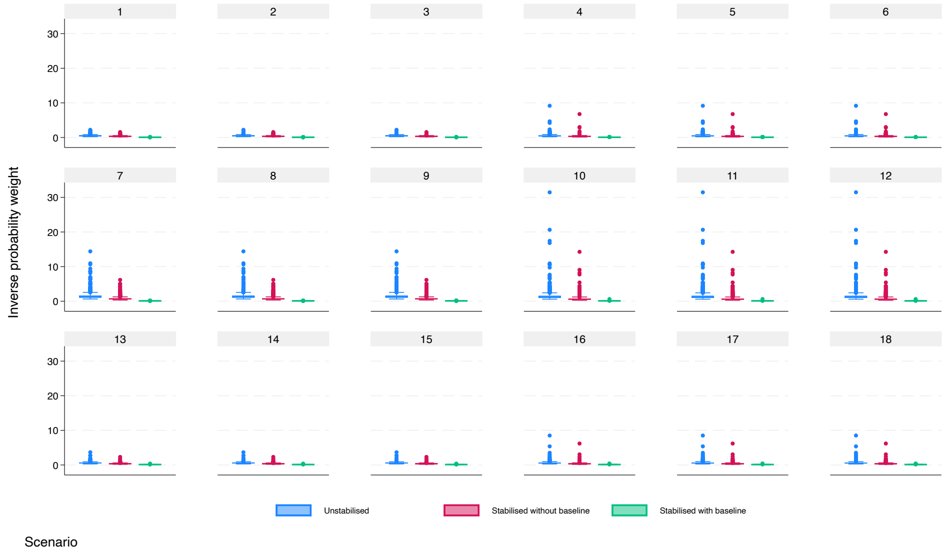

(b) SD of IPW in the control arm.

Figure F7. IPW by different weighting models with RC in the control arm in Scenario 1-18.

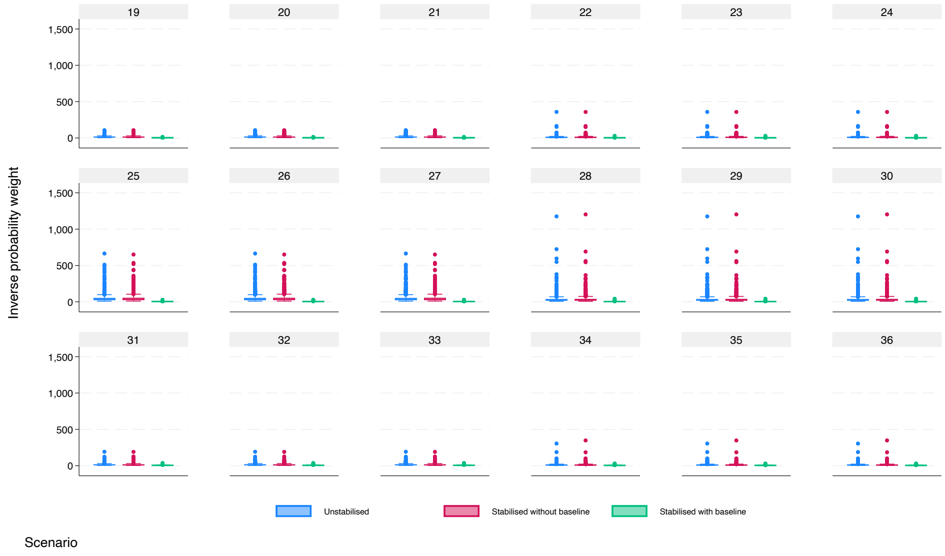

(a) Max/Min of IPW in the control arm.

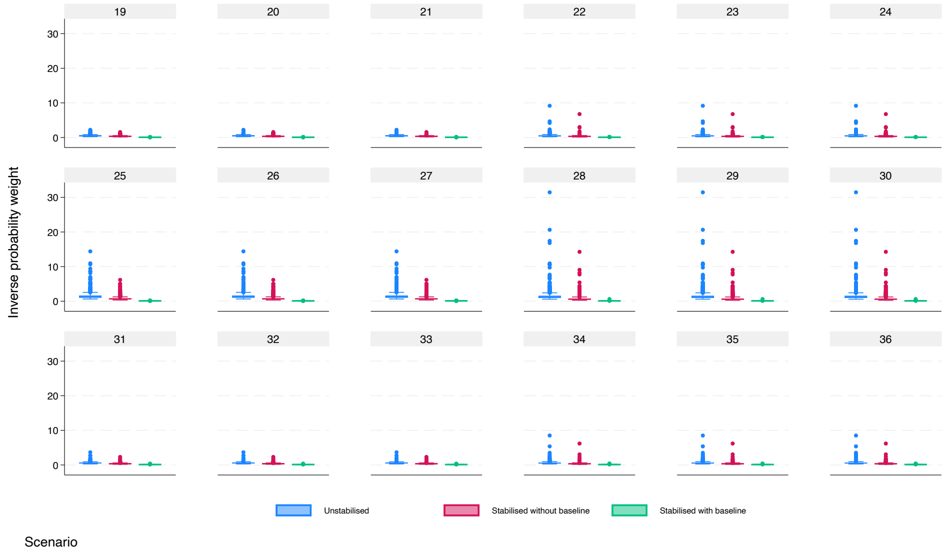

(b) SD of IPW in the control arm.

Figure F8. IPW by different weighting models with RC in the control arm in Scenario 19-36.

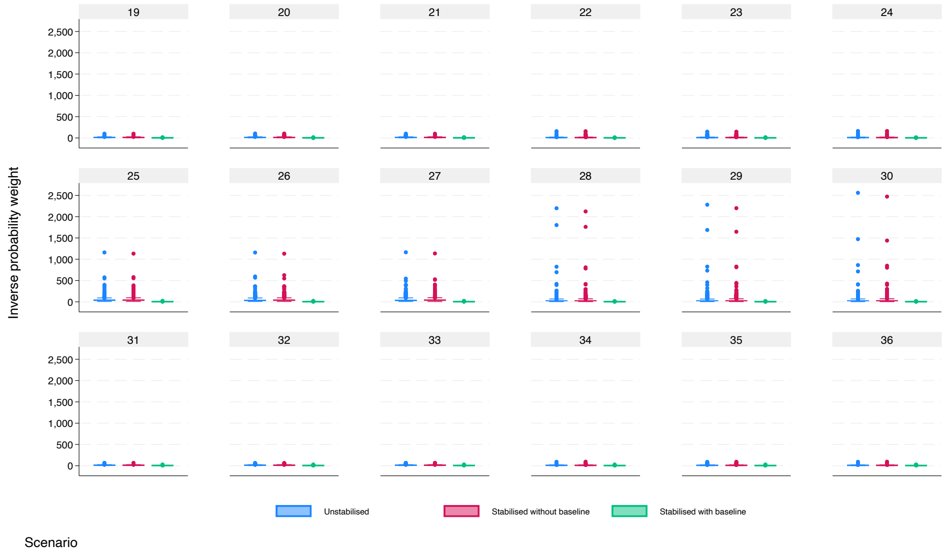

(a) Max/Min of IPW in the experimental arm.

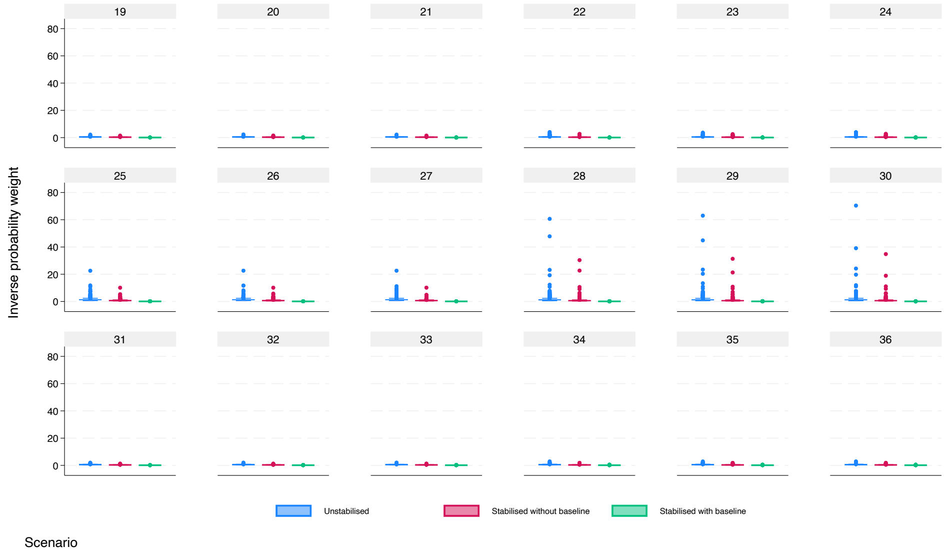

(b) SD of IPW in the experimental arm.

Figure F9. IPW by different weighting models with RC in the experimental arm in Scenario 19-36.

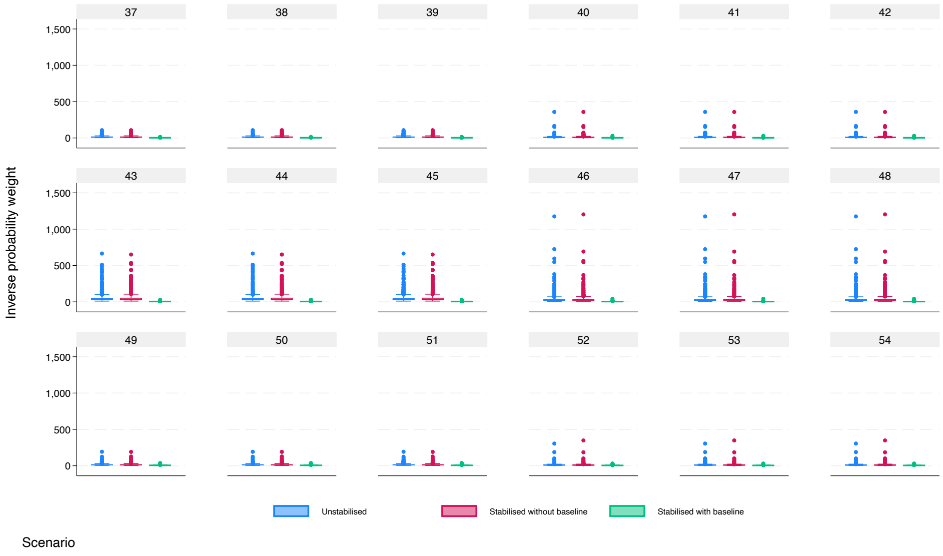

(a) Max/Min of IPW in the control arm.

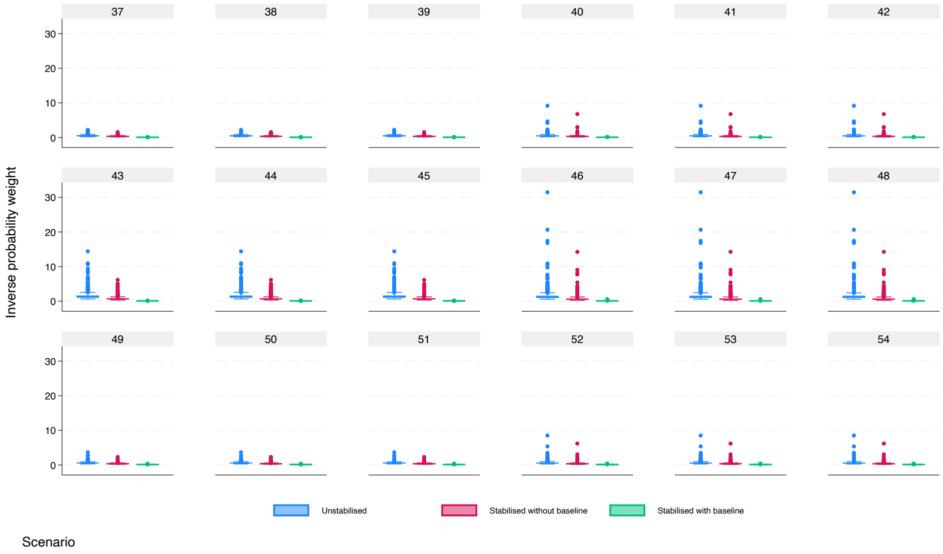

(b) SD of IPW in the control arm.

Figure F10. IPW by different weighting models with RC in the control arm in Scenario 37-54.

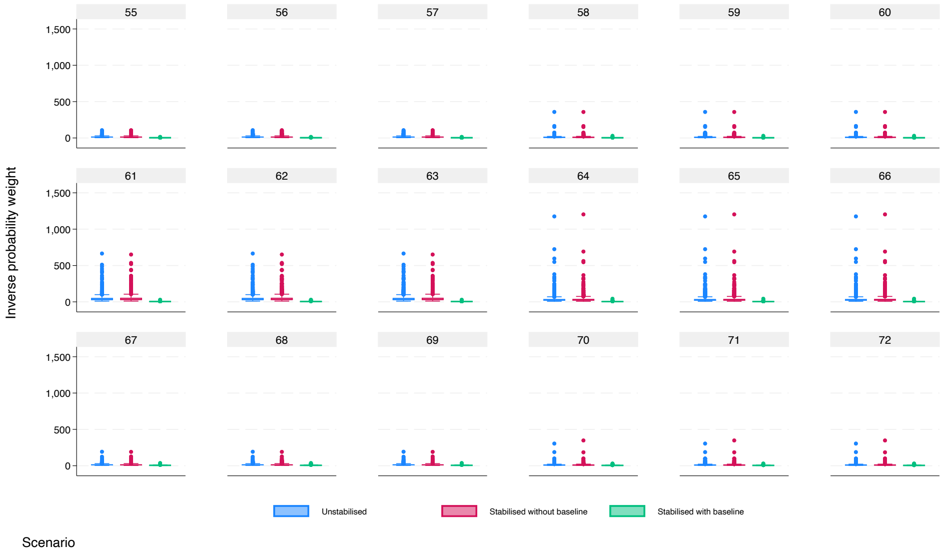

(a) Max/Min of IPW in the control arm.

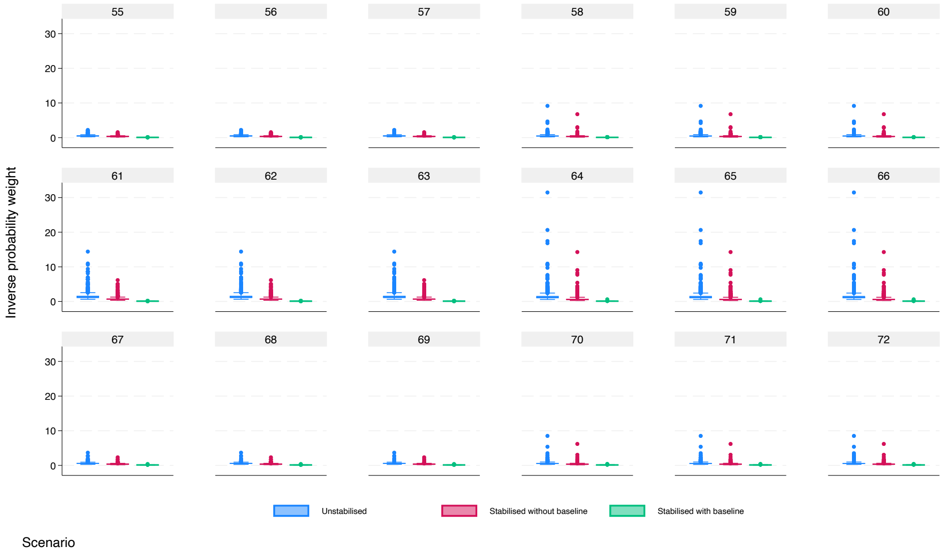

(b) SD of IPW in the control arm.

Figure F11. IPW by different weighting models with RC in the control arm in Scenario 55-72.

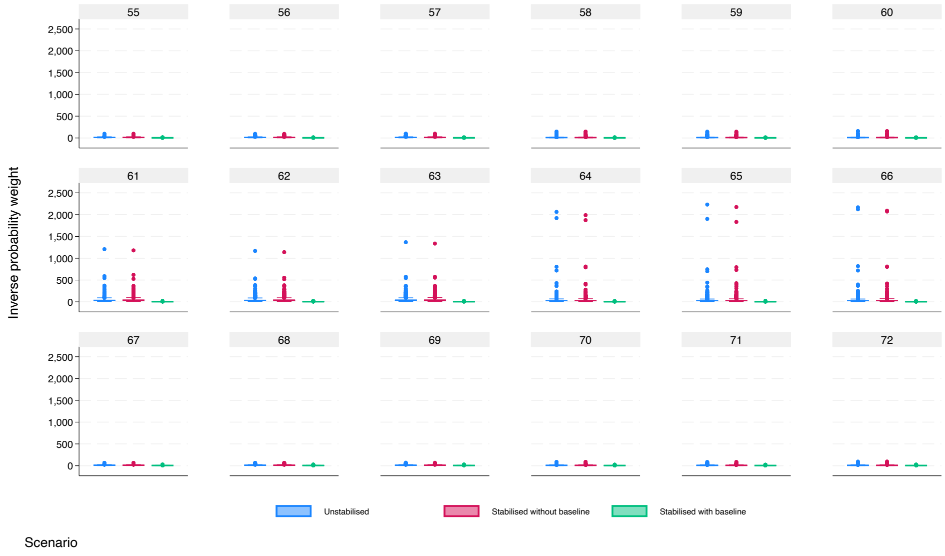

(a) Max/Min of IPW in the experimental arm.

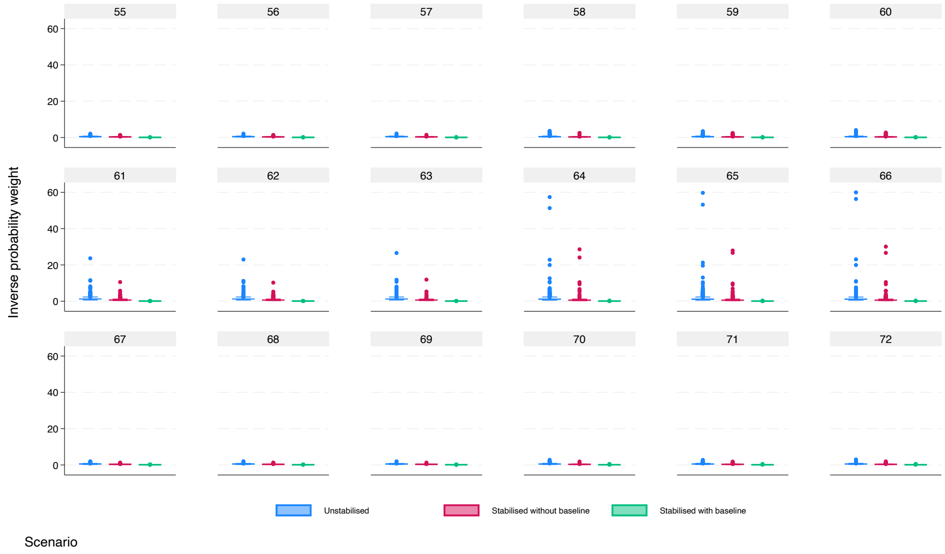

(b) SD of IPW in the experimental arm.

Figure F12. IPW by different weighting models with RC in the experimental arm in Scenario 55-72.
